# Atmospheric Removal of Nitrous Oxide: Approaches and Feasibility

**DOI:** 10.1021/acs.est.6c04107

**Published:** 2026-10-16

**Authors:** William J. Sawyer

**Affiliations:** Resolute Methane, Somerville, Massachusetts 02143, United States

**Keywords:** Non-CO_2_ greenhouse gas mitigation, Greenhouse gas removal, Nitrous oxide destruction, Feasibility assessment, Climate mitigation pathways, Thermocatalytic destruction, Photocatalytic reactions, Biological N_2_O reduction

## Abstract

Nitrous oxide (N_2_O) is the third-largest contributor to anthropogenic greenhouse gas forcing and the dominant anthropogenic ozone-depleting emission. While mitigation can flatten emissions, there is no clear pathway to eliminate residual emissions from agriculture and other diffuse sources, a key challenge for climate stabilization. The potential role of atmospheric removal has not been holistically assessed, yet N_2_O’s high potency and long atmospheric lifetime indicate the potential for a durable climate benefit. This review synthesizes emerging approaches for atmospheric N_2_O removal, organized by reaction mechanism (thermal destruction and thermocatalysis, photolysis, gas-phase radical reactions, photocatalysis, and biological reduction) and deployment format (closed versus open system). Each is evaluated for technical efficacy, climate viability, environmental and social viability, and economic plausibility using lower-bound cost models. No approach yet satisfies all four: gas-phase radical reactors are precluded by their kinetics, thermocatalytic and photocatalytic reactors require ten- to hundred-fold performance gains, and open-system approaches have the lowest cost floors but face the greatest measurement and governance challenges. These results identify the performance thresholds and broader constraints that govern feasibility across pathways, providing a foundation for a research agenda and for assessing the role of N_2_O removal alongside emissions mitigation.

## Graphical abstract

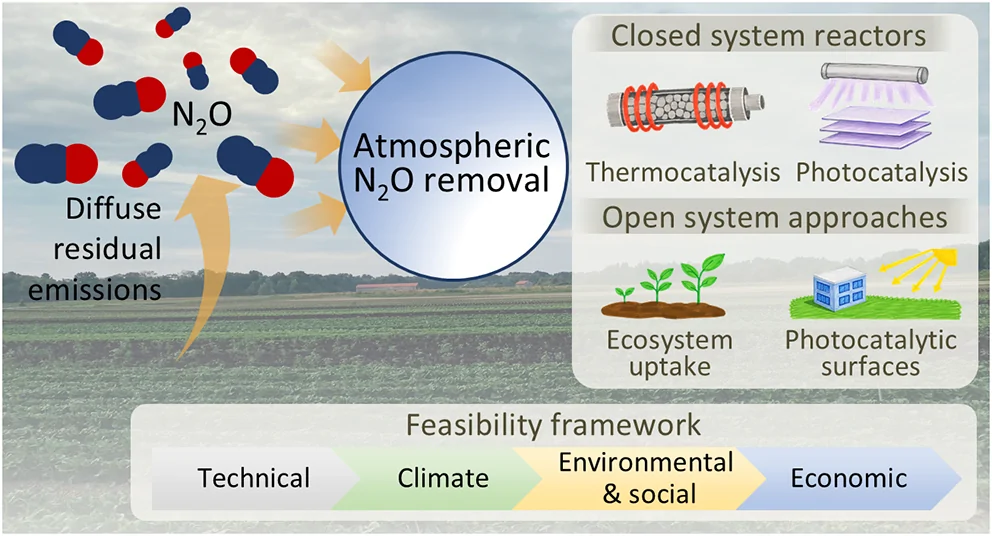

## 1. Introduction

Nitrous oxide (N_2_O) is the third-largest contributor to anthropogenic greenhouse gas forcing after carbon dioxide (CO_2_) and methane (CH_4_), contributing ∼0.1 °C of warming.^1^ Although far less abundant than CO_2_, reaching 339 ppb in 2025,^2^ its high radiative potency and ∼109-year lifetime are reflected in a global warming potential (GWP) of 273 on both 20- and 100-year time horizons.^1^ N_2_O produces a comparable global temperature response to that of CO_2_ for GWP-equivalent emissions (Figure 1), leading to climate impacts that persist well beyond a century.

**Figure 1:**
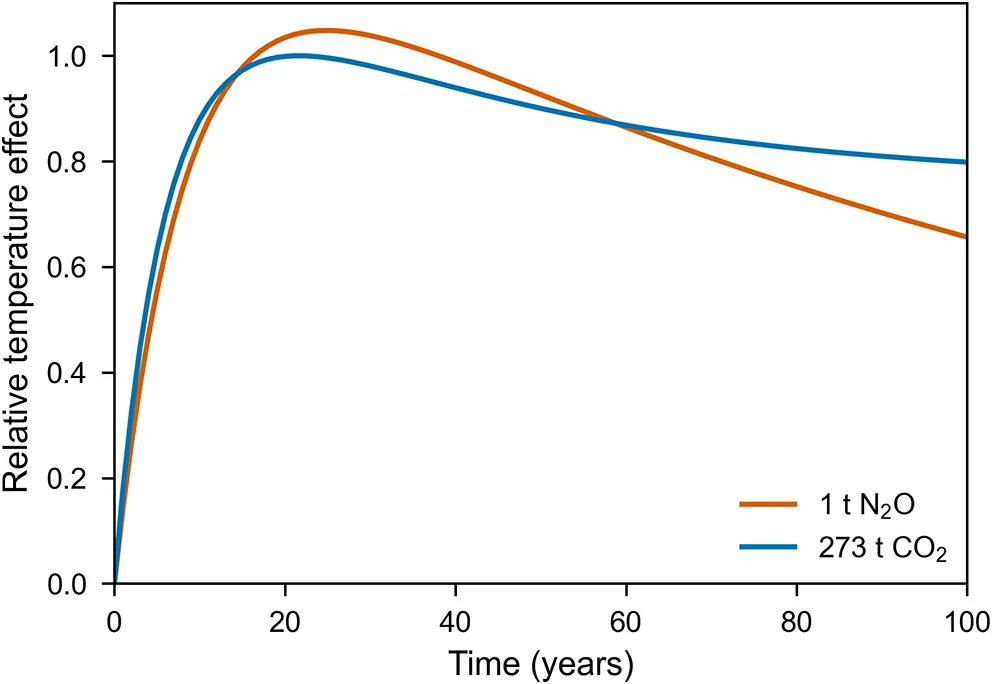
Temperature response to pulse emissions of nitrous oxide and a GWP-equivalent mass of carbon dioxide, normalized to the carbon dioxide peak; nitrous oxide produces a similar temperature response pattern (supporting calculations in SI Section S1.1).^3,4^

Consistent with this high radiative potency, the U.S. Environmental Protection Agency (EPA) estimates damages (social cost) of $67,000 t^–1^ of N_2_O, compared to $240 for CO_2_ and $2000 for CH_4_ (all costs herein are converted to 2025 United States dollars, see SI Section S1.2).^5^ Beyond climate, N_2_O is now the dominant anthropogenic ozone-depleting emission, equivalent to 15% of peak chlorofluorocarbon (CFC) emissions in the 1980s.^6^ This effect adds an estimated $2420 t^–1^ in human health and agricultural impacts.^7,8^ Steep CO_2_ and CH_4_ reductions could exacerbate this, as these gases indirectly increase stratospheric ozone, and unabated N_2_O emissions may then reverse the ozone hole’s recovery.^9^

Nearly two-thirds of direct anthropogenic N_2_O emissions arise from nitrogen fertilizers and manure applied in agriculture.^10^ While efforts focus on reducing nitrogen fertilizer demand and optimizing its management, there is no line of sight to reducing these emissions to zero.^9^ In contrast, concentrated sources, predominantly from adipic and nitric acid production, can be nearly eliminated using established thermal and catalytic abatement at estimated costs of approximately $6–23 tCO_2_e^–1^, yet comprise only ∼5% of anthropogenic emissions.^9,11^ The dominance of hard-to-mitigate agricultural emissions, together with the absence of any scalable natural sink, implies that stabilizing or reducing atmospheric N_2_O will be difficult through emissions mitigation alone.

### 1.1 Need for Removal Research

Climate pathways consistent with limiting warming to 2 °C require net-zero anthropogenic greenhouse gas emissions by 2100. These pathways retain substantial CH_4_ and N_2_O emissions and balance them with net-negative CO_2_ emissions.^12^ For N_2_O, they assume a ∼20% reduction in anthropogenic emissions over the coming decades, yet still require approximately 2 Gt CO_2_ y^–1^ of compensating carbon dioxide removal (CDR) by 2100.^12^ Closing the N_2_O contribution to net greenhouse gas emissions therefore rests on two levers, and both have a feasibility gap: widespread deployment of known N_2_O mitigation technologies appears sufficient only to flatten, rather than decrease, annual emissions,^9^ and large-scale CDR remains unproven and faces substantial scaling constraints.^13^

N_2_O removal is a novel approach to complement emissions mitigation by directly removing N_2_O from the atmosphere or decomposing it to N_2_ and O_2_. For residual agricultural emissions that are considered hard to abate, particularly diffuse emissions from fertilized cropland, grassland, and manure applied to soils, N_2_O removal could function as a like-for-like offset, ensuring that environmental effects, including ozone-related impacts, are more comparably offset than could be achieved with CDR. N_2_O destruction is particularly compelling for atmospheric greenhouse gas removal because it is climatically analogous to CDR (Figure 1) and avoids the storage and permanence challenges inherent to capture-and-storage-based CDR.

Completely removing the 0.339 ppm of N_2_O in a stream of ambient air is equivalent, on a molar GWP_100_ basis, to removing 92.5 ppm of CO_2_; the corresponding value for CH_4_ is 19.7 ppm of CO_2_ (Figure 2; SI Section S1.1). Although this comparison does not establish technical feasibility or cost, the relatively high GWP_100_-weighted removal value of N_2_O in ambient air makes it qualitatively appealing for further research relative to CH_4_ or halogenated compounds. N_2_O removal may also expand the total capacity for long-lived greenhouse gas removal if scaling constraints for N_2_O removal differ from those of CDR, or if the two can operate synergistically.

**Figure 2:**
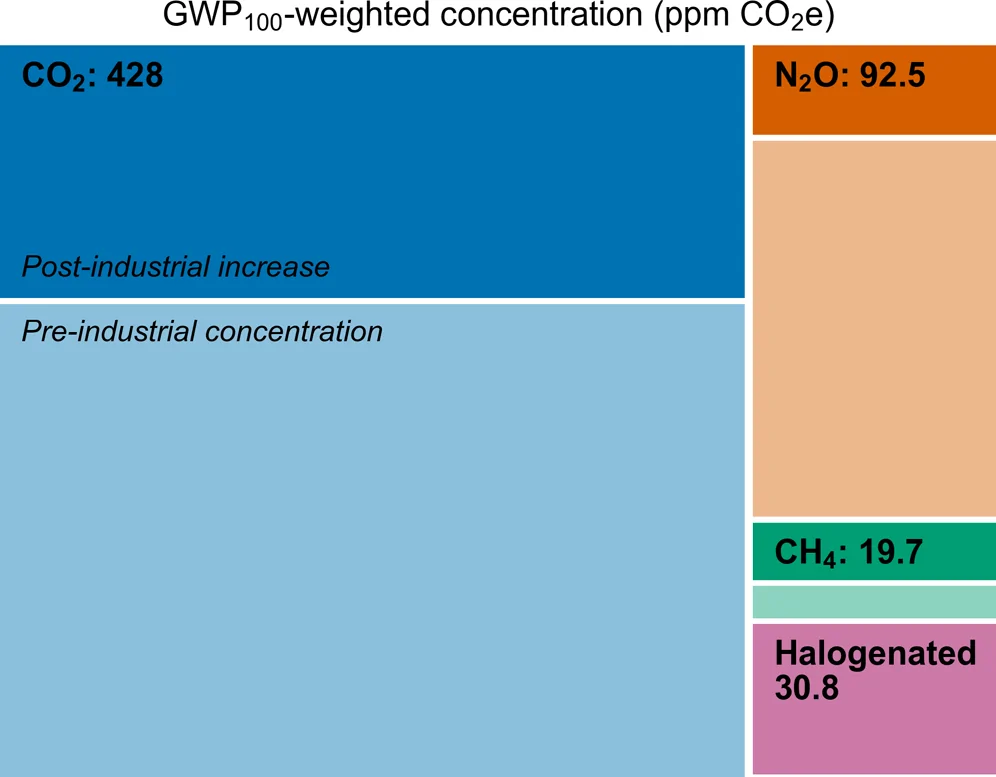
GWP_100_-weighted molar concentrations of the major greenhouse gases in ambient air, calculated as the atmospheric mole fraction of each gas multiplied by its molar GWP_100_ and expressed in ppm of CO_2_e (SI Section S1.1).^2,3,14–17^ For an atmospheric removal process, each value is the concentration of CO_2_ whose removal is equivalent, on a molar GWP_100_ basis, to complete removal of that gas from ambient air; this metric is not a measure of current radiative forcing. Preindustrial concentrations and postindustrial increases are shown by light and dark shading.

## 2. Overview of Removal Approaches

N_2_O removal could be accomplished by a variety of mechanisms, analogous to emerging CO_2_ and CH_4_ removal approaches, that exploit N_2_O’s distinctive chemical properties. Although decomposition of N_2_O to N_2_ and O_2_ is thermodynamically favorable, a high activation barrier suppresses spontaneous reaction under ambient conditions. Removal strategies therefore supply energy to surmount this barrier, lower it with a catalyst, or both. This assessment covers five N_2_O removal mechanisms: thermal destruction and thermocatalysis, photolysis, gas-phase radical destruction, photocatalysis, and biological reduction. These are deployed as either closed or open systems. A physical separation stage to concentrate N_2_O upstream of closed systems is assessed in Section 3.7.

Closed systems, analogous to CO_2_ direct air capture (DAC), are relatively self-contained industrial units which are actively driven by external energy inputs. Their scale is set by air throughput and residence time, and their cost by capital investment and energy consumption. Process control and monitoring of removal and environmental impacts are comparatively straightforward. In contrast, open systems operate in the ambient environment and rely on passive air contacting and natural energy inputs such as sunlight. Their scale and cost are determined by deployed surface area rather than airflow or energy consumption, with lower direct energy requirements but greater uncertainty in performance, monitoring, and environmental interactions.

Applying each mechanism in a closed or open system yields the matrix of approaches in Table 1. Each approach encompasses a family of physical, chemical, or biological processes that can be evaluated using a common analytical framework. This classification is not exhaustive or static: future work may identify additional mechanisms or approaches.

**Table 1:** A Framework for N_2_O Removal Approaches^a^

removal mechanism	closed-system approach	open-system approach
thermal and thermocatalysis	**thermal reactors** utilize high temperature (>700 °C) or medium temperature (200–400 °C) in combination with a catalyst to destroy N_2_O.^18,19^	
photolysis	**photolytic reactors** utilize UV–C photons which have sufficient energy to directly react with N_2_O.^20^	
gas-phase radical reaction	**radical reactors** utilize UV–C photons to generate O(^1^D) radicals from O_2_, which react with N_2_O.^21–23^	
photocatalysis	**photocatalytic reactors** utilize photocatalytic materials with artificial UV light sources. Photocatalyst materials drive chemical reactions with N_2_O when exposed to light.^24,25^	**photocatalytic surfaces** are coatings or powders deployed on built surfaces or open land, leveraging ambient sunlight and passive airflow.^26^ **Photocatalytic aerosols** are fine catalyst particles dispersed into the troposphere.^27^
biological reduction	**bioreactors** utilize microbial communities, organisms, or isolated enzymes to consume N_2_O via biological pathways.^28–30^	**ecosystem uptake enhancement** includes potential interventions that suppress N_2_O production or increase the population and productivity of N_2_O-consuming organisms in natural or managed ecosystems.^31–34^

a Representative citations identify mechanistic or experimental foundations for each populated approach.

### 2.1 Assessment Criteria

At present, it is not clear whether, or how, N_2_O removal could ultimately contribute to an overall climate response. Accordingly, this assessment evaluates idealized implementations to identify fundamental constraints, key uncertainties, and opportunities where targeted research and development could most effectively advance, or rule out, specific approaches. Three criteria, described below, are used to assess each approach.

#### 2.1.1 Net Mitigation Cost

Any approach must be net climate-beneficial, such that the cooling effect from N_2_O removal exceeds any warming caused by implementation. The net mitigation cost, in dollars per net metric ton of CO_2_ equivalent removed ($ tCO_2_e^–1^, using GWP_100_), is used as a single metric capturing both the efficacy of an approach and the cost of implementing it. No approach is developed enough for a full life-cycle assessment or a detailed techno-economic analysis, so only the primary climatic impacts (net N_2_O removal and CO_2_ emissions from energy use) and the major cost drivers are quantified; omitted impacts and costs would only raise the result, making these plausible lower bounds. Modeling methods and assumptions are described in the Supporting Information. The objective for this early stage assessment is to identify: (1) knowledge gaps in the net impact of the process, (2) whether fundamental energetic or material constraints are likely to preclude cost-effective deployment, and (3) whether research could plausibly improve performance to reach cost-competitiveness with other mitigation options.

#### 2.1.2 Environmental and Societal Considerations

In addition to climate impacts, N_2_O removal approaches present a range of potential benefits and risks for human health, ecosystems, and society, particularly for interventions deployed at large spatial scales. This criterion identifies which of those considerations are likely to be salient for a given approach. It does not evaluate whether an approach would ultimately be judged acceptable, a determination that requires a defined deployment context and stakeholders. Because a thorough life-cycle impact analysis is not possible at this technological maturity, the criterion instead identifies areas of greatest potential consequence or uncertainty, drawing on experience from analogous climate interventions such as CDR,^35,36^ atmospheric oxidation enhancement,^37^ and solar geoengineering.^38,39^ For open-system approaches in particular, large uncertainties about environmental and societal impacts may only be adequately addressed if considered in parallel with further technology development. The intent is not to exclude approaches a priori, but to highlight where nontechnical considerations are likely to be decisive, guiding research effort toward alleviating the most consequential barriers or reducing uncertainty.

#### 2.1.3 Scale

A common challenge for all greenhouse gas removal strategies is the large volume of air that must be processed, whether actively or passively. Closed-system approaches face scaling constraints similar to those of DAC: high capital intensity, dependence on low-emission energy, and the engineering constraints of large industrial infrastructure, compounded by the roughly 5-fold greater air volume required for the same gross removal (Figure 2). While open-system approaches may avoid some of these challenges, their scale is potentially limited by land use, fundamental constraints imposed by the Earth system, and social license to operate. Validation timelines may also be longer due to the need for field-scale experiments and the development of measurement, reporting, and verification (MRV). As a benchmark, a 2 °C scenario requires 2 Mt N_2_O y^–1^ (0.55 Gt CO_2_e y^–1^) removal by 2050 to close the gap between known and required mitigation and an additional 7.3 Mt N_2_O y^–1^ (2 Gt CO_2_e y^–1^) by 2100 to replace the CDR which implicitly offsets remaining N_2_O emissions.^12^ Scale is not itself a measure of merit for deployment: an otherwise favorable but site-limited intervention remains worth pursuing. It matters most for research prioritization, where a focus on scalable approaches could result in greater future removal.

### 2.2 Feasibility Framework

To integrate these criteria, a feasibility framework characterizes thresholds of evidence for climate-beneficial and cost-effective deployment of an N_2_O removal approach at scale. The framework (Table 2) consists of four successive levels that reflect increasing confidence in technical performance, climate benefit, and deployability.

**Table 2:** Levels of Deployment Feasibility

level	name	threshold of evidence
1	technical efficacy	the underlying chemical or biological mechanism can react with or remove N_2_O at atmospheric concentrations under environmentally relevant conditions.
2	climate viability	the approach has the potential to deliver net-negative CO_2_e emissions, based on experiments or validated models, when accounting for energy use and relevant greenhouse gas fluxes under realistic operating conditions.
3	environmental and social viability	the approach has manageable environmental side effects (with bounded uncertainty) and a credible pathway for MRV, regulation, and socially acceptable deployment at scale.
4	economic plausibility	high-fidelity, system-level cost analysis of a defined implementation yields estimated marginal costs below estimated marginal benefits, i.e., the social cost of N_2_O and comparable climate mitigation options.

Each level indicates necessary but not sufficient evidence and builds on the previous levels. Attaining the final level does not imply commercial readiness or recommend near-term deployment. Rather, it marks the point at which remaining uncertainties are sufficiently bounded to justify deployment-oriented research. A benchmark of $200 tCO_2_e^–1^ is used here, informed by the US EPA’s social cost of N_2_O at $250 tCO_2_e^–1^ (similar to CO_2_’s $240 tCO_2_e^–1^)^5^ and the $0.1–160 tCO_2_e^–1^ range of compliance market carbon prices.^40^ This framework is applied in Table 4 to summarize the current state of evidence across approaches.

## 3 Closed Systems

### 3.1 General Closed-System Cost Model

For a generic closed system, the net cost of removal, *C*_net_ ($ tCO_2_e^–1^), is modeled as

$$C_{net}=\frac{\frac{CA\mathrm{PE}X\cdot CRF}{V}+\mathrm{SEC}\cdot p_{el}}{M_{CO2e}-\mathrm{SEC}\cdot EF_{el}}$$ 1

This explicitly considers only two performance factors: the capacity-normalized capital cost of the system, CAPEX ($ cfm^–1^), and the specific energy consumption, SEC (Wh m^–3^ air). *p*_el_ ($ kWh^–1^) and EF_el_ (tCO_2_e kWh^–1^) are the cost and emissions factor for electricity. *M*_CO2e_ is the gross removal (tCO_2_e removed m^–3^ of air). CRF is the capital recovery factor for a project lifetime of *N* years, and *V* is the annual volume of air processed (m^3^ air cfm^–1^ y^–1^). Figure 3 shows *C*_net_ as a function of CAPEX and SEC, with a full derivation and assumptions in SI Section S2.

**Figure 3:**
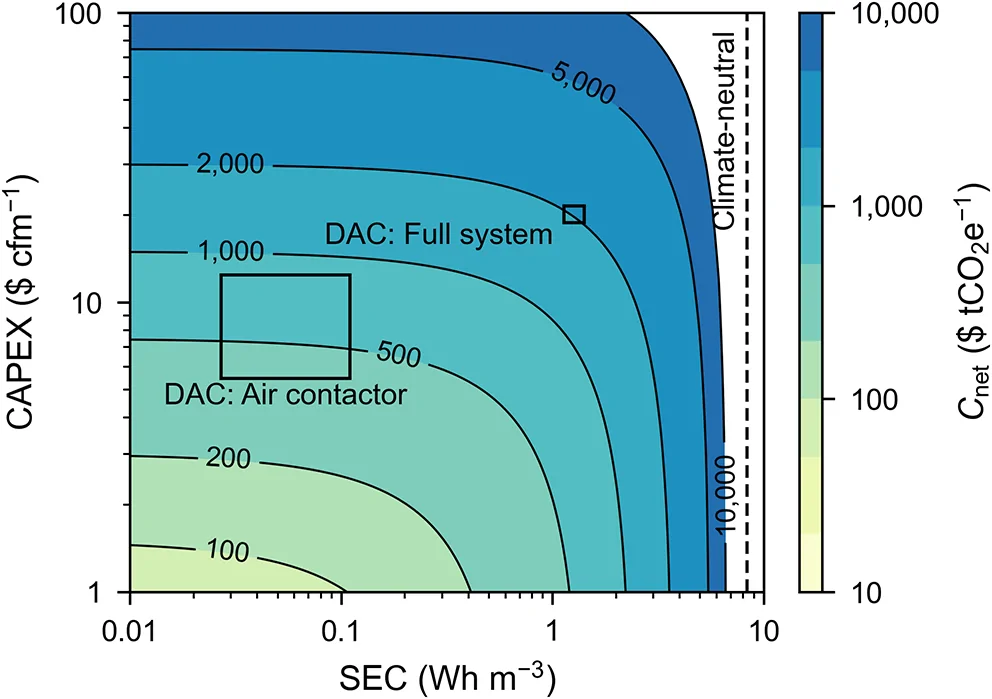
Net cost of atmospheric N_2_O removal (*C*_net_) in a generic closed system as a function of capacity-normalized capital cost (CAPEX) and specific energy consumption (SEC). Costs are reported per net metric ton of carbon dioxide equivalent (CO_2_e). The dashed line marks the climate-neutral threshold, at which emissions from electricity use equal the gross removal. Benchmark DAC costs^41^ and a full model description are reported in SI Section S2.

SEC provides a bridge between mechanism-level performance and preliminary cost estimation without assumptions about the energy source. Figures 4 and 6 therefore report SEC against mechanism-specific parameters, while Figure 3 maps SEC and CAPEX to net cost under the common assumptions above.

At low SEC, *C*_net_ is dominated by CAPEX; at higher SEC, by energy cost, with *C*_net_ diverging as SEC approaches the climate-neutral threshold of ∼8 Wh m^–3^, where SEC·EF_el_ = *M*_CO2e_. A removal process with similar CAPEX and SEC to those of the benchmark DAC plant^41^ is cost-prohibitive at >$1000 tCO_2_e^–1^.

### 3.2 Thermal and Thermocatalytic Reactors

#### 3.2.1 Mechanism

Thermal decomposition uses an elevated temperature to overcome the activation barrier, beginning at ∼700 °C with the rate increasing exponentially thereafter.^42^ Research has historically focused on pure N_2_O^43^ or industrial emissions destruction, where the heating energy is justified by the high concentration.^18^ Reducing this heating energy is key for making thermal destruction viable at lower concentrations.

One approach is to incorporate a heat recovery system that preheats the incoming air with waste heat from the treated exhaust, similar to commercially available regenerative thermal oxidizers (RTOs). RTOs have a heat-exchanger efficiency (ε_HX_) of 90–97%, reducing the amount of external heat needed by 10- to 33-fold, respectively.^44–47^ The second approach is to use a thermal catalyst, which lowers the required temperature. A wide range of materials, from noble metal nanoparticles to transition metal oxides and metal-doped zeolites, have been studied.^19^ The *T*_50_, the temperature at which 50% of N_2_O is decomposed, is commonly reported, but it also depends on gas flow rate, catalyst mass, and N_2_O concentration, and so does not support comparison across studies. Many materials have demonstrated *T*_50_ < 300 °C, and the highest-performing material, cobalt spinel (Co_3_O_4_) doped with other metals, shows *T*_50_ < 200 °C, resulting in a 3- to 4-fold reduction in heating energy compared to thermal destruction.^19,48^

More experimental characterization of these catalysts is needed at atmospheric concentrations. Typical experimental conditions are 1000 ppm to 5% N_2_O in a dry inert carrier gas, although O_2_ and H_2_O inhibit decomposition, increasing *T*_50_ by ∼100 °C for some catalysts.^49^ Yet, some of the best performance was demonstrated in a study of Zn-doped Co_3_O_4_ at 1000 ppm, with *T*_50_ ≈ 150 °C in a helium environment and ∼250 °C with 10% O_2_ and 5% H_2_O, indicating the potential for improved performance at lower N_2_O concentration.^49^ The literature on noncatalytic thermal decomposition is similarly focused on high-concentration applications, although several studies have been conducted down to 400 ppm.^50,51^

#### 3.2.2 Cost Drivers and Performance Targets

The state-of-the-art solution for thermal destruction of industrial emissions is the noncatalytic RTO, with total capital cost equivalent to CAPEX = $44–58 cfm^–1^ (SI Section S3).^52^ These systems use ceramic media for thermal storage and could accommodate catalyst monoliths in the intervening combustion chamber. The material cost for the highest-performing catalyst above, Zn-doped Co_3_O_4_, is estimated at ∼$3 cfm^–1^ (SI Section S3), and reduced *T*_rxn_ may enable lower hardware costs.^52^ Heating energy is calculated as a function of *T*_rxn_ and ε_HX_ (Figure 4, SI Section S3). An RTO with *T*_rxn_ = 200 °C (near the best reported catalytic *T*_50_ values) results in SEC = 1.75 Wh m^–3^ and *C*_net_ ≈ $4400 tCO_2_e^–1^. Theoretically, the use of waste heat could reduce *C*_net_ to < $200 tCO_2_e^–1^, yet colocation with high-temperature waste heat sharply constrains the ultimate scalability (Section 3.8).

**Figure 4:**
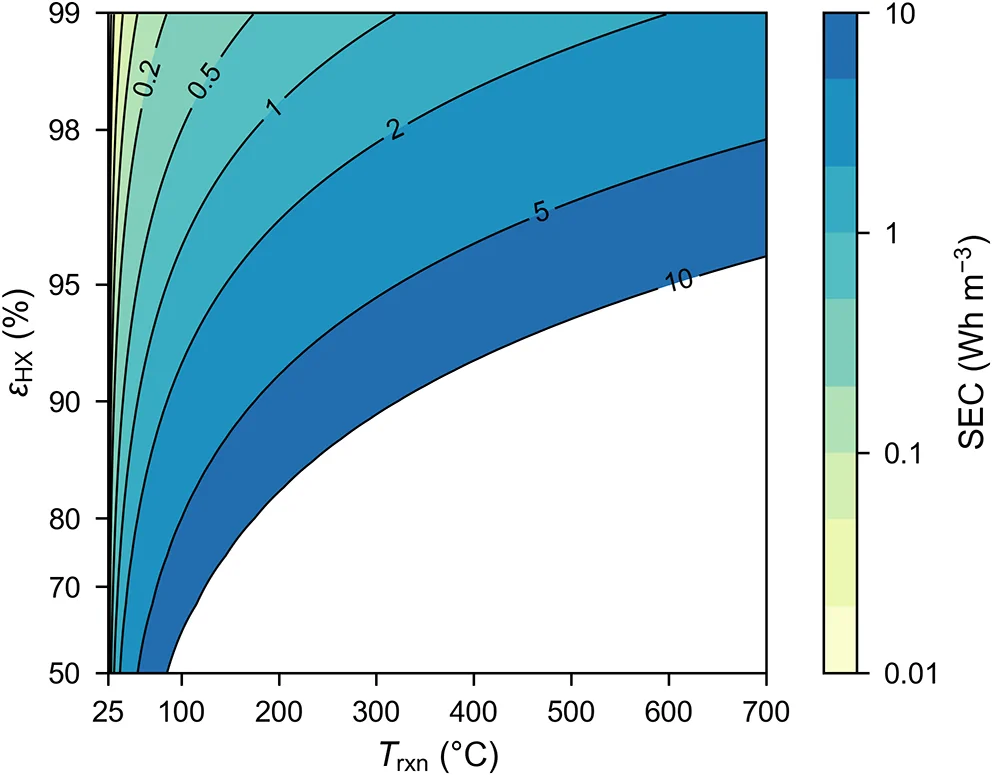
Heating energy requirement, expressed as specific energy consumption (SEC), for thermal N_2_O removal as a function of reaction temperature (*T*_rxn_) and heat-exchanger efficiency (ε_HX_).

While current standalone thermocatalytic systems could not achieve cost-effective operation, two research priorities remain valuable: systematic characterization of existing catalysts under atmospheric conditions, including kinetics, inhibition mechanisms, and long-term durability; and detailed assessment of RTO capital-cost drivers for a low-temperature system. Establishing these baselines will help scope the technical challenge of cost-effective thermal destruction, whether through novel manufacturing processes, heat recovery architectures, or alternative catalytic pathways.

### 3.3 Photolytic Reactors

#### 3.3.1 Mechanism

Photolysis is a reaction in which an N_2_O molecule is broken apart by the energy delivered by a photon of light. For this to occur, the photon must have sufficient energy to break the N–O bond and a wavelength that is absorbed by N_2_O. N_2_O absorbs a small fraction of light in the UV range below 240 nm (Figure 5a) and in the infrared, which causes its radiative forcing effect.^53^ Experimentally, 140–230 nm photons split N_2_O into N_2_ and a lone O atom with a quantum yield (the number of molecules of N_2_O decomposed per photon) of one.^20^ Yet, light in these wavelengths is also absorbed by ozone (O_3_) and O_2_, thus the apparent quantum yield (AQY_N2O_), incorporating the fraction of photons that reach an N_2_O molecule, is much lower (Figure 5b). Photolysis in the stratosphere accounts for about 90% of the natural N_2_O sink, but does not occur in the troposphere, where the upper atmosphere has absorbed all <240 nm photons.^21^

**Figure 5:**
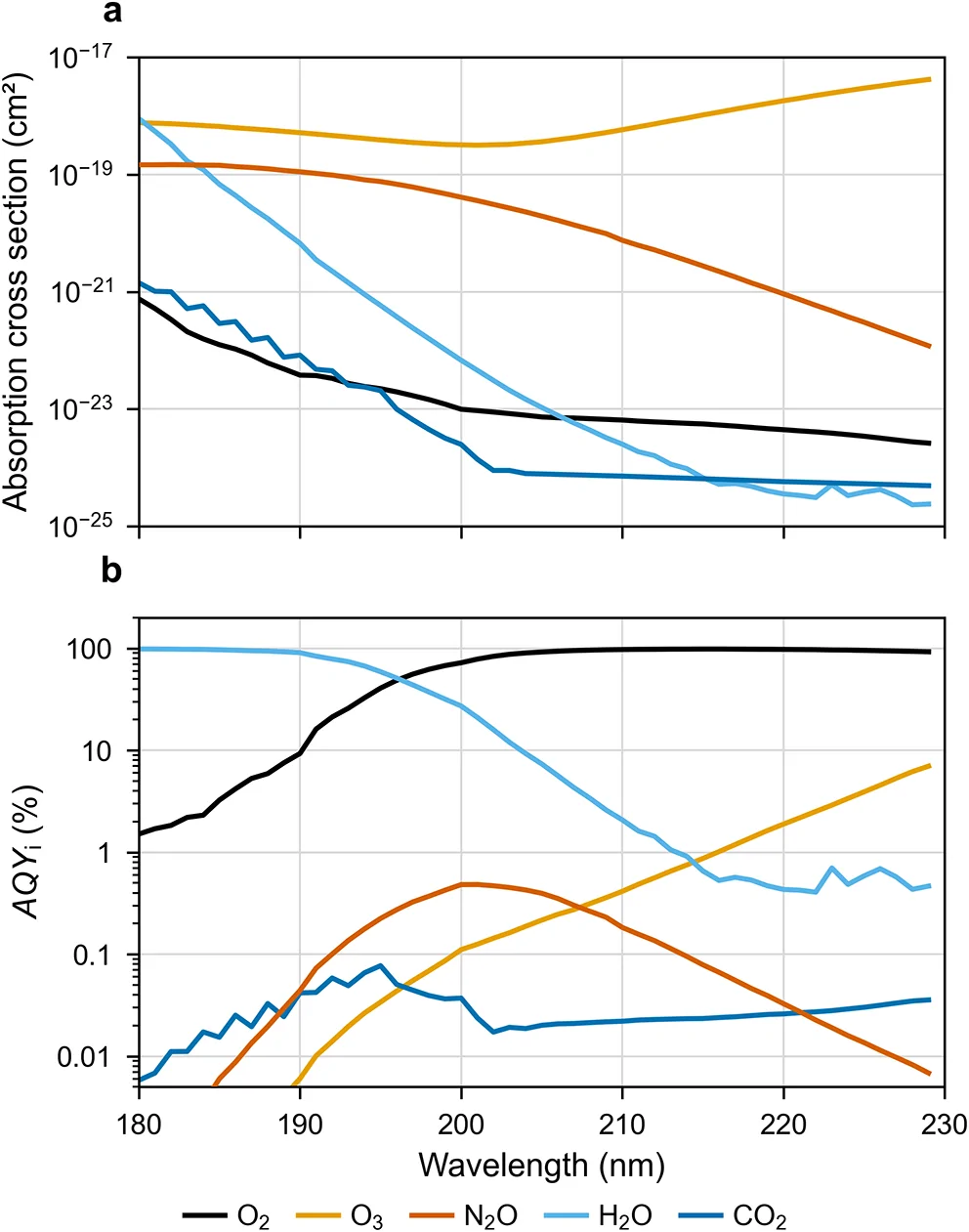
(a) Absorption cross sections for N_2_O and other UV-active atmospheric constituents.^20,54–56^ (b) Apparent quantum yield (AQY_*i*_), which accounts for the fraction of incident photons absorbed by each species rather than competing species, for tropospheric conditions of 1.1 mol % water vapor (∼37% relative humidity at 25 °C) and 10 ppb O_3_. Model details are reported in SI Section S5.

#### 3.3.2 Cost Drivers and Performance Targets

Under typical tropospheric conditions and assuming all emitted photons are absorbed by gases rather than lost at the reactor surfaces, AQY_N2O_ reaches a maximum of ∼0.5% at 201 nm (Figure 5b). The total extinction length is 159 m at 201 nm, thus achieving high photon utilization requires large-volume reactor designs, which incidentally allow for unobstructed gas flow and a low air-handling pressure drop (SI Section S5). An idealized 201 nm source converting all electrical input to photons would give SEC ≈ 0.5 Wh m^–3^ and an electricity cost of ∼$150 tCO_2_e^–1^. Costs in this section are reported as electricity cost alone, because no capital cost basis is available for light sources at these wavelengths.

Two limits reduce this idealized performance in practice. First, most photons are absorbed by O_2_ and H_2_O rather than N_2_O, producing O­(^1^D), O­(^3^P), and OH. Although O­(^1^D) can react with N_2_O (Section 3.4), the majority instead form O_3_, so indirect destruction is negligible and accumulating O_3_ further suppresses AQY_N2O_ as the reactor operates. Second, few sources emit near 201 nm. KrBr* (207 nm) excimer lamps are the closest available, but the offset wavelength lowers AQY_N2O_ to 0.3% and their radiant efficiency is only η_rad_ = 5–18% (SI Section S5), moving the SEC floor to ∼ 4 Wh m^–3^ (Figure 6).

**Figure 6:**
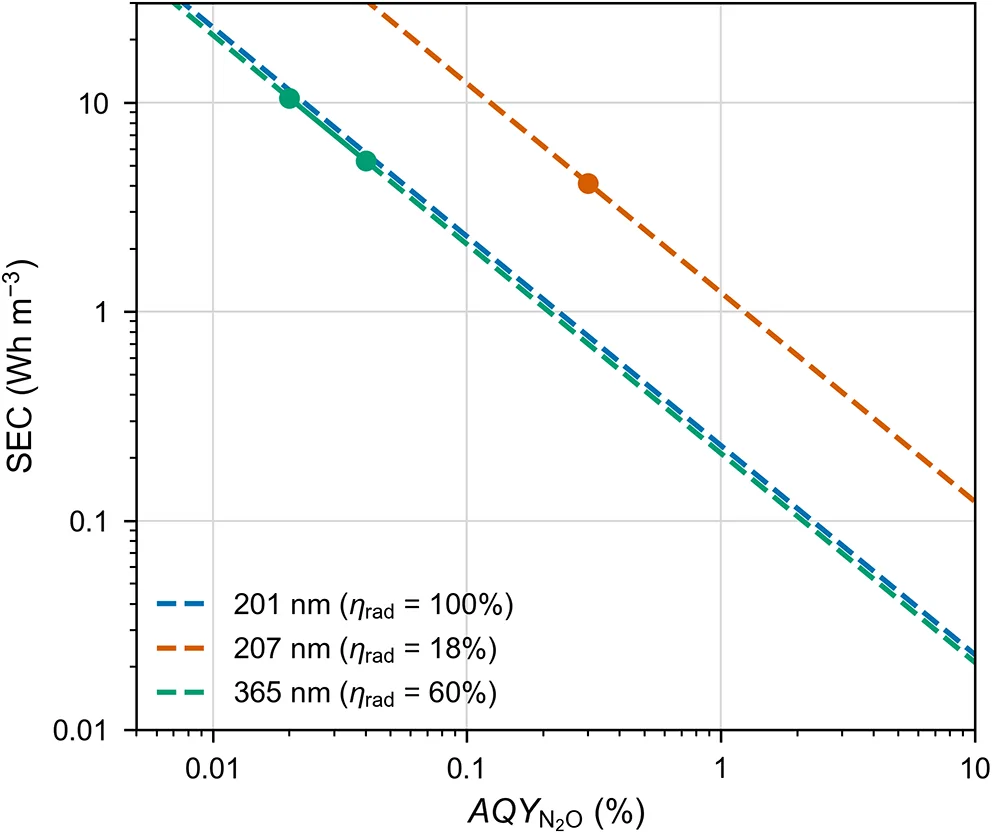
Specific energy consumption (SEC) of photolytic and photocatalytic reactors as a function of their respective apparent quantum yield for N_2_O destruction (AQY_N2O_). Radiant efficiency (η_rad_) specifies the fraction of electrical input converted to useful optical output.

While these limits highlight the enabling role of advanced light source development for reducing SEC to a practical level, a deeper understanding of the secondary chemical reactions is also needed to assess potential harms and cobenefits. OH radicals may provide additional air quality and climate benefits via oxidation of ambient CH_4_, NO_*x*_, and other pollutants,^57^ while O_3_ is both a harmful air pollutant at ground level and a greenhouse gas.^58^

### 3.4 Gas-Phase Radical Reactors

Reactions with energetic oxygen radicals, O­(^1^D), in the stratosphere comprise the remaining 10% of the natural N_2_O sink, after photolysis.^21^ O­(^1^D) is formed by O_3_ photolysis with UV light.^22^ Upon collision with N_2_O, O­(^1^D) initiates a reaction, forming either N_2_ and O_2_ or 2 NO. (The resulting NO can later react with O_3_, the mechanism by which N_2_O depletes the ozone layer.) While the quantum yield of O­(^1^D) production from O_3_ and the O­(^1^D) reaction with N_2_O are each approximately one, most O­(^1^D) is instead deactivated by collision with N_2_ or O_2_,^23^ so the AQY_N2O_ of the entire pathway is negligible (SI Section S5). To my knowledge, there have been no efforts to enhance this pathway or replicate it in an engineered system. Beyond O­(^1^D), N_2_O is relatively unreactive with other natural atmospheric radicals. The N_2_O reaction rates with OH and Cl radicals, the primary atmospheric sink for CH_4_ and other organic compounds, are 6 orders of magnitude slower than that with O­(^1^D).^20,59^

### 3.5 Photocatalytic Reactors

#### 3.5.1 Mechanism

Photocatalysts catalyze a chemical reaction when a UV or visible photon is absorbed, exciting an electron which can then react with nearby molecules, including N_2_O.^24^ AQY_N2O_ is the number of N_2_O molecules decomposed per photon delivered to the system. This metric is set by intrinsic material properties, chiefly bandgap, surface adsorption of N_2_O, and charge recombination, and by extrinsic conditions including the photon’s wavelength, the concentration of N_2_O, and competing species (SI Section S6). Traditional photocatalysts such as titanium dioxide (TiO_2_) absorb only in the UV,^60^ where little of the solar spectrum lies^61^ and artificial light is more expensive and less efficient.^62^ At low N_2_O concentrations, AQY_N2O_ is expected to decrease as fractional N_2_O surface coverage falls; however, this relationship has not been established experimentally and may additionally depend on humidity, irradiance, and competing species.^63^ The majority of N_2_O photocatalysis literature is at higher concentrations, often in an inert gas instead of air, and rarely with environmentally relevant humidity. Thus, these studies are not directly applicable to understanding the potential AQY_N2O_ here. Sano et al.’s work showing AQY_N2O_ ≈ 9% at 1000 ppm^25^ has been referenced to indicate the high potential of photocatalytic decomposition in a climate context,^27,64^ yet their experiments used methanol vapor as a reductant and showed no measurable activity in the absence of methanol.

Several photocatalytic removal approaches have been described, yet none, to my knowledge, report AQY_N2O_ at relevant conditions. Two research groups testing photocatalytic pollution mitigation in barns have shown a small degree of incidental N_2_O removal at near-ambient concentration using TiO_2_-based commercial coatings and artificial light.^65,66^ Yet, this effect was not replicated in a follow-up study, and none report sufficient details to estimate AQY_N2O_.^67^ A single outdoor study reported atmospheric removal using a TiO_2_-based catalyst, corresponding to AQY_N2O_ = 0.02–0.04% (Section 4.2).^26^

#### 3.5.2 Cost Drivers and Performance Targets

Experimental work typically uses relatively efficient UV-A LEDs, e.g., η_rad_ = 60% at 365 nm.^68^ Even so, a potential photocatalyst with AQY_N2O_ = 0.02–0.04% results in a lighting SEC of 5–11 Wh m^–3^ (Figure 6) and an estimated CAPEX for the LED modules alone of $30–60 cfm^–1^ (SI Section S6), well above the benchmark air contactor (Figure 3) on both metrics, while the TiO_2_-based catalyst cost is negligible by comparison.^27^

At the lower end of this range the process is not climate beneficial on a net basis. Achieving *C*_net_ < $200 tCO_2_e^–1^ would require AQY_N2O_ = 0.7%, an improvement of more than an order of magnitude that exceeds other dilute photocatalytic processes, discussed further in Section 4.2. Yet, the lack of baseline performance data under atmospheric conditions and the potential for large cost reductions if AQY_N2O_ can be improved motivate further baseline characterization of existing materials and new research efforts into higher-performance photocatalysts.

### 3.6 Bioreactors

#### 3.6.1 Mechanism

The reduction of N_2_O to N_2_ occurs in biological systems as the final step of denitrification, part of the biological nitrogen cycle. The N_2_O reductase enzyme (N_2_OR)^69^ is the only known enzyme to catalyze this reaction^70^ (SI Section S10). N_2_O-reducing organisms and, in theory, isolated N_2_OR can be hosted in bioreactors, engineered systems that provide a controlled environment to support their activity. While several systems have been studied to treat high-concentration (>100 ppm) emissions, no reports focusing on atmospheric removal were found.

The challenge of operating at ambient concentration is especially acute for biological processes because gaseous N_2_O must first dissolve into an aqueous phase to reach an enzyme’s active site, introducing additional mass transfer steps which reduce the overall reaction rate. Because N_2_O is more soluble, this barrier is less significant than for CH_4_, which has seen far more interest in low-level and atmospheric removal bioreactors.^71–74^ Accounting for their atmospheric concentrations, the aqueous equilibrium concentration of N_2_O is 3 times that of CH_4_ (8.20 versus 2.74 nM), or 81 times that of CH_4_ on a CO_2_e basis (see SI Section S7). Yet, an additional challenge for N_2_O removal is that N_2_OR requires an anoxic environment,^28,29^ with oxygenated conditions at the enzyme site consistently showing orders-of-magnitude decreases in activity.^75,76^

#### 3.6.2 Cost Drivers and Performance Targets

In contrast to the other closed-system approaches, bioreactors are indicated to have relatively low SEC, just that required for handling air and other inputs, so *C*_net_ is dominated by CAPEX, which in turn depends on the specific design of the reactor and the process intensity. There are two main strategies to create the necessary anoxic environment: bulk O_2_ removal using a multistep process or the creation of localized anoxic microsites within the biological media. For atmospheric N_2_O removal, a key distinction between these strategies is the amount of O_2_ consumed through oxidation of organic carbon, which produces CO_2_.

One demonstration of N_2_O removal with minimal CO_2_ production is a pilot study of a biofilter to treat swine barn pit ventilation exhaust.^30^ This system achieved 14–18% removal at 0.43–0.56 ppm of N_2_O with no indication of significant O_2_ depletion in the exhaust, implying the efficient creation of locally anoxic microsites.^30^ The underlying biofilter used a 7.6 s empty-bed residence time with an average fan pressure drop of 125 Pa, and is modeled with CAPEX = $10.56 cfm^–1^ and SEC = 0.28 Wh m^–3^, resulting in *C*_net_ ≈ $800 tCO_2_e^–1^ (SI Section S8). Characterization of the bacterial community here and in similar biofilters may yield insights on aerobic N_2_O respiration applicable to other architectures and indicate the potential to increase removal efficiency in this context.

In contrast, multistage systems have been developed to mitigate higher-concentration N_2_O emissions (∼100 ppm) from wastewater treatment plants.^77–79^ These systems use the oxidation of organic-rich wastewater to deplete O_2_ from the gas stream in an initial stage, enabling greater N_2_O reduction under less-oxic conditions in a subsequent stage. Extending this approach to atmospheric removal is challenged by the CO_2_ emissions associated with O_2_ depletion: at atmospheric N_2_O concentrations, depleting just 10% of the O_2_ entering the air contactor—a level observed in laboratory tests^78^—produces CO_2_ emissions 226 times greater than the N_2_O removed on a CO_2_e basis. Bulk O_2_ removal therefore cannot deliver a net climate benefit at atmospheric concentrations, and reducing the required O_2_ depletion far enough to permit one converges on the localized-microsite strategy described above.

Achieving cost-effective removal will require further reductions in CAPEX, constraining solutions to simple biofilter-like designs. Under this constraint, the boundary between a large-scale closed-system reactor and a highly managed ecosystem becomes less distinct. Designs that explicitly span this continuum are under-explored and may best balance cost and removal performance.

### 3.7 Concentrators

For the mechanisms considered here, both CAPEX and SEC scale with the volume of air handled rather than with the N_2_O concentration, so enriching the feed with an upstream concentrator lowers the cost of destruction by approximately the same factor. To reduce *C*_net_, the concentrator must therefore have lower CAPEX and SEC than the destruction process. While no such device has been proposed for N_2_O, existing air contactors already exceed the $3 cfm^–1^ CAPEX ceiling consistent with removal at $200 tCO_2_e^–1^ (Figure 3, SI Section S2). Further, the carbamate- and carbonate-looping chemistries of the two best-developed DAC processes^80^ have no analog for N_2_O, which is not acidic and forms no comparable stable adduct.

Any pressure-driven route is further constrained by the energy budget: even at the climate-neutral threshold of 8 Wh m^–3^, reversible isothermal compression permits a pressure ratio of only 1.33, whereas demonstrated membrane and swing-adsorption processes operate at ratios of 4–15.^81,82^ Reported membrane selectivity of 5–10 against O_2_ and N_2_ could raise ambient N_2_O only to a few ppm in a single-stage process,^83,84^ 2 orders of magnitude below the 300–3500 ppm of N_2_O of nitric acid tail gas, for which existing sorbents are already reported insufficient.^85^

Physisorption presents a more equivocal case. Because N_2_O shares many chemical properties with CO_2_, sorbents take up the two almost equally,^86,87^ and N_2_O uptake is suppressed by competing water adsorption much as CO_2_ uptake is in DAC.^88,89^ Co-enrichment of CO_2_ is not inherently an obstacle, but with CO_2_ present at 1263 times the N_2_O concentration, sorbent volume, contacting area, and regeneration energy would be orders of magnitude higher than a more selective process. However, if integrated with a CO_2_ DAC plant, coenrichment at a comparable efficiency could deliver N_2_O at ∼750 ppm, sufficient for existing thermocatalytic abatement,^11,19^ increasing gross CO_2_e removal by 22%. In some cases, N_2_O chemisorption proceeds by dissociation: at metal sites in exchanged zeolites, half or more of the adsorbed N_2_O is converted to N_2_ and O_2_ rather than recovered on regeneration.^88,90^ A sorbent that both took up N_2_O and decomposed it would need no second treatment stage.

The relative uptake of N_2_O and CO_2_, and the potential for in situ decomposition, depend strongly on the specific sorbent chemistry. N_2_O is not typically measured in studies of DAC sorbents or in surveys of captured CO_2_ stream impurities,^91^ but this characterization could be done at laboratory and facility scale.

### 3.8 Scale and Deployment Considerations

The closed-system format offers two deployment advantages: no hard limit on how much capacity can be built, and containment that limits the magnitude of undesired environmental impacts relative to open systems. Scale is therefore governed almost entirely by efficacy and cost, yet most closed-system approaches remain far from the $200 tCO_2_e^–1^ benchmark, requiring improved energy efficiency, low-emissions-intensity energy, and lower capital intensity.

Systems could be standalone, analogous to DAC plants, or colocated with existing industrial infrastructure to use waste streams such as low-grade heat or organic matter, or to share the capital cost of air handling. Colocation may reduce *C*_net_ in the right setting, but the scale of such opportunities is limited: for example, the most optimistic use of waste heat in the EU, without heat recovery, would power only ∼1.7 Mt CO_2_e y^–1^ of thermocatalytic N_2_O removal (SI Section S4). Whether colocated or standalone, closed-system approaches will likely resemble familiar industrial infrastructure in form and function, an indicator that their impacts and oversight could be addressed within existing regulatory and social frameworks. Yet, as seen with DAC and point-source CO_2_ capture projects, the siting of large industrial climate mitigation facilities can raise environmental justice concerns around localized environmental and social impacts.^92,93^ While N_2_O removal systems may share similar local impacts with respect to energy or resource use, there is a potential for many approaches to improve local air quality through the incidental codestruction of harmful air pollutants including VOCs, ammonia, NO_*x*_, CH_4_, and odor-causing compounds. Yet, any unacceptable byproducts must be prevented at the source because additional treatment steps carry high marginal cost and an energy penalty.

## 4. Open Systems

Because open-system approaches rely on passive air contact and ambient energy,^37^ their performance is governed primarily by the cost and removal rate per unit area, with negligible direct energy input. Thus, this analysis focuses on achievable areal removal rates, associated costs, and potential environmental interactions. Only mechanisms that can operate effectively using ambient environmental energy are compatible with an open-system approach. Known thermocatalysts require temperatures significantly above ambient conditions, necessitating an energy source for heating. Likewise, photolysis at ground level requires artificial UV light generation, as this portion of the solar spectrum is attenuated in the upper atmosphere.

### 4.1 General Open-System Cost Model

The gross cost of removal for a generic open-system approach, *C*_open_ ($ tCO_2_e^–1^), is modeled as

2 $$C_{open}=\frac{C_{A}}{R_{\mathrm{CO}2e}}$$

This explicitly includes only two factors: the annualized intervention cost, *C*_A_ ($ ha^–1^ y^–1^), and the annual average removal rate, *R*_CO2e_ (tCO_2_e ha^–1^ y^–1^). If impacts on emissions beyond N_2_O are known, e.g., concomitant change in natural CO_2_ or CH_4_ fluxes or energy use for deployment, *R*_CO2e_ can be treated as a net removal rate, and *C*_open_ as a net cost (full derivation reported in SI Section S9.1). For open systems, *R*_CO2e_ serves the same role as SEC does for closed systems, separating mechanistic performance from the intervention-cost scenarios illustrated in Figure 7.

**Figure 7:**
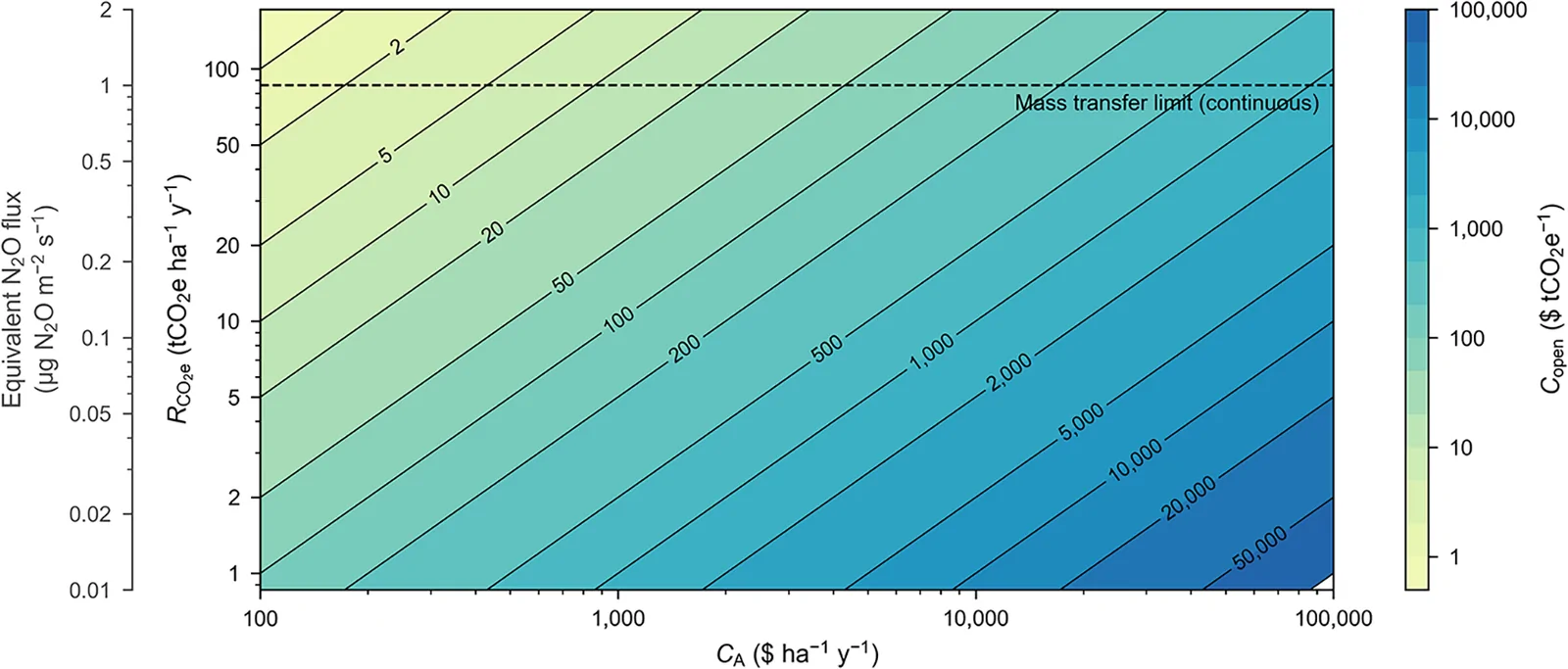
Estimated cost of atmospheric N_2_O removal in an open system (*C*_open_) as a function of annualized intervention cost per area (*C*_A_) and annual average CO_2_-equivalent removal rate per area (*R*_CO2e_). The dashed line marks the surface–atmosphere mass-transfer limit for continuous operation. A full description of the model is reported in SI Section S9.

Figure 7 illustrates the resulting cost as a function of removal rate and intervention cost. Atmosphere–surface mass transfer limits the instantaneous removal flux to <1 μg N_2_O m^–2^ s^–1^ under typical outdoor conditions, equivalent to ∼90 tCO_2_e ha^–1^ y^–1^ if sustained continuously (dashed line in Figure 7; SI Section S9.2). For photocatalytic systems, the full-sun AQY_N2O_ is converted to an annual-average removal rate using a solar capacity factor, here a representative 22.9% (SI Section S9.3).

### 4.2 Photocatalytic Surfaces

#### 4.2.1 Mechanism

Photocatalytic N_2_O destruction is governed by the same surface photochemistry described for photocatalytic reactors (Section 3.5.1). While most photocatalysts require UV photons, incident sunlight is predominantly in the visible range, with only a few percent of total irradiance in the UV band. Thus, photocatalyst performance is determined by AQY_N2O_ within the UV band and by the cutoff wavelength set by the catalyst’s bandgap, which fixes the usable irradiance. Extending the cutoff from 365 nm to 395 or 427 nm raises the usable photon flux two- or 4-fold (SI Section S9.3).

As an initial benchmark, the only experimental evidence identified for open-system atmospheric N_2_O removal was a TiO_2_-based field study; while AQY_N2O_ was not reported directly, a range of 0.02–0.04% was calculated from published data (SI Section S6).^26^ TiO_2_ coatings have been studied for NO_*x*_ and VOC removal on street surfaces,^94^ highway sound barriers,^95^ glass surfaces,^96^ and building exteriors,^64^ and for CH_4_ oxidation in laboratory settings.^97^ Reported NO destruction of 0.1–0.3 μg m^–2^ s^–1^ at 2000 ppb^64^ and NO_*x*_ destruction with AQY = 0.05–0.1% at <100 ppb^96^ demonstrate activity under ambient conditions, including in long-duration studies subject to weathering and surface fouling.^64,94^

NO_*x*_ and CH_4_ removal follow distinct photocatalytic pathways from N_2_O decomposition (oxidation versus reduction, SI Section S6) so their reported AQYs should be viewed as contextual benchmarks rather than quantitative predictors of AQY_N2O_. Recent controlled measurements of CH_4_ oxidation across 2–5000 ppm and varying humidity provide a useful template for the concentration- and condition-resolved measurements now needed for N_2_O.^97^ Rigorous measurements of AQY_N2O_ across relevant concentration, humidity, irradiance, and copollutant conditions, with product identification, are therefore a high-leverage near-term research priority.

#### 4.2.2 Cost Drivers and Performance Targets

Most outdoor photocatalyst work has used durable coatings on static surfaces. Coating costs are highly sensitive to location, labor, and maintenance, here estimated as *C*_A_ = $8400–30,700 ha^–1^ y^–1^ based on commercial roof cost reports of $3.8 m^–2^ (India) to $18.8 m^–2^ (United States) for initial coating and $0.27 m^–2^ y^–1^ for average annual maintenance (SI Section S9.5).^27,98,99^ At the upper-estimate AQY_N2O_ of 0.04%, for a coating whose area is entirely active catalyst (SI Section S9.5), this range corresponds to *C*_open_ ≈ $380–1400 tCO_2_e^–1^. Achieving *C*_open_ below the $200 benchmark likely requires a combination of improved AQY_N2O_ and low labor cost or labor-saving innovations in coating and maintenance. Increasing AQY_N2O_ alone is unlikely to provide a path to *C*_open_ < $200 tCO_2_e^–1^ except at the lower end of the *C*_A_ range. Mobile surfaces such as vehicle exteriors and solar updraft towers that passively drive airflow have both been proposed to achieve higher *R*_CO2e_.^100,101^ Yet the only field-measured flux, from a field 10–20% covered by catalyst (below), is an order of magnitude below the mass-transfer limit. A coating whose area was entirely active catalyst would be within a factor of 2 of that limit, so added airflow would help only if AQY_N2O_ also improved (SI Section S9.4).

An alternative format is powder dispersal using methods from the agricultural industry (e.g., aerial application or tractor-based boom sprayers). To my knowledge, the only field tests of open-system N_2_O removal in this manner used a proprietary TiO_2_-based catalyst.^26^ While the deployment cost is low (e.g., $20 ha^–1^ for existing agricultural powder dispersal),^102^ the active lifetime may be only days to weeks,^103^ requiring multiple annual applications (increasing *C*_A_), and surface coverage is incomplete, reducing *R*_CO2e_. This 40-ha field trial reported a 0.1 μg m^–2^ s^–1^ change in N_2_O flux from a control field,^26^ yet this difference is small relative to the variability between samples and between visits, and the experiment lacks a robust baseline (SI Section S6). Annualized over a 90-day growing season in full-sun-equivalent hours (SI Section S6), this corresponds to a removal of 0.5–2 tCO_2_e ha^–1^ and requires 12 applications, totaling 3 kg ha^–1^. The dispersal cost is *C*_A_ = $240 ha^–1^ y^–1^ (12 dedicated spray applications), resulting in *C*_open_ ≈ $120–480 tCO_2_e^–1^ (Figure 7). Yet, higher-fidelity N_2_O flux measurements and a more robust experimental design are needed to establish whether removal occurs and to constrain AQY_N2O_. Additional measurements of CH_4_ and CO_2_ fluxes are needed to evaluate the net greenhouse gas removal rate, while characterization of the complete nitrogen cycle and crop yields is needed to attribute the change in N_2_O flux to the photocatalytic removal mechanism.

Finally, fine catalyst powders could instead be dispersed as aerosols into the atmosphere. Randall et al. modeled tropospheric, i.e., low-altitude, dispersal, only accounting for catalyst activity while aloft.^27^ They estimate a dispersal cost of $0.80 kg^–1^, 2 orders of magnitude below the estimate here for field spraying ($80 kg^–1^ for *C*_A_ = $240 ha^–1^ y^–1^ and 3 kg_cat_ applied per hectare per year), enabled by the higher release rate for the aerosol dispersal (1900 kg_cat_ h^–1^) as opposed to typical crop dusters or boom spraying. They find *C*_open_ = $350–1060 tCO_2_e^–1^ for AQY_N2O_ = 0.01–0.03% and a tropospheric lifetime of 8 days. Higher-altitude release may yield a longer lifetime, yet with increased dispersal cost and a greater potential for widespread impacts. Any deployment format must also contend with the ultimate fate of the photocatalyst material, the difficulty of geographic containment, and the potential for long-term environmental effects. Because these considerations will constrain the deployment format, material selection, and monitoring requirements, they directly inform the achievable removal rates and net mitigation cost.

### 4.3 Ecosystem Uptake Enhancement

#### 4.3.1 Interventions

Ecosystem uptake enhancement has been defined as efforts to augment the in situ net uptake of greenhouse gases by or within primarily managed ecosystems but with potential to apply to natural systems.^37^ Here, interventions that suppress positive N_2_O fluxes are also considered because overlapping biological controls are relevant to atmospheric removal, mitigation of anthropogenic emissions, and suppression of natural emissions. See SI Section S10 for relevant background on the biological nitrogen cycle. Any attempt to modify natural environments must contend with both the difficulty of establishing and maintaining desired impacts and, conversely, the potential irreversibility of unintentional or unforeseen impacts.^104^

The exchange of nitrogen between inert dinitrogen (N_2_) in the atmosphere and reactive nitrogen species in soils and waters is mediated entirely by microbial activity in natural ecosystems.^105^ Within this cycle, N_2_O is a key component of the denitrification pathway, an anoxic respiratory process that evolved to replace oxygen as a terminal electron acceptor in respiration where O_2_ is limited or absent.^28,29^ Thus, denitrification reactions, including both the production and consumption of N_2_O, occur almost exclusively within oxygen-depleted microsites in soils or sediments. Nevertheless, heterogeneity within soil structures often allows sufficient atmospheric connectivity so that atmospheric N_2_O exchange occurs, albeit usually with net emission of N_2_O to the atmosphere. Recent advances in flux measurement using isotopic approaches have shown that gross N_2_O production and consumption within anaerobic soil microsites can be an order of magnitude larger than gross N_2_O exchanges with the atmosphere (uptake and emission).^31,32^

Efforts to enhance ecosystem uptake of N_2_O span three mechanistic paths: source suppression, which reduces gross N_2_O production; production-coupled consumption, which consumes locally produced N_2_O supported by elevated pore-space concentrations; and atmospheric consumption, which consumes N_2_O supplied from the atmosphere and remains active near atmospheric concentrations. The first two reduce a positive ecosystem flux, mitigating anthropogenic or natural emissions, but do not establish atmospheric removal: in soils, gross production and consumption are coupled, so as production falls, pore-space N_2_O concentration and consumption fall with it. Removal requires a distinct biological mechanism capable of atmospheric consumption sufficient to sustain inward transport from the atmosphere. Even a removal-capable intervention may function only as mitigation when deployed in a net-emitting ecosystem. Achieving removal is especially challenging because N_2_O reduction is strongly inhibited by O_2_ and therefore occurs in low-O_2_ microsites, which are poorly coupled to atmospheric exchange.

Five potential intervention classes have been identified: trace metal amendments, microbial inoculation, soil biogeochemistry, N_2_O fixation, and heterologous N_2_OR expression, summarized in Table 3 with evidence and analysis reported in SI Section S11. The evidence base is limited and insufficient to establish broadly achievable field performance. Reported fluxes from natural analogs are frequently based on short-duration measurements with small spatial extent and therefore may not reflect temporal persistence, weather sensitivity, or spatial heterogeneity when extrapolated to hectare- and year-scale net emissions.

**Table 3:** Interventions for Ecosystem Uptake Enhancement

**intervention**	**primary mechanism**	**reported flux (tCO**_**2**_ **e ha** ^**–1**^ **y^–^** ^**1**^ **) or efficacy** ^a^	**key limitation**	**mechanistic function and removal evidence**
trace metal amendments	supply trace metal cofactors for key N_2_O-controlling enzymes to modulate metabolic rate	Cu supplementation in wetland sediment reduced net N_2_O production by 80% and net CO_2_e emissions by 78% (including CO_2_ and CH_4_).^106^	highly variable results across studies; site-specific nutrient and dosing requirements.	production-coupled consumption demonstrated in lab; does not establish atmospheric consumption.
microbial inoculation	nondenitrifying N_2_O-reducers	kinetics indicate a gross consumption rate of 28 tCO_2_e ha^–1^ y^–1^ when extrapolated to atmospheric N_2_O concentration.^107^	respiration is strongly suppressed by O_2_ (lab), and field interventions show little effect during periods of low gross N_2_O production.	production-coupled consumption demonstrated in field; atmospheric consumption not observed.
	lichens (soil and tree bark communities)	instantaneous net ecosystem uptake of 0.5–2.5 tCO_2_e ha^–1^ y^–1^ observed in natural ecosystems.^108,109^	incomplete characterization of natural ecosystems; no demonstrated process for cultivating or replicating.	atmospheric consumption indicated by observed net uptake.
soil biogeochemistry	agroforestry, biochar and ground rock amendments to cropped fields shift soil pH, porosity, and moisture	net N_2_O emissions reduction of 0.01–0.6 tCO_2_e ha^–1^ y^–1^.^110,111^	intensive intervention for small mitigation potential; limited field data.	source suppression and production-coupled consumption demonstrated; atmospheric consumption unproven.
N_2_O fixation	modified nitrogenase enzyme to increase preferential fixation of N_2_O over N_2_	theoretical replacement of biological N_2_ fixation yields 1–2 (soils) to 100 (legumes) tCO_2_e ha^–1^ y^–1^.	high uncertainty, no known R&D efforts.	atmospheric consumption mechanism; removal potential remains theoretical.
heterologous N_2_OR expression	expressing N_2_OR in crop leaves	unknown efficacy due to highly oxygenated leaf environment.^112,113^	N_2_OR has been expressed in tobacco leaves but activity has not been confirmed in a relevant environment.	atmospheric consumption mechanism; removal potential remains theoretical.

a Detailed process descriptions and analyses with underlying field or laboratory evidence for each intervention are reported in SI Section S11.

Accounting of the net climate impact for ecosystem interventions must include CH_4_ and CO_2_ fluxes, and a verified counterfactual emissions profile.^114^ Nonetheless, in studies where all three greenhouse gases were measured, changes in the N_2_O flux are the primary driver of changes in the overall CO_2_e flux.^106,115^

The range of observed N_2_O and N_2_ fluxes in existing natural and managed ecosystems can bound the magnitude of potential intervention effects. Globally, cropland has a typical net N_2_O emission rate of 0–5 kg N_2_O–N ha^–1^ y^–1^ (0–2.1 tCO_2_e ha^–1^ y^–1^).^116^ Two reviews which focus only on field studies reporting net N_2_O uptake, by far the minority, report net uptake of 0.1–1 kg N_2_O ha^–1^ y^–1^ (0.03–0.27 tCO_2_e ha^–1^ y^–1^).^33,34^ Observations in forest soils report net uptake up to 2.5 tCO_2_e ha^–1^ y^–1^.^108^ Nondenitrifying N_2_O reducers could sustain atmospheric consumption of *R*_CO2e_ ≈ 28 tCO_2_e ha^–1^ y^–1^ from reported kinetics if O_2_ inhibition were overcome (SI Section S11).^107^ This entire range is far below the surface–atmosphere mass-transfer limit of ∼ 90 tCO_2_e ha^–1^ y^–1^ (SI Section S9.2), so the binding constraints are internal to the ecosystem: transport into anoxic microsites, metabolic energy availability, the concentration of N_2_O-consuming enzymes, and the frequency of suitable conditions. Interventions that exploit the greater atmospheric exchange of trees or crop leaves therefore offer little benefit unless they also relieve these internal limits.

#### 4.3.2 Cost Drivers and Performance Targets

A range of established methods disperse biological amendments in agricultural systems, including seed inoculation, foliar sprays, and soil amendments, as well as in natural ecosystem restoration, at reported costs of *C*_A_ = $13–67 ha^–1^ y^–1^ (SI Section S11). An intervention achieving *R*_CO2e_ = 0.2–2 tCO_2_e ha^–1^ y^–1^ would therefore yield *C*_open_ ≈ $7–340 tCO_2_e^–1^ (Figure 7).

Because many of these mechanisms are most amenable to agricultural systems, which are often net emitting, established N_2_O mitigation practices in those systems provide a relevant cost and feasibility benchmark. A recent global assessment estimates costs of approximately $24–185 tCO_2_e^–1^ across agricultural N_2_O measures, including improved fertilizer management, and an average of $61 tCO_2_e^–1^ for animal-diet, housing, and manure-storage measures.^9^ The *C*_open_ range estimated above overlaps these benchmarks, suggesting that ecosystem interventions could be cost-competitive when functioning as mitigation or could provide a distinct benefit where atmospheric consumption achieves net removal. Nevertheless, established practices set a high benchmark for demonstrated efficacy and acceptable risk, given the greater uncertainty and potential risks of these speculative, early stage approaches.

### 4.4 Scale and Deployment Considerations

Open-system interventions are subject to a broader range of deployment considerations because they directly interact with the environment, increasing both the consequence and the uncertainty of certain risks: impacts to air quality via the formation of unintended reactive species, loss of materials and byproducts into water and soil with downstream effects, and broader ecosystem perturbations. Further, the process of identification, measurement, isolation, and remediation of undesired impacts may be more difficult than for a closed system.

Cropland is a promising setting for controlled field research on open-system interventions because existing management infrastructure can reduce experimental cost and logistical burden relative to less-managed ecosystems. Elevated ground-level N_2_O from diffuse agricultural emissions can also increase the effectiveness of interventions, resulting in a combined mitigation and removal outcome. Tens of millions of acres of crops already use biological inoculants,^117^ and herbicides are applied across large agricultural areas (>85% of acreage for most crops in the United States).^118^ The existing scale of agricultural spraying and inoculant use provides a relevant benchmark for studying logistical scalability, but no N_2_O-removal intervention has yet demonstrated the efficacy, durability, environmental safety, or MRV needed for deployment. All of these interventions can likely be combined with existing spraying activities, and doing so could reduce *C*_open_ by approximately 50% for biological approaches (1 application per season) and 90% for photocatalyst powders (weekly to biweekly application). Although chemicals and biologics are already used widely in agriculture, interventions that directly interact with the food system carry heightened human health risk and may face additional regulation. Project creditability also requires low-cost, high-quality MRV: the cost estimate here for direct CO_2_, CH_4_, and N_2_O flux measurements is ∼$170 ha^–1^ y^–1^ (SI Section S12), substantially higher than MRV costs for many existing carbon credit projects for which cheaper soil measurements or other proxies are considered sufficient.^13^

Interventions on built surfaces have the potential to be both more durable (photocatalytic paints or lichen can last for years) and more reversible (surfaces can be repainted and have a greater degree of separation from natural environments which may suffer permanent consequences). However, the total surface area is significantly smaller than cropland, e.g., 3% of US land area was urban while 17% was cropland in 2017,^119^ and is fragmented, reducing the maximum potential scale. Proposals to exploit high-airflow surfaces do not address the binding constraint: realistic removal rates for both biological and photocatalytic approaches fall below the stationary-surface mass-transfer limit, so added airflow offers little benefit at current performance (Section 4.2.2).

Deployment in rangeland, forests, and other nonagricultural open land, 57% of US land, would triple the area for interventions compared to cropland,^119^ yet the area subject to annual spraying is smaller^120^ and dedicated aerial dispersal may be required. Photocatalyst deployment in arid and high-irradiation locations could increase the removal rate and the active lifetime of powders, for example enabling a roughly 10-fold cost reduction (SI Section S12). Even then, approximately 80–310 million ha would be required to achieve a 2 Mt N_2_O y^–1^ (0.55 Gt CO_2_e y^–1^) removal rate. While the logistics of deployment and MRV are broadly similar to those for cropland, greater caution must be taken, and a broader range of stakeholders involved, as the risk of irreversible ecosystem impacts is heightened.

Photocatalytic aerosols represent a conceptually different deployment class, sharing many environmental impact risks and social considerations with stratospheric aerosol injection (SAI) and other geoengineering approaches. Both have the potential for unintended impacts, and neither can be readily contained geographically (Section 4.2). Photocatalytic particles are designed for reactivity and may undergo unintended reactions in the atmosphere and, after deposition, in terrestrial and aquatic systems. Yet, to the extent that the risks of SAI and photocatalytic aerosols are differentiated, including both within a research portfolio increases the likelihood that a viable option emerges. The benefit of N_2_O removal is also long-lasting, avoiding SAI’s termination shock concern, and the long-term effect is to directly counteract the impact of elevated atmospheric N_2_O rather than to mask it, avoiding the residual impacts from SAI’s imperfect compensation of the multidimensional effects of rising atmospheric greenhouse gas levels.^121^

Maturing open-system N_2_O removal from isolated field trials to a scalable and governable climate intervention requires progress on five fronts: (i) validating removal rates under ambient conditions across multiple seasons and sites; (ii) durability engineering, including rainfastness and ecological persistence, with explicit quantification of activity half-lives; (iii) MRV, spanning lower-cost field flux systems, scalable approaches for small-area measurement, and foundational work toward aerosol MRV systems; (iv) risk-reduction-by-design, including suppression of byproducts and containment or reversibility strategies; and (v) stakeholder-aligned deployment pilots, beginning with cropland and built surfaces, that evaluate operational disruption, marginal logistical cost, and the conditions for social license alongside technical performance.

## 5. Current Status and Future Prospects

Given the limited potential of existing mitigation strategies for N_2_O emissions and the steep reductions required to align with a 2 °C warming scenario, research into the technical efficacy, climate viability, environmental and social viability, and economic plausibility of atmospheric N_2_O removal is warranted to determine whether it could serve as a distinct complement to emissions mitigation and CDR. While an emerging body of work has demonstrated N_2_O destruction via a wide range of chemical and biological mechanisms, substantial challenges remain to develop climate-beneficial and cost-effective approaches.

This analysis shows that feasibility is set not only by intrinsic reaction chemistry but by system boundaries that govern energy requirements, monitoring, environmental interaction, and cost. Framing feasibility this way enables consistent comparison across approaches that differ widely in maturity, scale, and operating context.

### 5.1 Comparative Assessment

Each approach assessment is synthesized in Table 4 to report the current status (feasibility level, key constraint) and highest-leverage research direction (viability threshold, key research need). Detailed justification for each assignment, including the cost estimates and their principal uncertainties (consolidated in Table S11) and how the evidence from Sections 3 and 4 was interpreted, is provided in SI Section S13.

**Table 4:** Status of Approaches and Research Directions

**approach**	**feasibility level 1–4** ^a^	**key constraint** ^b^	**viability threshold** ^c^	**key research need** ^d^
thermal reactor	1	high heating energy requirement	ε_HX_ = 99.9%	identify the cost-efficiency frontier of large high-efficiency heat exchangers.
thermocatalytic reactor	3	high heating energy requirement	*T*_rxn_ = 45 °C or ε_HX_ = 99.7% heat recovery (or a combination thereof)	high-throughput assay of thermal catalysts for reaction kinetics and H_2_O inhibition at atmospheric concentration.
photolytic reactor	1	O_3_ production limits AQY and results in hazardous emissions	in situ O_3_ treatment that maintains AQY_N2O_	development of a lab-scale experimental system with in situ O_3_ treatment.
gas-phase radical reactor	1	theoretical maximum AQY_N2O_ < 1 × 10^–8^	no path to viability through additional research or development has been identified	no targeted research is recommended.
photocatalytic reactor	1	high lighting energy requirement due to low AQY_N2O_	AQY_N2O_·η_rad_ > 0.63%	high-throughput assay of photocatalysts for AQY_N2O_, competing reactions, and humidity dependence at atmospheric concentration.
bioreactor	3	low removal efficiency under aerobic conditions	removal efficiency >90% for low-cost biofilter architecture	assay of natural and engineered communities for N_2_O reduction kinetics and O_2_ inhibition under controlled biofilter conditions.
photocatalytic surfaces	2	limited field data on performance and environmental impact; attribution of the reported removal not yet established	reported performance replicated across seasons and sites; positive environmental impact and toxicology assessment	field trials with comprehensive performance, longevity, and environmental impact monitoring across seasons and sites.
photocatalytic aerosols	2	no data on aerosol performance or environmental impact	small-scale outdoor test with intensive monitoring showing positive environmental impact and performance	paired chamber and small-scale outdoor testing with comprehensive measurement of gas-phase reactions.
ecosystem uptake enhancement	1	O_2_ inhibition of the primary biological N_2_O reduction pathway limits reaction with atmospheric N_2_O	season-average net N_2_O uptake (implying O_2_ tolerance) > 1 tCO_2_e ha^–1^ y^–1^ across multiple sites	identify or engineer bacterial communities that sustain N_2_O reduction under natural or managed field conditions.

a Feasibility level refers to the framework laid out in Section 2.2: (1) technical efficacy; (2) climate viability; (3) environmental and social viability; (4) economic plausibility.

b Key constraint refers to the single technical dimension that limits the feasibility of this approach at present.

c Viability threshold refers to a quantitative threshold for the key constraint that is required (but not alone sufficient) to demonstrate feasibility level 4/4 (i.e., *C*_net_ or *C*_open_ < $200 tCO_2_e^–1^ with reasonable other assumptions).

d The key research need is a near-term research topic that would advance the key constraint or reduce uncertainty on the current or future performance.

### 5.2 Key Research Priorities

A set of focused research efforts with high leverage for advancing performance and reducing uncertainty emerges from Table 4. For closed-system approaches, the nonbiological mechanisms have well-defined performance thresholds for climate viability, yet have received relatively little investigation at environmentally relevant conditions. These research efforts do not merit equal attention (Table S11): gas-phase radical reactors are ruled out by well-established kinetics, and even an ideal light source leaves photolytic reactors climate-beneficial but at roughly 2.5 times the energy target for cost-effectiveness, with mitigation of coproduced O_3_ an open question. Of the remaining efforts, the thermal-catalyst and photocatalyst assays offer the greatest leverage, as catalyst performance at atmospheric concentration is the key uncertainty for both. The 10-fold and 25- to 50-fold performance gains required for thermocatalytic and closed-system photocatalytic reactors are unlikely to be met, but neither mechanism is foreclosed: thermal destruction retains waste-heat and higher-concentration niches, and photocatalysis extends to open-system formats. Although a dedicated concentrator stage appears unlikely to improve closed-system economics (Section 3.7), characterizing N_2_O uptake on established CO_2_ sorbents could open an additional approach, coremoval at sorbent-based DAC facilities.

For biological and open-system approaches, the small existing body of knowledge on pilot reactors, field interventions, and natural analogs has high uncertainty, which can be reduced through benchmarking studies and continued field trials. Field-applied photocatalytic powders, bioreactors, and ecosystem uptake enhancement are the only approaches with reported removal at or near atmospheric concentration, yet single studies and short-duration observations make these preliminary indications rather than performance benchmarks. As these studies are necessarily of longer duration than laboratory assays, achieving comparable progress will require innovations that reduce monitoring cost and uncertainty and enable coordinated parallel experimentation. For the biological approaches, laboratory assays can screen natural and engineered communities for sustained N_2_O reduction under bulk aerobic conditions, the common constraint, ahead of season-long field campaigns. Across both system classes, additional characterization of the interactions with other greenhouse gases and harmful air pollutants is needed. Many photocatalytic processes can also destroy CH_4_ and other air pollutants, while biological systems present both risks and opportunities for coupled changes in N_2_O, CH_4_, and CO_2_ fluxes. Accounting for these interactions is essential for maximizing net climate impact and demonstrating environmental viability.

A diverse research portfolio remains essential for advancing N_2_O removal, as relatively bounded efforts can substantially reduce uncertainty across this family of approaches, each with fundamentally different constraints. Many of these mechanisms are also relevant to emissions mitigation, such that advances motivated by removal research may see broader applications. For this reason, foundational research should proceed even where long-term deployment pathways remain uncertain, including field studies of open-system approaches that are confined to managed environments and conducted within established regulatory frameworks. Overall, open-system approaches appear to have lower theoretical cost floors and fewer energy-related constraints than closed-system approaches, but face substantially greater challenges in measurement, environmental risk, and governance. Looking ahead, field-building efforts focused on feasibility assessment, broader environmental impacts, social license to operate, and MRV will be needed to guide future research and development. Resolving the mechanisms, constraints, and trade-offs identified here will determine whether atmospheric N_2_O removal can serve as a credible complement to emissions mitigation.

## Supplementary Material



## Notes

Sections covering: (1) reference values and calculations for atmospheric composition, greenhouse gas intensity, and currency and unit conversions; (2) derivation of the closed-system cost model; (3) analysis of thermal and thermocatalytic systems; (4) analysis on the availability of waste heat; (5) analysis of photolysis energy requirements; (6) analysis of photocatalytic reactors; (7) tabulated values for the aqueous solubility of greenhouse gases; (8) bioreactor references and analysis of swine-exhaust and multistage wastewater-treatment systems; (9) derivation of the open-system cost model, mass-transfer limit, equivalence of reaction rate and AQY under solar irradiance, analysis of updraft towers for artificial advection, and reference open-system removal rates; (10) an overview of the nitrogen cycle and biological mechanisms for N_2_O consumption; (11) details of potential interventions for ecosystem uptake enhancement; (12) additional analysis for the scale and deployment considerations for open-system approaches; and (13) assessment of each approach within the feasibility framework (PDF)
