# Supplementary material for: Atmospheric Removal of Nitrous Oxide: Approaches and Feasibility
Source: Environ Sci Technol. 2026 Oct 16;60(42):29753–69. doi: 10.1021/acs.est.6c04107 (PMC13718624; doi:10.1021/acs.est.6c04107)
Supplement: Supplementary file 1 [file es6c04107_si_001.pdf]

Supporting Information for:
Atmospheric Removal of Nitrous Oxide: Approaches and Feasibility

Sawyer, William J.[1]*

[1]Resolute Methane, Somerville, MA, USA 02143

*Corresponding Author: will@resolutemethane.com

Contains 53 pages, one figure, and eleven tables, within thirteen sections:

Section S1: Reference Values and Calculations (S2)
Section S2: Closed-System Cost Model (S8)
Section S3: Thermal and Thermocatalytic Analysis (S11)
Section S4: Availability of Waste Heat (S12)
Section S5: Photolysis Energy Requirements (S13)
Section S6: Photocatalytic Reactors (S17)
Section S7: Aqueous Solubility of Greenhouse Gases (S22)
Section S8: Bioreactor References (S22)
Section S9: Open-System Cost Model (S24)
Section S10: The Nitrogen Cycle and Biological Mechanisms for N_2O Consumption (S28)
Section S11: Potential Interventions for Ecosystem Uptake Enhancement (S30)
Section S12: Additional Analysis for Section 4.4 (S34)
Section S13: Additional Analysis for Section 5 (S34)
References (S43)

S1. Reference Values and Calculations

S1.1. Atmospheric Composition and GWP Values

Table S1 reports atmospheric composition and gas properties for the major constituents of dry air plus all greenhouse gases with an effective radiative forcing of >0.1 mW m^{-2} (relative to a pre-industrial baseline of 1750).[1] Global warming potential (GWP) is typically reported on a relative basis by normalizing the absolute global warming potential (AGWP) of a gas species to that of CO_2 by mass. To compare the gross CO_2-equivalent removal value of gases in an ambient-air inlet stream, conventional mass-based GWP_{100} is converted to a molar basis by multiplying by the ratio of the gas molar mass to the molar mass of CO_2. Multiplying each atmospheric mole fraction by its molar GWP_{100} gives a GWP_{100}-weighted molar concentration in ppm CO_2e. This inlet-stream metric represents the gross value of complete removal and is not a measure of current radiative forcing. Unless otherwise stated, all calculations in this work assume 25 °C and 1 atm.

Table S1. Atmospheric composition and gas properties

	**M_m[a]**	**τ_{atm}[b]**	**$AGWP_{100}$[b]**	**GWP_{100}**		**Pre-industrial concentration**		**Present concentration**	
**Species**	**(g mol^{-1})**	**(y)**	**(pW y m^{-2} kg^{-1})**	**(kg CO_2e kg^{-1})**	**(mol CO_2e mol^{-1})**	**(ppm)[c]**	**(ppm CO_2e)**	**(ppm)[c]**	**(ppm CO_2e)**
				Major components:					
Nitrogen (N_2)	28.01							780840[d]	
Oxygen (O_2)	32.00							209470[d]	
Argon (Ar)	39.95							9340[d]	
Neon (Ne)	20.18							18.2[d]	
Helium (He)	4.00							5.2[d]	
Krypton (Kr)	83.80							1.1[d]	
Hydrogen (H_2)	2.02							0.53[d]	
Carbon monoxide (CO)	28.01							0.10[d]	
Xenon (Xe)	131.29							0.09[d]	
Ozone (O_3)	48.00							0.07[d]	
Nitrogen dioxide (NO_2)	46.01							0.02[d]	
Iodine (I_2)	253.81							0.01[d]	
				Primary greenhouse gases:					
Carbon dioxide (CO_2)	44.01	Var[e]	0.0895	1.0	1.0	278	278	428[f]	428

Methane (CH_4)	16.04	11.8	2.49	27.8	10.1	0.73	7.4	1.94[f]	19.7
Nitrous oxide (N_2O)	44.01	109	24.5	273	273	0.270	74	0.339[f]	92.5
Halogenated greenhouse gases:									
CFC-12 (CCl_2F_2)	120.91	102	1120	12514.0	34381.1	0	0	5.03×10^{-4}	17.3
CFC-11 (CCl_3F)	137.37	52	557	6223.5	19425.2	0	0	2.26×10^{-4}	4.39
CFC-113 (CCl_2FCClF_2)	187.38	93	583	6514.0	27733.6	0	0	6.98×10^{-5}	1.94
PFC-14 (CF_4)	88.01	50000	660	7374.3	14746.1	3.41×10^{-5}	0.5	8.55×10^{-5}	1.26
HCFC-22 ($CHClF_2$)	86.47	11.9	175	1955.3	3841.7	0	0	2.47×10^{-4}	0.95
Sulfur hexafluoride (SF_6)	146.06	1000	2180	24357.5	80834.8	0	0	9.95×10^{-6}	0.80
HFC-23 (CHF_3)	70.01	228	1310	14636.9	23285.3	0	0	3.24×10^{-5}	0.75
Carbon tetrachloride (CCl_4)	153.82	32	196	2189.9	7654.3	3.00×10^{-8}	2.30×10^{-4}	7.79×10^{-5}	0.60
CFC-114 ($CClF_2CClF_2$)	170.92	189	844	9430.2	36623.8	0	0	1.60×10^{-5}	0.59
HFC-134a (CH_2FCF_3)	102.03	14	137	1530.7	3548.8	0	0	1.08×10^{-4}	0.38
HFC-125 (CHF_2CF_3)	120.02	30	335	3743.0	10207.7	0	0	2.94×10^{-5}	0.30
CFC-115 ($CClF_2CF_3$)	154.47	540	859	9597.8	33686.2	0	0	8.67×10^{-6}	0.29
HFC-143a (CH_3CF_3)	84.04	51	520	5810.1	11094.7	0	0	2.40×10^{-5}	0.27
PFC-116 (C_2F_6)	138.01	10000	1110	12402.2	38892.2	0	0	4.85×10^{-6}	0.19
CFC-13 ($CClF_3$)	104.46	640	1450	16201.1	38453.8	0	0	3.28×10^{-6}	0.13
HCFC-142b (CH_3CClF_2)	100.50	18	205	2290.5	5230.3	0	0	2.23×10^{-5}	0.12
PFC-318 (c-C_4F_8)	200.03	3200	912	10189.9	46314.3	0	0	1.75×10^{-6}	8.11×10^{-2}
Halon-1301 ($CBrF_3$)	148.91	72	644	7195.5	24346.4	0	0	3.32×10^{-6}	8.08×10^{-2}
Nitrogen trifluoride (NF_3)	71.00	569	1560	17430.2	28120.4	0	0	2.05×10^{-6}	5.76×10^{-2}
HCFC-141b (CH_3CCl_2F)	116.95	9.4	77	860.3	2286.2	0	0	2.44×10^{-5}	5.58×10^{-2}
CFC-114a (CCl_2FCF_3)	170.92	105	664	7419.0	28813.0	0	0	1.05×10^{-6}[g]	3.03×10^{-2}
PFC-218 (C_3F_8)	188.02	2600	831	9284.9	39666.9	0	0	6.80×10^{-7}	2.70×10^{-2}
Sulfuryl fluoride (SO_2F_2)	102.06	36	414	4625.7	10727.2	0	0	2.50×10^{-6}	2.68×10^{-2}

Halon-1211 ($CBrClF_2$)	165.37	16	173	1933.0	7263.0	0	0	3.28×10^{-6}	2.38×10^{-2}
HFC-227ea (CF_3CHFCF_3)	170.03	36	322	3597.8	13899.7	0	0	1.59×10^{-6}	2.21×10^{-2}
HFC-32 (CH_2F_2)	52.02	5.4	69	770.9	911.3	0	0	2.00×10^{-5}	1.82×10^{-2}
PFC-5-1-14 (n-C_6F_{14})	338.04	3100	771	8614.5	66168.4	0	0	2.20×10^{-7}[g]	1.46×10^{-2}
CFC-113a (CCl_3CF_3)	187.38	55	351	3921.8	16697.2	0	0	6.20×10^{-7}[g]	1.04×10^{-2}
PFC-31-10 (n-C_4F_{10})	238.03	2600	897	10022.3	54205.6	0	0	1.90×10^{-7}[g]	1.03×10^{-2}
CFC-112 (CCl_2FCCl_2F)	203.83	63.6	413	4614.5	21371.9	0	0	4.20×10^{-7}[g]	8.98×10^{-3}
HFC-245fa ($CHF_2CH_2CF_3$)	134.05	7.9	86.1	962.0	2930.1	0	0	3.06×10^{-6}	8.97×10^{-3}
PFC-41-12 (n-C_5F_{12})	288.04	4100	825	9217.9	60328.8	0	0	1.40×10^{-7}[g]	8.45×10^{-3}
PFC-61-16 (C_7F_{16})	388.05	3000	752	8402.2	74085.0	0	0	1.10×10^{-7}[g]	8.15×10^{-3}
PFC-71-18 (C_8F_{18})	438.06	3000	739	8257.0	82186.5	0	0	9.00×10^{-8}[g]	7.40×10^{-3}
Halon-2402 ($CBrF_2CBrF_2$)	259.82	28	194	2167.6	12796.9	0	0	4.00×10^{-7}	5.12×10^{-3}
Methyl chloride (CH_3Cl)	50.49	0.9	0.495	5.5	6.3	4.57×10^{-4}	2.90×10^{-3}	5.51×10^{-4}	3.50×10^{-3}
HFC-365mfc ($CH_3CF_2CH_2CF_3$)	148.08	8.9	81.7	912.8	3071.4	0	0	1.09×10^{-6}	3.35×10^{-3}
HFC-43-10mee ($CF_3CHFCHFCF_2CF_3$)	252.05	17	143	1597.8	9150.6	0	0	2.90×10^{-7}	2.65×10^{-3}
HCFC-124 ($CHClFCF_3$)	136.48	5.9	53.4	596.6	1850.2	0	0	1.02×10^{-6}[g]	1.89×10^{-3}
HFC-152a (CH_3CHF_2)	66.05	1.6	14.7	164.2	246.5	0	0	7.10×10^{-6}	1.75×10^{-3}
Methylene chloride (CH_2Cl_2)	84.93	0.493	0.998	11.2	21.5	7.00×10^{-6}	1.51×10^{-4}	4.10×10^{-5}	8.82×10^{-4}
Methyl chloroform (CH_3CCl_3)	133.40	5	14.4	160.9	487.7	0	0	1.60×10^{-6}	7.80×10^{-4}
Chloroform ($CHCl_3$)	119.38	0.501	1.84	20.6	55.8	4.80×10^{-6}	2.68×10^{-4}	8.80×10^{-6}	4.91×10^{-4}
HCFC-133a (CH_2ClCF_3)	118.49	4.6	34.7	387.7	1043.8	0	0	4.00×10^{-7}[g]	4.18×10^{-4}
						Total halogenated:	0.5		30.8

[a]Molecular weight data from the CRC Handbook of Chemistry and Physics.[2]
[b]Atmospheric lifetime and absolute global warming potential at 100-year time horizon

(AGWP$_{100}$) from Smith et al. unless otherwise noted.[3]
[c]Atmospheric concentrations from Dentener et al., unless otherwise noted, where pre-industrial refers to 1750 and current refers to 2019.[1]
[d]Present-day major component concentrations from NOAA.[4]
[e]For carbon dioxide, no single lifetime has been defined as there are multiple removal processes with rates ranging from decades to centuries. About half of carbon dioxide emitted today will be gone in a century, but a portion will persist for millennia.[5]
[f]CO_2, CH_4, and N_2O concentrations are "September 2025 monthly mean value corrected for seasonal variation" as reported by NOAA Global Monitoring Lab.[6]
[g]Atmospheric concentrations in 2015 from Dentener et al.[1]

Figure 1 shows the global temperature response for a pulse emission of 1 t N_2O and a GWP$_{100}$-equivalent 273 t CO_2, normalized to the peak temperature deviation of the CO_2 response. Temperature responses are calculated following eq 8.SM.14 and 8.SM.15 in Myhre et al. Supplementary Material[7] using radiative forcing values from Smith et al.[3]

Figure 2 was produced using the GWP$_{100}$-weighted molar concentrations reported in Table S1. The value shown for each gas is its present-day GWP$_{100}$-weighted molar concentration in ambient air (ppm CO_2e). These values sum to 571 ppm across the gases in Table S1, and each gas's share of that total is its share of gross inlet-stream removal value under the assumption of complete removal.

S1.2. Currency and Unit Conversions

All monetary values reported in literature are converted to United States Dollars using the US Treasury's historical Rates of Exchange dataset[8] and inflation adjusted from the year of publication to 2025 using the Federal Reserve Bank of Minneapolis' inflation calculator, based on the CPI-U.[9] Where the adjusted value has more significant figures than the reported value, it has been additionally rounded to the same number of significant figures.

Table S2. Currency and unit conversions

Section	Text	Original value	Original unit	Adjusted value	Rounded value
1	"…the U.S. Environmental Protection Agency (EPA) estimates damages (social cost) of $67,000 t^{-1} of N_2O, compared to $240 for CO_2 and $2,000 for CH_4…"	$54,000 t$N_2O^{-1}$	2020 USD	$67,241 [or, using GWP$_{100}$ = 273, $246.30 t$CO_2e^{-1}$]	$67,000 [or $250 t$CO_2e^{-1}$]
1		$190 t$CO_2^{-1}$	2020 USD	$237	$240
1		$1,600 t$CH_4^{-1}$	2020 USD	$1,992	$2,000
1	"This effect adds an estimated $2,420 t^{-1} in human health and	$2.66 kg N^{-1} → $1.69 kg N_2O^{-1} → $1,690 t N_2O^{-1}	2011 USD	$2,420	$2,420

	agricultural impacts…"				
1	"…estimated costs of approximately \$6–23 tCO_2e^{-1}…"	The 2024 Global Nitrous Oxide Assessment reports \$1,600–6,000 t N_2O^{-1}, equivalent to \$5.86–21.98 t CO_2e^{-1} using GWP_{100} = 273.	2024 USD	Using CPI-U (2025/2024 = 967.5/942.7), this is \$1,642.09–6,157.84 t N_2O^{-1}, equivalent to \$6.01–22.56 t CO_2e^{-1}.	\$6–23 tCO_2e^{-1}
2.2	"…informed by the US EPA's social cost of N_2O at \$250 tCO_2e^{-1} (similar to CO_2's \$240 tCO_2e^{-1})…"	See row 1			
2.2	"…the \$0.1–160 tCO_2e^{-1} range of compliance market carbon prices."	\$0.1 to \$158.8 tCO_2e^{-1}	2025 USD	No adjustment	\$0.1–160 tCO_2e^{-1}
SI 2	"…the total cost of major equipment is \$469–535 million and the cost of the air contactor is \$136–309 million in 2025 dollars (Table S2)."	\$394–450 million, major equipment	2021 USD	\$469–535 million	\$469–535 million
SI 2		\$114–260 million, air contactor	2021 USD	\$136–309 million	\$136–309 million
SI 3	"…total capital cost ranging from \$8.46 million for a 250,000 m^3 h^{-1} system to \$26.44 million for a 1,000,000 m^3 h^{-1} system."	\$8 million	2023 USD	\$8.46 million	\$8.46 million
SI 3		\$25 million	2023 USD	\$26.44 million	\$26.44 million
SI 6	"Randall et al. estimate a lower-bound capital cost of \$0.074 kWh^{-1} for LED modules…"	\$0.074 kWh^{-1}	2023 USD	\$0.078 kWh^{-1}	\$0.078 kWh^{-1}
SI 6	"Their analysis assumes each LED costs \$5.29…"	\$5 per LED module	2023 USD	\$5.29	\$5.29
SI 6	"…achieving modeled lighting costs of order \$100–200 tCO_2e^{-1} therefore requires an apparent quantum yield of order 1%."	Figure 6 in Randall et al. shows a lighting cost of \$100–200 at AQY_{N2O} = 1%.	2023 USD	\$105–211	\$100–200
SI 8	"This cost estimate uses the reported materials cost as the capital cost,	\$1,350 reported materials cost; \$1.23 cfm^{-1}; \$0.32 d^{-1} consumables	2011 USD	\$1,930.50; \$1.76 cfm^{-1}; \$0.458 d^{-1}	\$1,930; \$1.76 cfm^{-1}; \$0.46 d^{-1}

	inflation-adjusted to 2025…"				
SI 8	"…a gross removal cost above €3,710 tCO_2e^{-1}."	€3,710 tCO_2e^{-1}	2022 EUR	$3,964 in 2022 USD, then $4,365.16 in 2025 USD	>$4,370 tCO_2e^{-1}
4.2.2	"…the deployment cost is low (e.g., $20 ha^{-1} for existing agricultural powder dispersal)…"	$8.01 $acre^{-1}$ average for self-propelled sprayer (Indiana Farm Survey)	2022 USD	$8.81 $acre^{-1}$ or $21.77 ha^{-1}	$20 ha^{-1}
4.2.2	"They estimate a dispersal cost of $0.80 kg^{-1}…"	$0.76 kg^{-1}	2023 USD	$0.804 kg^{-1}	$0.80 kg^{-1}
4.2.2	"…They find C_{open} = $350–1,060 tCO_2e^{-1} for AQY_{N2O} = 0.01–0.03%…"	$333–1,000 tCO_2e^{-1}	2023 USD	$351.84–1,056.58 tCO_2e^{-1}	$350–1,060 tCO_2e^{-1}
SI 9.5	"The lower-cost case uses the Telangana government Schedule of Rates for installed cool-roof coating, $3.84 m^{-2}."	₹300 m^{-2}	2023 INR	$3.84 m^{-2}	$3.84 m^{-2}
SI 9.5	"The upper-cost case uses the installed cost in the United States commercial-roof model of Randall et al., $18.8 m^{-2}."	$17.8 m^{-2}	2023 USD	$18.8 m^{-2}	$18.8 m^{-2}
SI 9.5	"Both cases use the median maintenance cost observed by Sproul et al., $0.27 m^{-2} y^{-1}."	$0.20 m^{-2} y^{-1}	2014 USD	$0.27 m^{-2} y^{-1}	$0.27 m^{-2} y^{-1}
SI 9.5	"…the washing assumption in Randall et al. ($2.1 m^{-2} y^{-1})…"	$2.0 m^{-2} y^{-1}	2023 USD	$2.1 m^{-2} y^{-1}	$2.1 m^{-2} y^{-1}
SI 11	"…to improve N_2 fixation in soy at a total cost of $17–24 ha^{-1}"	$12–17 ha^{-1}	2011 USD	$17.19–24.36 ha^{-1}	$17–24 ha^{-1}
SI 11	"all-in costs, including application labor and equipment, of $30–52 ha^{-1}"	$11.50 $acre^{-1}$ → $28.42 ha^{-1}	2023 USD	$30.06 ha^{-1}	$30 ha^{-1}
SI 11		$20 $acre^{-1}$ → $49.42 ha^{-1}	2023 USD	$52.27 ha^{-1}	$52 ha^{-1}

SI 11	"…small-scale academic trials reporting costs of \$450–4,170 ha^{-1} for manual collection…"	\$350–3,250 ha^{-1}	2018 USD	\$449.19–4,171.07 ha^{-1}	\$450–4,170 ha^{-1}
SI 12	"The cost of a single tower, which can cover an area of 100 ha, is estimated at \$160,000 (2022 Australian dollars)."	A\$160,000	2022 AUD	\$114,000	\$114,000
4.3.2	"…costs of approximately \$24–185 tCO_2e^{-1} across agricultural N_2O measures…"	\$23–180 tCO_2e^{-1}	2024 USD	\$23.61–184.74 tCO_2e^{-1}	\$24–185 tCO_2e^{-1}
4.3.2	"…an average of \$61 tCO_2e^{-1} for animal-diet, housing, and manure-storage measures."	\$59 tCO_2e^{-1}	2024 USD	\$60.55 tCO_2e^{-1}	\$61 tCO_2e^{-1}

S2. Closed-System Cost Model

The techno-economic model estimates the net cost of atmospheric greenhouse gas removal in units of US dollars per net metric ton of CO_2 equivalent (\$ tCO_2e^{-1}). The calculation is based on normalizing both costs and climate benefits to the volumetric flow of processed air. This model is intentionally simplified, incorporating just the system capital cost and the cost and emissions associated with energy use. These two elements are assumed to dominate the cost and life-cycle climate impact. A low-fidelity model is sufficient for the purpose of this assessment and the precise data that would be needed for a more complete model does not exist. Given that additional factors would likely both increase the total cost and decrease the net climate benefit, this model represents a lower bound on the net cost of removal. For example, any approach will have operating costs beyond just electricity use, and the capital equipment and end-of-life decommissioning have associated greenhouse gas emissions.

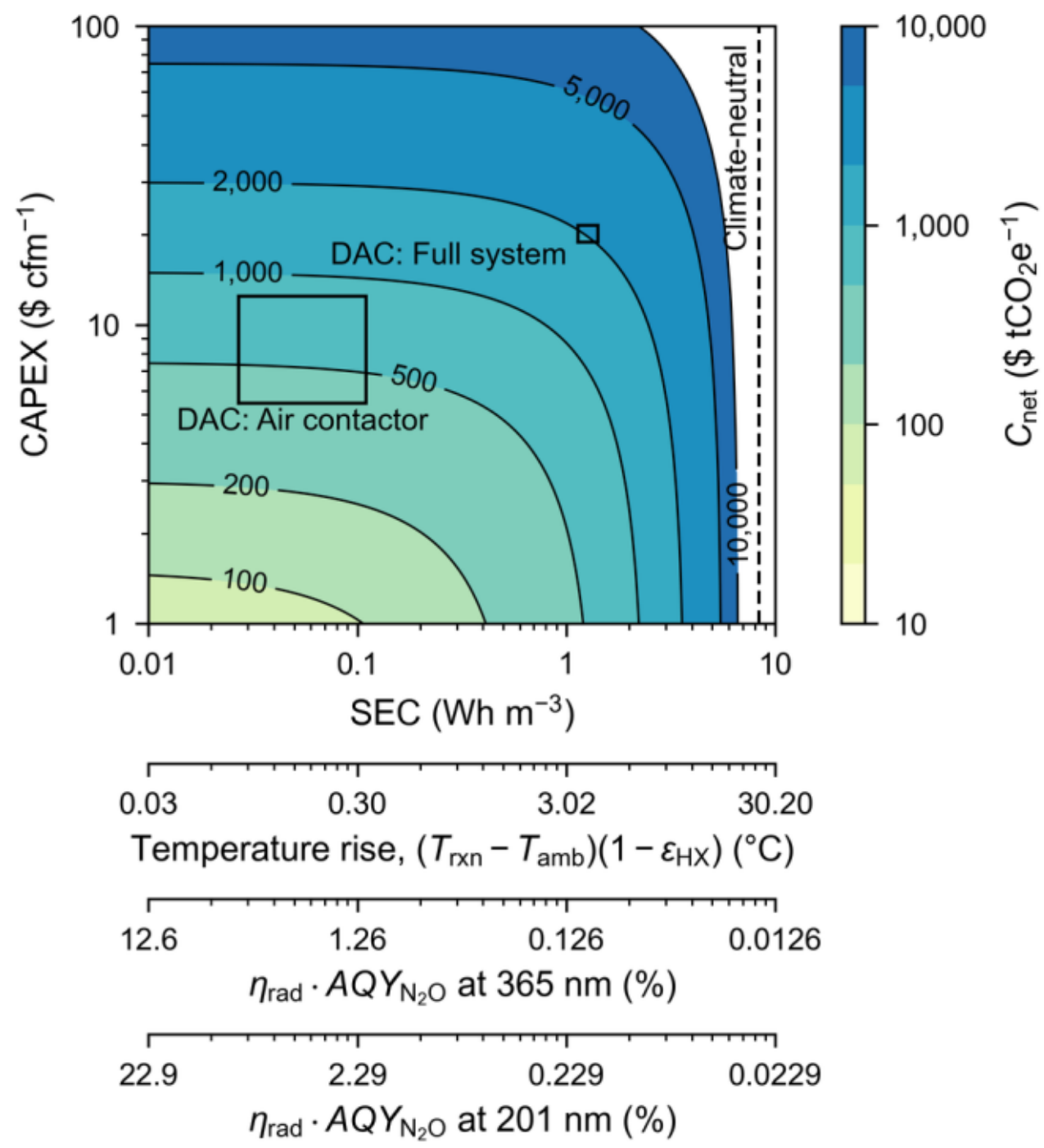

Figure S1. Total cost of atmospheric removal of N_2O per net tCO_2e using a closed system (Figure 3) including additional axes relating SEC to performance metrics for thermal and photon-based approaches.

The net cost of removal is defined as:

$$C_{net} = \frac{C_{cap,ann} + SEC \cdot p_{el}}{M_{CO2e} - SEC \cdot EF_{el}} \quad \text{(S1)}$$

where $C_{cap,ann}$ is the annualized capital cost per cubic meter of air processed ($ m^{-3}), SEC is the specific energy consumption (Wh m^{-3} air), p_{el} is the cost of electricity ($ kWh^{-1}), EF_{el} is the electricity emissions factor (tCO_2e kWh^{-1}), and M_{CO2e} is the gross removal of the process (tCO_2e m^{-3} air). The specific energy consumption is converted from Wh to kWh when evaluating eq S1. The volumetric properties of air are calculated assuming 25 °C and 1 atm. This analysis assumes the use of a renewable electricity source with EF_{el} = 20 g CO_2e kWh^{-1} (2×10^{-5} tCO_2e kWh^{-1}) representing the life-cycle emissions of a typical new utility-scale solar photovoltaic system in the United States[10] and p_{el} = $0.05 kWh^{-1}, representing a balance between present-day prices for industrial customers ($0.08 kWh^{-1} 2024 US average, in 2025 dollars)[11] and projected cost decreases (2050 LCOE projections down to $0.015–0.03 kWh^{-1}).[12] $C_{cap,ann}$ is determined by normalizing the capital cost to airflow capacity and applying a capital recovery factor (CRF, tabulated in Table S3):

$$C_{cap,ann} = \frac{CAPEX \cdot CRF}{V} \quad \text{(S2)}$$

$$CRF = \frac{r(1+r)^N}{(1+r)^N - 1} \quad \text{(S3)}$$

where CAPEX is the total capital investment normalized to the air-handling capacity ($\$$ cfm^{-1}), V is the volume of air handled per year normalized to the air-handling capacity (m^3 cfm^{-1} y^{-1}) determined using a capacity factor (*CF*), *N* is the economic lifetime of the system (y), and *r* is the discount rate (fractional). For this analysis, default values are: *CF* = 0.9, *N* = 10 y, and *r* = 0.08.

Table S3. Capital recovery factor (CRF) for annualized value of capital

	Discount rate (%)				
Period (y)	2%	4%	6%	8%	10%
2	0.52	0.53	0.55	0.56	0.58
3	0.35	0.36	0.37	0.39	0.40
4	0.26	0.28	0.29	0.30	0.32
6	0.18	0.19	0.20	0.22	0.23
8	0.14	0.15	0.16	0.17	0.19
10	0.11	0.12	0.14	0.15	0.16

The gross removal potential is derived from the ambient concentration of N_2O and its global warming potential:

$$M_{CO2e} = \chi \cdot c_{air} \cdot M_{mol} \cdot GWP \cdot X \quad \text{(S4)}$$

where χ is the mole fraction of N_2O (0.339 ppm), c_{air} is the molar concentration of air at 25 °C and 1 atm (40.92 mol m^{-3}), M_{mol} is the molar mass of N_2O (44.013 g mol^{-1}, 4.4013 × 10^{-5} t mol^{-1}), GWP is the mass-based global warming potential (unitless), and X is the removal efficiency of the process (unitless). For this analysis, default values are GWP = 273 (100-year time horizon, GWP_{100}) and X = 1 (complete removal). As SEC and CAPEX may vary widely across approaches, C_{net} is evaluated parametrically over the range SEC = 0.01–10 Wh m^{-3} air and CAPEX = $\$$1–100 cfm^{-1} and visualized as a contour plot in Figure 3.

Three additional sets of labels for the horizontal axis of Figure S1 are plotted to show the relationship between thermo(catalytic) and photon-based performance metrics and SEC. For the temperature rise, the temperature-dependent heat capacity of dry air is used to calculate the resulting temperature rise from 25 °C as a function of SEC. Full details of this calculation are described in Section S3. The quantum yield labels are the product of AQY_{N2O} and η_{rad} at 365 nm (representative of a photocatalytic process) and 201 nm (representative of a photolysis

process). The molar concentration of N_2O is used to convert SEC from units of Wh m^{-3} to Wh mol N_2O^{-1}. The energy of 1 mole of photons is calculated as:

$$E = \frac{h \cdot c}{\lambda} N_a \quad \text{(S5)}$$

where h is Planck's constant (6.626 × 10^{-34} J s), c is the speed of light (3 × 10^8 m s^{-1}), N_a is Avogadro's number (6.02 × 10^{23} mol^{-1}), and λ is the photon wavelength. Dividing E by SEC returns the number of N_2O molecules per photon, assuming 100% conversion of supply energy to photons. Full details of this calculation are described in Section S6.

For reference, a range of values corresponding to a 1 Mt CO_2 y^{-1} DAC system for CO_2 has been included based on a cost-performance model for solvent-based capture with thermal regeneration.[13] This source was selected because it contains sufficient information to break out the portion of cost and energy use for the air contactor alone. This system is assumed to be a well-optimized state-of-the-art design in 2021, but should not be assumed representative of the current-day state of the art or deployed systems given the wide range of DAC technologies and rapid pace of advancement.

For a range of variations of the total plant, the total cost of major equipment is $469–535 million and the cost of the air contactor is $136–309 million in 2025 dollars (Table S2).[13] A Lang factor of five is used to estimate total capital cost ranges from these values: $2.35–2.68 billion and $0.68–1.55 billion, respectively. Total power requirement is reported as 240–300 MW.[13] The plant's airflow is estimated at 58,770 m^3 s^{-1} (125 million cfm) based on the gross removal of 1 Mt CO_2 y^{-1}, the atmospheric mass concentration of CO_2, and a 70% capture fraction. Capital cost and total plant energy use are normalized by this estimated airflow to units of $ cfm^{-1} and Wh m^{-3}, respectively. Finally, the energy requirement of the air contactor is estimated based on a reported pressure drop of 68–280 Pa and an estimated blower efficiency of 0.7.[13]

S3. Thermal and Thermocatalytic Analysis

A 2025 UNECE report on the cost of RTO systems for the treatment of coal mine ventilation air indicates total capital cost ranging from $8.46 million for a 250,000 m^3 h^{-1} system to $26.44 million for a 1,000,000 m^3 h^{-1} system.[14] These flow rates, converted to cfm, are 147,000 and 589,000, respectively. These costs are adjusted to 2025 USD in Table S2. Thus, CAPEX = $44–58 cfm^{-1}.

The case study catalyst has a composition of $Zn_{0.36}Co_{0.64}Co_2O_4$, and was reported to have 100% N_2O destruction at a space velocity of 15,000 h^{-1} at 200–300 °C depending on the level of oxygen and moisture present.[15] The authors report this space velocity based on a catalyst mass of 0.6 g in a gas flow of 100–150 mL min^{-1}, equivalent to 0.6 g per 0.003531–0.005297 cfm or 170 to 113 g of catalyst cfm^{-1}.

Taking 113 g of catalyst cfm^{-1} as a lower bound, the lower bound for the catalyst cost based on the cost of the metals within is estimated at \$3.39 cfm^{-1} (Table S4).

Table S4. Cost of $Zn_{0.36}Co_{0.64}Co_2O_4$ catalyst

	Molar mass (g mol^{-1})	Mass in 1 mol catalyst (g)	Mass fraction in catalyst	Mass in 113 g catalyst (g)	Metal spot price (\$ kg^{-1})[a]	Cost in 113 g catalyst (\$)
Zn	65.38	23.54	0.0968	10.94	\$3.29	\$0.04
Co	58.93	155.58	0.6399	72.31	\$46.30	\$3.35
O	16.00	64.00	0.2633	29.75		
$Zn_{0.36}Co_{0.64}Co_2O_4$		243.12				\$3.39

[a]Metal spot prices are 2025 annual average spot prices as reported by the USGS Mineral Commodity Summaries for North America (Zinc)[16] and the US market (Cobalt).[17]

The energy required to heat air from ambient temperature to a desired temperature is:

$$SEC_{heat} = (1 - \varepsilon_{HX})\, f \int_{T_i}^{T_{rxn}} c_p(T)\, dT \quad \text{(S6)}$$

where $c_p(T)$ is the temperature-dependent specific heat (J kg^{-1} K^{-1}, as reported in Lienhard and Lienhard),[18] T_i is the ambient temperature, T_{rxn} is the desired temperature, ε_{HX} is the heat recovery efficiency, and f is a conversion factor from J kg^{-1} to Wh m^{-3} at 25 °C and 1 atm.

Equation S6 makes explicit a trade-off between operating temperature and heat recovery. SEC and CAPEX vary inversely with the two main determinants of system performance, ε_{HX} and T_{rxn}: ε_{HX} can be raised by enlarging the heat exchanger, and T_{rxn} can be lowered by increasing reactor residence time or catalyst loading. Both reduce energy use at the cost of additional capital, and comparable trade-offs may exist across catalysts and heat-recovery architectures. Because no cost basis is available for these variations, Section 3.2.2 evaluates fixed representative values rather than optimizing over them.

S4. Availability of Waste Heat

It is estimated that 72% of global primary energy is ultimately lost as waste heat, typically in the form of hot air.[19] Assuming a reaction temperature of 200 °C, the utilization of this resource can be maximized by diluting higher-temperature streams with ambient air to increase the volume of air processed. A study of waste heat in the EU estimates a total of 202 TWh y^{-1} at temperatures

above 200 °C,[20] equivalent to 3,500 km^3 y^{-1} of 200 °C air or 577,000 tCO_2e y^{-1} of N_2O, approximately 0.1% of the total N_2O emissions gap. Reducing the reaction temperature to 100 °C could increase this value by a factor of three through a combination of unlocking additional waste heat in the 100–200 °C range (~100 TWh y^{-1}) and increased dilution to reach the lower target temperature. While not insignificant, the scale of this opportunity may not be sufficient alone to justify the cost of research and development for this approach. While the use of waste heat reduces the cost of energy and the attributable energy-related emissions, it will also require reactor systems tailored to a large variety of relatively small installations. The addition of heat recovery could increase the volume of accessible air, but at the cost of increased CAPEX.

S5. Photolysis Energy Requirements

While the stratospheric photolysis and $O(^1D)$ pathways are well understood, to my knowledge there have been no efforts to replicate either in an engineered system. In either case, the initial energy source is a UV photon, and the key metric for determining the energy requirement is the fraction of all photons which result in an N_2O decomposition. N_2O's peak UV absorption is in the 180–240 nm range. Several other components of ambient air have non-negligible absorption in this wavelength range. For each species the photon absorption rate is defined as:

$$a_i = I \sigma_i A_i \tag{S7}$$

where I is the incident photon density (photon m^{-2} s^{-1}), σ_i is the absorption cross section of species i (m^2 $molecule^{-1}$), and A_i is the number concentration of species i (molecule m^{-3}). Assuming all photons are absorbed by one of these species, the fraction of photons that are absorbed by species i, the absorption yield, is defined as:

$$\gamma_i = \frac{a_i}{\sum_i a_i} = \frac{\sigma_i A_i}{\sum_i (\sigma_i A_i)} \tag{S8}$$

Because σ_i can vary strongly with wavelength, γ_i must be evaluated as a function of wavelength. The gases O_2, O_3, H_2O, CO_2, and N_2O are included in this analysis due to their relatively strong absorbance in this wavelength band and high concentration in tropospheric air. Among other main and trace components of air, CH_4, CO, NO, NO_2, and I_2 have non-negligible absorption cross sections, yet are not included due to their low $\sigma_i A_i$ across this wavelength band.

Table S5. Gas species and absorption cross sections

Gas	Concentration (ppm)	Absorption cross section reference
O_3	0.01	Table 4A-2-2 (185–295 nm)[21]<br>Figure 8 (180–185 nm)[22]
O_2	209,000	Table 4A-1-2 (205–245 nm)[21]<br>Figure 5 (180–205 nm)[22]

H_2O	11,500	Table 4B-2-2 (121–190 nm)[21] (190–230 nm)[23]
N_2O	0.339	Table 4C-3-2 (160–240 nm)[21]
CO_2	428	(170–300 nm)[24]

Figure 5 shows (a) raw absorption cross sections and (b) calculated absorption yields for typical tropospheric concentrations and 1.1 mol% water vapor (~37% RH at 25 °C). The maximum N_2O absorption yield is 0.48% at 201 nm. For relative humidity ranging from 0% to 100% the maximum absorption yield varies from 0.66% (200 nm) to 0.37% (203 nm).

Optical Path Length and Photon Utilization

The gas-phase branching ratio for N_2O absorption is independent of optical path length. Define the absorption coefficient of species (i) as

$$\alpha_i(\lambda) = \sigma_i(\lambda)A_i, \qquad \alpha_{\text{tot}}(\lambda) = \sum_i \alpha_i(\lambda) \tag{S9}$$

where σ_i and A_i are the absorption cross section and number concentration of species i, respectively. The fraction of gas-absorbed photons assigned to N_2O is

$$\gamma_{\text{N}_2\text{O}}(\lambda) = \frac{\alpha_{\text{N}_2\text{O}}(\lambda)}{\alpha_{\text{tot}}(\lambda)} \tag{S10}$$

Increasing path length does not change this branching ratio; it increases the fraction of emitted photons absorbed by a gas before reaching a reactor surface. The relevant length scale is therefore the total gas-phase extinction length. The 1/e extinction length and the path length required to absorb a fraction p of photons are

$$L_{\text{ext}}(\lambda) = \frac{1}{\alpha_{\text{tot}}(\lambda)}, \qquad L_p(\lambda) = -\ln(1-p)L_{\text{ext}}(\lambda) \tag{S11}$$

For the tropospheric composition used above (25 °C, 1 atm, 1.1 mol% water vapor, and 10 ppb O_3), summing the absorption coefficients gives the extinction lengths in Table S6. The values depend on total absorption by the gas mixture rather than N_2O alone. O_2 accounts for 78% of total absorption at 201 nm and 95% at 207 nm, while H_2O accounts for 21% at 201 nm. In contrast, the N_2O-only 1/e extinction lengths are 33.2 km at 201 nm and 86.1 km at 207 nm.

Table S6. Calculated N_2O absorption yield and gas-phase extinction lengths under representative tropospheric conditions

Wavelength (nm)	Absorption yield (%)	Total $L_{1/e}$ (m)	$L_{90\%}$ (m)	$L_{99\%}$ (m)	N_2O-only $L_{1/e}$ (km)	O_2 share (%)

193	0.135	18	41	83	13.3	26
200	0.477	139	319	639	29.1	72
201	0.478	159	366	731	33.2	78
207	0.299	258	594	1,187	86.1	95
222	0.022	453	1,044	2,088	2,022	97

The AQY_{N2O} values calculated above should consequently be interpreted as upper limits that assume all emitted photons are absorbed by gases. If η_{abs} is the fraction of emitted photons absorbed by gases rather than lost at reactor surfaces, then the effective yield per unit of electrical energy is $\eta_{rad}\gamma_{N2O}\eta_{abs}$, and the actual SEC is SEC_{ideal} / η_{abs}. SEC will therefore increase to the degree that internal reflectance is imperfect or the available optical path is short.

Reflective internal surfaces can increase the effective optical path, but achieving high deep-UV reflectance over large reactor areas is an additional materials and reactor-engineering challenge. Rather than assigning an unconstrained capital-cost increment, this analysis identifies a further research need: optical surfaces and reactor geometries that provide high photon utilization at 193–222 nm. In practice, the long extinction lengths favor large-volume reactor designs with long optical paths.

Table S7. Photolysis products

Gas	Absorption yield at 201 nm	Photolysis products
O_3	0.13%	O_2 + O. At 201 nm, 59% of the single O are in the excited $O(^1D)$ state with the remainder in the ground state $O(^3P)$.[21]
O_2	78.35%	O + O. At 201 nm, all O is in the ground state $O(^3P)$.[21] Below 175 nm, 50% of O is formed as $O(^1D)$.[21]
H_2O	21.02%	At 185 nm, >99% quantum yield to OH + H, extrapolated to 201 nm.[21]
N_2O	0.48%	N_2 + $O(^1D)$ at 201 nm.[21]
CO_2	0.02%	CO + $O(^3P)$ ($O(^1D)$ only formed below 170 nm).[25]

Combining these reactions yields the following products for 1 mole of photons reacting with air: 0.0056 $O(^1D)$, 1.57 $O(^3P)$, 0.21 OH, 0.21 H, 0.0013 O_2, 0.0048 N_2, and 0.0002 CO. Several of these products are reactive species, indicating that significant subsequent reactions will occur in

addition to the original N_2O photolysis. While a complete analysis is beyond the scope of this work, two main outcomes with feasibility implications are addressed.

First, the quantum yield of $O(^1D)$ is 0.56%. While these radicals may react with N_2O, increasing the effective removal efficiency, they also readily react with other species. Table S8 contains the rate coefficients of these reactions (k_i) and the relative reaction rates (k_iC_i, where C_i is the concentration of the other species). Given a relative reaction rate with N_2O of 1.25×10^{-6}, the overall yield of $O(^1D)$ generation to N_2O decomposition is 7.03×10^{-9}, negligible when compared to direct photolysis. This result also shows that any other process for generating $O(^1D)$ radicals will have a negligible effectiveness for N_2O destruction.

Table S8. Rate coefficients for $O(^1D)$ reactions

Reactant	k_i Rate coefficient at 298 K (cm^3 molecule^{-1} s^{-1})	k_iC_i (s^{-1})	Relative rate
N_2	3.0×10^{-11}	5.77×10^{8}	0.69
O_2	3.95×10^{-11}	2.03×10^{8}	0.24
H_2O	2.0×10^{-10}	5.62×10^{7}	0.07
CO_2	1.1×10^{-10}	1.14×10^{6}	1.36×10^{-3}
N_2O	1.27×10^{-10}	1.05×10^{3}	1.25×10^{-6}

Second, a large number of $O(^3P)$ are formed (both directly and via the deactivation of $O(^1D)$ in collisions with O_2, N_2, H_2O, and CO_2). The primary sink for $O(^3P)$ is reaction with O_2 to form O_3. With a quantum yield of 1.57 for $O(^3P)$, 201-nm photolysis will generate a significant amount of O_3. Practically, this will lead to a rapid increase in the O_3 concentration, increasing the absorption yield of O_3 and decreasing the absorption yield for N_2O. Given this, the N_2O absorption yields determined here should be considered as instantaneous initial values. Ozone is also a harmful air pollutant when produced at ground level, requiring an additional ozone treatment stage for a closed-system process.

Light sources: While there are several types of UV light sources available (Table S9), they are limited in their radiant efficiency (the ratio of light output to electrical input) and operate only at specific wavelengths. Operation away from the 201 nm peak will result in a lower quantum yield: only two light sources, ArF* and KrBr* excimer lamps, can produce wavelengths which provide an absorption yield of >0.01% at 50% RH. With unit quantum yield, AQY_{N2O} equals the absorption yield, and the yield per unit of electrical energy is the product of the radiant efficiency and the absorption yield for N_2O: $\eta_{rad} \cdot \gamma_{N2O}$.

Table S9. UV light sources and corresponding yield per unit of electrical energy

Source	Wavelength (nm)	η_{rad} (%)	Absorption yield (γ_{N2O}) at 0 and 50% RH	Yield per unit of electrical energy at 0% RH[a]
Light emitting diode (LED)	>250	<5% in 250–300 nm range.[26]	<0.01%	<0.0005%
Low pressure mercury vapor (two main UV peaks with minimal emission in 200-nm region).[27,28]	254	40%	<0.01%	< 0.004%
	185	11%[29]	0.18% | 0.01%	0.02%
Excimer sources	193 (ArF*)	4.4% (lamp)[30] 10% (laser)[31]	0.52% | 0.14%	0.052%
	207 (KrBr*)	5–18%[32]	0.31% | 0.30%	0.056%
	172 (Xe_2*)	60%[32]	<0.01%	< 0.006%

[a]Yields at 0% RH should be interpreted as best-case ceilings.

The highest yield per unit of electrical energy achievable with these existing light sources is ~0.05%. Based on the closed-system cost model (Section 3.1), these processes are likely climate-beneficial but will be cost-prohibitive. Yet, an idealized light source with 100% radiant efficiency at 201 nm would require a factor of ten less electricity, SEC ≈ 0.5 Wh m^{-3}, well below the full system DAC reference point (Figure 3).

S6. Photocatalytic Reactors

Photocatalysts are typically semiconductors with an energy difference between valence and conduction bands called a *bandgap*. Semiconductors normally behave as insulators, with all electrons stuck in the low-energy valence bands. A photon can excite an electron into the conduction band, leaving behind a *hole*, if it has energy greater than the *bandgap*. The electron and *hole* can each react with other molecules adsorbed to the surface of the photocatalyst, including N_2O.

Photocatalytic AQY_{N2O} is influenced by both intrinsic material properties and extrinsic conditions: (1) Bandgap: Traditional photocatalysts like titanium dioxide (TiO_2) have a bandgap in the UV range,[33] yet the majority of the solar spectrum is in the visible range[34] and artificial lights are generally more expensive and less efficient in the UV range.[35] Reducing the bandgap enables the use of visible wavelengths, but also makes it easier for the excited electron to recombine with its hole before reacting with N_2O. (2) Surface adsorption: An N_2O molecule must adsorb onto the catalyst surface before reacting. The material and surface preparation determine the

number and selectivity of active sites.[36] (3) Concentration: The higher the concentration of N_2O, or any species, the greater the likelihood that a molecule collides with and sticks to an active site. This relationship is thought to be linear at the low concentration of ambient N_2O, as observed in analogous systems,[37] based on a Langmuir–Hinshelwood mechanism.

Apparent quantum yield:

To my knowledge there are no controlled studies of N_2O photocatalysis at ambient atmospheric conditions that report sufficient information to determine the AQY for the studied catalyst. Further, AQY_{N2O} depends on several factors related to not only the catalyst material itself but also the testing conditions, making extrapolation from other conditions of limited use. This relationship can be modeled by the following equation for a simplified photocatalytic reaction[38]:

$$AQY_{N2O} = \frac{\phi\, k\, \theta\, C_{R,0}}{\phi L_p^a + k_r + k\, \theta\, C_{R,0}} \qquad \text{(S12)}$$

where the following variables are solely properties of the catalyst:

- ϕ: The yield of reactive charges per incident photon (mol site mol photon^{-1})
- $C_{R,0}$: Concentration of reactive sites (mol site g_{cat}^{-1})
- k_r: Rate of charge recombination (mol site g_{cat}^{-1} s^{-1})

Of the environment:

- L^a_p: Incident light intensity (mol photon g_{cat}^{-1} s^{-1})

And, of the catalyst–N_2O interaction:

- k: kinetic rate constant for N_2O destruction (s^{-1})
- θ: Surface Coverage (unitless)

At atmospheric conditions, the low concentration of N_2O will reduce the surface coverage, the fraction of reactive sites which have an adsorbed N_2O. It is typically modeled as[38]:

$$\theta = \frac{K_{ads}\,[C]}{1 + K_{ads}\,[C]} \qquad \text{(S13)}$$

where $[C]$ is the concentration of N_2O and K_{ads} is an adsorption constant. At low concentration ($K_{ads}\,[C] \ll 1$), this can be approximated by $\theta = K_{ads}\,[C]$, which indicates that the surface coverage is both linearly proportional to the concentration and is itself $\ll 1$.

For an optimal catalyst, assume $\phi = 1$ and $k_r = 0$:

$$AQY_{N2O} \approx \frac{k\,\theta\,C_{R,0}}{L_p^a + k\,\theta\,C_{R,0}} \quad \text{(S14)}$$

Thus, AQY_{N2O} is maximized under low-light conditions, i.e., $L^a{}_p \ll k\,\theta\,C_{R,0}$ (to prevent supplying photons in excess of the reaction rate). Thus, for an optimal catalyst under illumination to maximize AQY_{N2O}:

$$AQY_{N2O} \propto k\,\theta\,C_{R,0} \propto k\,C_{R,0}\,K_{ads}[C] \quad \text{(S15)}$$

The maximum AQY_{N2O} is therefore proportional to the surface coverage, which itself is proportional to N_2O concentration at low concentration. To my knowledge, there are no experimental reports of AQY_{N2O} over a range of concentrations, although one study does report a decrease in N_2O conversion when going from 1,000 to 100 ppm inlet concentration.[39] While the photocatalytic destruction of N_2O and CH_4 are distinct processes, similar photocatalytic materials have been studied for both, and, in the absence of experimental data on atmospheric N_2O, the more widely studied CH_4 reaction provides a reference. A recent study by Kessler et al. of CH_4 oxidation at concentrations from 2–5,000 ppm confirms a linear relationship below ~1,000 ppm for several catalyst materials, and reports AQY_{CH4} of 0.0001–0.01% at 2 ppm.[40] The upper end of this range is still one to two orders of magnitude below Randall et al.'s AQY_{CH4} target.[41]

Equations S12–S15 describe a simplified single-species kinetic model. At low N_2O concentration, the model predicts low fractional N_2O surface coverage and an approximately concentration-proportional maximum AQY. In humid air, however, surface state, charge-carrier kinetics, and competing species can alter the proportionality; this behavior has not been directly established for N_2O.

Adsorption behavior alone does not provide a basis for ranking AQY_{N2O} against NO_x or CH_4. Computed low-coverage adsorption energies for N_2O and NO on the same anatase (101) surface are similar, while CH_4 is also weakly physisorbed and its reported adsorption energy depends substantially on TiO_2 facet and surface state.[42,43] Under environmentally relevant humidity, surface state and charge-carrier kinetics can be more important than gas adsorption strength.[40] More importantly, the reactions differ: N_2O decomposition is reductive, whereas NO_x and CH_4 removal are oxidative processes.[44,45] Thus, their reported adsorption behavior and AQYs should not be used to infer a quantitative AQY ordering for N_2O.

Photocatalytic N_2O removal in air requires a reduction to proceed in the presence of O_2, which is generally held to scavenge photogenerated electrons,[46] while calculations on TiO_2 show that N_2O dissociates only with a photoexcited electron or at an oxygen vacancy.[44] The few N_2O photocatalysis tests with O_2 present found no suppression by O_2, but they used 10–1,000 ppm N_2O and lacked the photon and product accounting needed to detect a diversion of electrons to O_2,[39,47,48] so whether O_2 limits the reaction at ambient concentration is unknown. An active site that adsorbs N_2O selectively and is passivated against O_2 would address this; the same principle was recently shown for the reduction of NO to N_2, through an adsorbed N_2O

intermediate, in 20% O_2,[49] making site design for O_2 tolerance a large and largely unexplored research opportunity.

Kessler et al. provide a useful benchmark for the type of characterization needed rather than for the expected magnitude of AQY_{N2O}. Their controlled CH_4 experiments resolve AQY across 2–5,000 ppm and show strong humidity dependence.[40] Equivalent N_2O studies should measure AQY across a concentration series spanning atmospheric conditions, at multiple humidities and irradiances, and with product identification to distinguish sustained N_2O decomposition from other nitrogen transformations.

Bueno-Alejo et al. measured the N_2O flux in cropped fields with and without catalyst application. The majority of measurements show net production of N_2O from the soil, with treated-field fluxes lower than control fluxes by an average of ~0.1 μg m^{-2} s^{-1}.[50] Extrapolating these flux measurements to an annual per-field removal is fraught with confounding effects: precipitation, temperature, time since nitrogen fertilizer application and spatial microclimate variability can strongly influence the ecosystem's baseline N_2O production, while cloud cover and catalyst application frequency and durability will influence the removal rate.

Designing a small-scale field trial with an adequate baseline is inherently difficult, and these data support the reported reduction only weakly. Each crop had one treated field and one adjacent control field, sampled simultaneously or immediately afterward, with 5–9 air samples per field per visit at two heights. There was no pre-treatment baseline, no irradiance record, and no measurement in darkness, and fluxes were estimated by the flux-gradient method from daily-average wind speeds at the nearest weather station.[50] Across the eight visits with paired control data, the treated-minus-control flux difference averages 0.12 ± 0.07 μg m^{-2} s^{-1} (1 SE; one-sample *t*-test, $p \approx 0.14$), and on two of those visits the treated field emitted more than the control. The statistical significance of the reported reduction, and its attribution to photocatalysis, are therefore low.

Assuming the measurements were made in full sun and counting incident photons below 365 nm (the AM1.5G basis of Section S9.3), this flux corresponds to an instantaneous AQY_{N2O} = 0.0036%, not accounting for the incomplete areal coverage of the catalyst. Because the irradiance during sampling was not reported, full sun gives the lowest plausible value. The value can be divided by the surface coverage of the catalyst to estimate the AQY_{N2O} of the catalyst itself. Photos of sprayed crop leaves indicate a coverage in the range of 10–20%, resulting in AQY_{N2O} = 0.02–0.04%.[50] However, based on the application protocol and catalyst particle size and loading data reported in a previous paper,[39] I estimate a lower bound for surface coverage at only 1.5%, indicating a maximum potential AQY_{N2O} of 0.24%.

To annualize this flux, it is taken as the full-sun rate and applied for the full-sun-equivalent hours of the season: the solar capacity factor of 22.9% (Section S9.3) corresponds to 5.5 full-sun hours per day. Over a 90-day growing season, 0.1 μg m^{-2} s^{-1} then gives ~0.5 tCO_2e ha^{-1}. This assumes that AQY_{N2O} does not change with irradiance. If the rate instead saturates at lower

light, the same flux would persist through more of the day, raising the seasonal removal by up to a factor of about two if it held through all daylight hours. The main text reports 0.5–2 $tCO_2e\ ha^{-1}$, allowing for uncertainty in the reported mean field flux.

Cost of lighting:

With AQY_{N2O} unknown, previous work has levelized the cost of the lighting system as a function of the lighting energy. Randall et al. estimate a lower-bound capital cost of \$0.074 kWh^{-1} for LED modules (not including other mounting hardware or power electronics).[41] In 2025 USD (Table S2), this is \$0.078 kWh^{-1}. Their analysis assumes each LED costs \$5.29, has an electrical power of 1.6 W, and an operating lifetime of 50,000 h.

Alternatively, the capital cost of the LEDs can be amortized across the airflow to produce a cost in units of \$ cfm^{-1} for use in the system cost model. For a flow of 1 cfm, the air flow rate is:

$$\frac{1\ ft^3}{min} \times \frac{1\ m^3}{35.3147\ ft^3} \times \frac{1\ min}{60\ s} = 0.000472\ m^3\ s^{-1} \tag{S16}$$

The flow of N_2O can be calculated from the concentration:

$$0.000472\ m^3 s^{-1} \times \frac{40.92\ mol\ air}{1\ m^3} \times \frac{0.339\ ppm\ N_2O}{1 \times 10^6\ ppm\ air} = 6.54 \times 10^{-9}\ mol\ N_2O\ s^{-1} \tag{S17}$$

At the midpoint of the estimated range, AQY_{N2O} = 0.03%. With an LED radiant efficiency of 60% and a wavelength of 365 nm, the required electrical power is:

$$\frac{6.54 \times 10^{-9}\ mol\ N_2O}{1\ s} \times \frac{1\ mol\ photons}{0.0003\ mol\ N_2O} \times \frac{3.28 \times 10^5\ J_{light}}{mol\ photon} \times \frac{1\ J_{elec}}{0.60\ J_{photon}} = 11.9\ W_{elec} \tag{S18}$$

Assuming the same LED specifications of 1.6 W_{elec} and \$5.29 per module, the illumination requirement is 7.45 LEDs cfm^{-1} or \$39.41 cfm^{-1}. Across the 0.02–0.04% range used in the main text, this inverse scaling gives 5.59–11.17 LEDs cfm^{-1} and LED-module CAPEX of \$29.56–59.11 cfm^{-1}.

As a second reference for the cost model, Randall et al. developed a detailed techno-economic model for a photocatalytic reactor.[41] They estimate an air-handling cost of \$63–6,300 tCO_2e^{-1} gross removal depending upon the per-area reaction rate. While this reaction rate is not determined by AQY_{N2O} alone, it is the product of AQY_{N2O} and light intensity, thus a higher AQY_{N2O} is beneficial for increasing the per-area reaction rate and reducing the air-handling cost. This cost is ~80% air contactor hardware and ~20% air handling energy. Their estimated lighting cost is inversely proportional to AQY_{N2O}; for example, the estimated gross-removal cost increases from \$160 to \$1,600 tCO_2e^{-1} as AQY_{N2O} decreases from 1% to 0.1%. These values are sensitivity benchmarks rather than demonstrated performance; achieving modeled lighting costs of order \$100–200 tCO_2e^{-1} therefore requires an apparent quantum yield of order 1%.

S7. Aqueous Solubility of Greenhouse Gases

For greenhouse gas removal approaches which involve an aqueous step, the solubility of the greenhouse gas in water influences the overall rate. The saturation concentration is the product of the solubility constant and the partial pressure of the gas phase that is in equilibrium with the liquid phase. In a closed-system process involving an air–liquid contactor and handling of that liquid stream, a higher saturation concentration reduces the amount of liquid that must be processed per unit removal. The rate of aqueous phase reactions, e.g., biological N_2O consumption via denitrification or fixation pathways, is limited by the aqueous concentration, either kinetically or through diffusion-based mass-transfer limits. N_2O has a significantly higher solubility than CH_4 in pure water (Table S10). Thus, despite N_2O having a lower atmospheric concentration than CH_4, its aqueous equilibrium concentration is three times higher. Accounting for their respective molar masses and GWP_{100} values, N_2O has an 81 times higher CO_2-equivalent aqueous equilibrium concentration than CH_4.

Table S10. Aqueous equilibrium concentration for greenhouse gases

	Atmospheric concentration		Solubility (298 K)[21]	Aqueous equilibrium concentration	
Gas	(ppm)	(ppm CO_2e)	(M atm^{-1})	(μM)	(μM CO_2e)
CO_2	428	428	3.44×10^{-2}	14.72	14.72
CH_4	1.94	19.7	1.41×10^{-3}	2.74×10^{-3}	2.76×10^{-2}
N_2O	0.339	92.5	2.42×10^{-2}	8.20×10^{-3}	2.24
N_2	7.81×10^5		6.52×10^{-4}	509.2	
O_2	2.09×10^5		1.27×10^{-3}	265.4	

S8. Bioreactor References

Hood et al. report on the operation of a rudimentary small-scale biofilter on swine barn pit ventilation exhaust in 2011.[51] Table 8 in Hood et al. reports season-averaged inlet concentrations of N_2O ranging from 0.78 ± 0.079 to 1.00 ± 0.09 mg m^{-3}. Assuming an air molar density of 40.92 mol m^{-3} and N_2O molar mass of 44.01 g mol^{-1}, this range is 0.43 to 0.56 ppm. Table 9 reports season-averaged removal efficiency for N_2O ranging from 14.0 ± 4.0% to 18.3 ± 7.6%.

Table 8 also reports net CO_2 production in one season from 1,038.6 ± 111.8 to 1,103.4 ± 209.9 mg m^{-3} (likely not statistically significant) and net consumption in the other two seasons. An increase in CO_2 concentration of 100 mg m^{-3} corresponds to 55.5 ppm assuming an air molar density of 40.92 mol m^{-3} and CO_2 molar mass of 44.01 g mol^{-1}, indicating an insignificant change in the O_2 level.

Hood et al. report a materials cost of \$1,930 and an airflow rate of 0.52 m^3 s^{-1}, providing a minimum bound of CAPEX = \$1.76 cfm^{-1}. They report a cost of consumables of \$0.46 d^{-1}, of which two-thirds is electricity and one third is water. Electricity use is also reported as 2.1 kWh d^{-1}, which is equivalent to 0.047 Wh m^{-3}. They also report a maintenance time of approximately one hour per week.

Although N_2OR requires anoxic conditions at the enzyme, N_2O-reducing bacteria show a varying sensitivity to ambient O_2: those with the lowest sensitivity show higher O_2 respiration rates, suggesting a protective mechanism to form anoxic conditions within the cell.[52]

This cost estimate uses the reported materials cost as the capital cost, inflation-adjusted to 2025 (Table S2), giving \$1.76 cfm^{-1}. Water use and labor are omitted from the operating cost, which just includes the electricity at 0.047 Wh m^{-3}. Both of these values are scaled by a factor of 6 to reflect the low N_2O removal efficiency: at 14–18.3% removal, roughly six times as much air must be processed per unit of N_2O as would need to be at 100% removal. Thus, the final estimates for effective cost model inputs are CAPEX = \$10.56 cfm^{-1} and SEC = 0.28 Wh m^{-3}.

Pressure drop does not necessarily increase with empty-bed residence time. In the viscous-flow limit, Darcy's law gives $\Delta P = \mu L u / k$, while $\tau_{EBRT} = L/u$, where μ is gas viscosity, L is bed depth, u is superficial velocity, and k is bed permeability. At fixed bed depth, increasing residence time by lowering velocity therefore gives $\Delta P = \mu L^2/(k\tau_{EBRT})$, reducing pressure drop while increasing the required frontal area; increasing residence time by deepening the bed at fixed velocity instead increases pressure drop. Biofilters can exploit this footprint–energy trade-off because they can use shallow, wide geometries without the heat-loss constraints of high-temperature reactors. For the Hood et al. system described above, the 0.30 m bed, superficial velocity of 0.04 m s^{-1}, and 7.6 s empty-bed residence time were associated with an average fan pressure drop of 125 Pa. At the reported flow of 0.52 m^3 s^{-1}, this pressure drop corresponds to 1.56 kWh d^{-1} of fluid work, consistent with the reported total electricity use of 2.1 kWh d^{-1} at an implied overall fan efficiency of approximately 74%.

Multistage biological systems have also been demonstrated for higher-concentration N_2O emissions from wastewater treatment plants. Han et al. report that a 250–500 L min^{-1} pilot system would not be cost-effective for gross N_2O removal at its average operating concentration of 17 ppm at a credit value of €74 tCO_2e^{-1}.[53] Scaling the reported cost inversely with N_2O concentration to atmospheric conditions gives a gross removal cost above €3,710 tCO_2e^{-1}. In 2025 USD (Table S2), this is >\$4,370 tCO_2e^{-1}. However, this gross removal cost is not decision-relevant once emissions from O_2 depletion are included. For one mole of air, depletion of 10% of the 20.95 mol% O_2 consumes 0.02095 mol O_2. Assuming oxidation of organic carbon produces one mole of CO_2 per mole of O_2 consumed, this generates 0.02095 mol CO_2. Complete removal of atmospheric N_2O (339×10^{-9} mol N_2O mol^{-1} air) is equivalent to $339 \times 10^{-9} \times 273$ mol CO_2e because N_2O and CO_2 have the same molar mass. The ratio is therefore $0.02095/(339 \times 10^{-9} \times 273) = 226$, so CO_2 production is 226 times greater than the N_2O removed on a CO_2e basis.

This calculation neglects other energy and material emissions and therefore remains a lower bound.

S9. Open-System Cost Model

S9.1. Cost Model

The techno-economic model estimates the gross cost of atmospheric greenhouse gas removal in units of US dollars per metric ton of CO_2 equivalent ($ tCO_2e^{-1}). The calculation is based on normalizing the intervention cost and the rate of N_2O removal to the surface area of the open-system intervention (i.e., m^2 of photocatalytic paint or ha of ecosystem intervention).

This model is intentionally simplified, incorporating just the annualized per-area cost of the intervention and the rate of N_2O destruction. It does not account for the emissions associated with producing, deploying, or maintaining the intervention which may reduce the net removal. A low-fidelity model is sufficient for the purpose of this assessment and the precise data that would be needed for a more complete model does not exist. Given that additional factors would likely both increase the total cost and decrease the net climate benefit, this model represents a lower bound on the net cost of removal. The gross cost of removal is defined as:

$$C_{open} = \frac{C_A}{R_{CO2e}} \tag{S19}$$

where C_A is the annualized per-area intervention cost ($ m^{-2} y^{-1}) and R_{CO2e} is the annual average removal rate of N_2O (tCO_2e m^{-2} y^{-1}). The N_2O flux is converted to CO_2e assuming GWP = 273 (100-year time horizon).

For a given intervention, all costs can be scaled by the intervention area and annualized to include in C_A. However, C_A may be misleading, especially when comparing interventions with widely different scale or duration, if a significant portion of costs are fixed with respect to the intervention area or project duration. Similarly, R_{CO2e} can be calculated to include all of the emissions associated with an intervention, including the embodied emissions of capital equipment, ongoing emissions from energy use, and changes to other greenhouse gas fluxes.

For approaches where the cost is a combination of upfront or periodic and ongoing annual costs, the former can be annualized by multiplying by a capital recovery factor (CRF, Table S3, eq S3) based on the relevant period, *N* (years), and the discount rate, *r* (%), as described in Section S2.

S9.2. Reaction Rate Mass-Transfer Limit

The upper limit of reaction rate is determined by the mass-transfer rate of N_2O from the atmosphere to the surface. As a typical case, an intervention area of characteristic length *L*

subject to a horizontal wind velocity v_{wind} is considered. The mass-transfer rate (mol m^{-2} s^{-1}) for this forced-convection configuration is:

$$m = \frac{Sh_L \cdot D_{N2O,air} \cdot \Delta C}{L} \quad \text{(S20)}$$

where Sh_L is the dimensionless Sherwood number (analogous to the Nusselt number for heat transfer), $D_{N2O,air}$ is the diffusivity of N_2O in air (0.143 cm^2 s^{-1} at 20 °C and 1 atm),[54] and ΔC is the difference in concentration between the surface and the bulk atmosphere (mol m^{-3}). To find the upper limit of m, ΔC is maximized by assuming the reaction rate at the surface is sufficiently high that the surface concentration of N_2O is zero and ΔC is equal to the bulk concentration.

Given uncertainty about the deployment conditions, several potentially applicable empirical correlations for Sh_L are considered. First, a model for laminar flow over a flat plate[18]:

$$Sh_L = 0.664\, Re^{1/2} Sc^{1/3} \quad \text{(S21)}$$

$$Re = \frac{v_{wind}\, L}{\nu_{air}}, \quad Sc = \frac{\nu_{air}}{D_{N2O,air}} \quad \text{(S22)}$$

where Re is the Reynolds number ($Re < 5 \times 10^5$ for laminar), Sc is the Schmidt number, and ν_{air} is the kinematic viscosity (m^2 s^{-1}). The laminar constraint makes this correlation only applicable for small areas and low wind speeds, e.g., m = 0.62 μg m^{-2} s^{-1} for v_{wind} = 1 m s^{-1} and L = 6 m. The boundary layer becomes turbulent for higher v_{wind} or L, with the resulting mixing increasing mass transfer. Any real outdoor surface is unlikely to be exposed to such idealized conditions. Studies of outdoor surfaces have shown that turbulence is almost always already present from natural convection and rough or unaligned surfaces, suggesting the critical transition to turbulence is as low as Re = 1,000.[55] Second, a Sh_L correlation applicable for building roofs under turbulent conditions is considered[55]:

$$Sh_L = R_f\, 0.037\, Re^{4/5} Sc^{1/3} \quad \text{(S23)}$$

Under the same conditions, the turbulent correlation outputs 2.76 μg m^{-2} s^{-1}. Increasing the characteristic length drastically to L = 1,000 m, representative of a large-scale ground-level intervention, only decreases m to 0.99 μg m^{-2} s^{-1}. Further, it should be noted that reports of average wind speed are at some height above the surface (e.g., 10, 30 m or more)[56] and thus may not be directly applicable to this analysis. Yet, even at a height of 10 m, the average wind speed is above 1 m s^{-1} in most regions.[56] Here, the value m = 1 μg m^{-2} s^{-1} is used as a representative threshold for bulk mass transfer, indicating that the bulk mass transfer (and thus local air flow patterns and intervention geometry) can be neglected if other rate-limiting steps exist for lower values of m.

Sustained continuously, m = 1 μg m^{-2} s^{-1} corresponds to an annual removal of ~90 tCO_2e ha^{-1} y^{-1}. The field flux of Section S6, ~0.1 μg m^{-2} s^{-1} from a field 10–20% covered by catalyst, is an

order of magnitude below this limit. A surface whose area was entirely active catalyst would therefore be within a factor of two of the limit.

As an example of the use of forced airflow to increase the mass-transfer rate, the roof of a truck with L = 10 m and v_{wind} = 20 m s^{-1} has a mass-transfer rate limit of m = 28 μg m^{-2} s^{-1}.

S9.3. Solar AQY

For a sunlight-powered photocatalyst, the full-sun AQY_{N2O} is converted to an annual-average N_2O removal rate (mol N_2O m^{-2} s^{-1}) by multiplying it by the annual average photon flux from sunlight (mol photon m^{-2} s^{-1}), assuming the removal rate is proportional to irradiance (eq S24). The AM1.5 Global Spectrum is assumed for incoming solar flux, which has an integrated power of 1,000 W m^{-2} and was designed to represent relevant conditions for terrestrial flat-plate solar modules.[57]

Given the intended use of this metric for photocatalytic removal, a cutoff wavelength of 365 nm is used. The AM1.5G spectral irradiance is converted from units of W m^{-2} nm^{-1} to mol photons s^{-1} m^{-2} nm^{-1} and integrated for wavelengths below the cutoff wavelength, resulting in a total UV photon flux of 6.33×10^{-5} mol m^{-2} s^{-1}.[57] Modeling of solar irradiance in North America indicates an annualized average integrated power of 125 (northern Canada) to 270 (southwest United States and Mexico) W m^{-2},[58] indicating a capacity factor of 12.5–27%. A capacity factor of 22.9%, corresponding to an average daily insolation of 5.5 kWh m^{-2}, is used here:

$$R_{N_2O} = AQY_{N_2O} \times 6.33 \times 10^{-5} \times 22.9\% \quad \text{(S24)}$$

where R_{N2O} is the annual-average removal rate (mol N_2O m^{-2} s^{-1}), AQY_{N2O} is the full-sun instantaneous value, 6.33×10^{-5} mol m^{-2} s^{-1} is the full-sun photon flux below 365 nm, and 22.9% is the solar capacity factor. Converted with the molar mass of N_2O, GWP_{100}, and the seconds in a year, each 0.01% of AQY_{N2O} on a surface whose area is entirely active catalyst corresponds to ~5.5 tCO_2e ha^{-1} y^{-1}.

Counting solar photons below the 365 nm cutoff when computing AQY_{N2O} allows direct comparison between laboratory AQY_{N2O} measurements with pure 365-nm illumination and real-world performance under sunlight. For a photocatalyst with a higher cutoff wavelength, i.e., a lower bandgap, the usable solar irradiance increases significantly. The usable photon flux is doubled when integrated to 395 nm and increased fourfold when extended to 427 nm.

S9.4. Updraft Tower Analysis

De Richter et al. estimate that a solar updraft tower of sufficient size to generate 200 MW of electricity (using a turbine within the buoyancy-driven flow) has a collector area of 38 km^2 and processes an air volume containing 3,900 t N_2O per year during daylight hours.[59] This is equivalent to 280 tCO_2e ha^{-1} y^{-1}. Complete greenhouse gas removal, as they propose, would

require complete area coverage of a catalyst with AQY_{N2O} = 0.5% (eq S24) and similar performance for CH_4 and CFCs.

For a static surface, the field flux of Section S6, ~0.1 μg m^{-2} s^{-1} from a field 10–20% covered by catalyst, is an order of magnitude below the stationary-surface mass-transfer limit (Section S9.2). A coating whose area was entirely active catalyst would be within a factor of two of that limit, so added airflow, as on vehicle exteriors or in updraft towers, would benefit it only if AQY_{N2O} also improved. At the 10–20% coverage of powder dispersal, by contrast, there is headroom for a tenfold or greater increase in AQY_{N2O} before mass transfer limits removal.

S9.5. Photocatalytic Surface Coating Cost Sensitivity

All cost values in this subsection are reported in 2025 United States dollars following the currency and inflation-adjustment procedure in Section S1.2. Upfront coating costs are annualized using the default 10-year economic lifetime and 8% discount rate, corresponding to a capital recovery factor of 0.149: C_A = 10,000 (CRF C_{cap} + C_{clean}), where C_{cap} is installed coating cost ($ m^{-2}) and C_{clean} is annual cleaning cost ($ m^{-2} y^{-1}). The lower-cost case uses the Telangana government Schedule of Rates for installed cool-roof coating, $3.84 m^{-2}. The upper-cost case uses the installed cost in the United States commercial-roof model of Randall et al., $18.8 m^{-2}. Both cases use the median maintenance cost observed by Sproul et al., $0.27 m^{-2} y^{-1}. This is substantially lower than the washing assumption in Randall et al. ($2.1 m^{-2} y^{-1}) and should therefore be regarded as an optimistic maintenance benchmark for a photocatalytic coating.[41,60,61]

These assumptions yield C_A = $8,400 ha^{-1} y^{-1} for the lower-cost case and $30,700 ha^{-1} y^{-1} for the upper-cost case. At the upper estimate of the field catalyst's AQY_{N2O}, 0.04%, a coating whose area is entirely active catalyst would remove ~22 tCO_2e ha^{-1} y^{-1} (eq S24), giving C_{open} = $380–1,400 tCO_2e^{-1}. Such a coating would be within a factor of two of the mass-transfer limit (Section S9.4), so mass transfer would also slow removal; this rate is an upper bound, and these costs a lower bound.

Cost assumptions therefore matter a great deal, yet currently reported reaction rates remain insufficient even under the lower-cost case. That case reaches $200 tCO_2e^{-1} only if R_{CO2e} increases to approximately 42 tCO_2e ha^{-1} y^{-1}, corresponding to $AQY_{N2O} \approx 0.08\%$, roughly twice the upper estimate, or more, because a coating whose area is entirely active catalyst would be limited by the mass-transfer limit near midday. The upper-cost case cannot reach $200 tCO_2e^{-1} even at the stationary-surface mass-transfer limit. These estimates are a sensitivity range, not a deployment-cost prediction, because photocatalyst loading, durability, field soiling, and cleaning frequency remain unmeasured for atmospheric N_2O removal.

S9.6. Reference Removal Rates

The cost model does not explicitly include the value of the land itself, and does not impose any penalty for the inefficient use of land area (so long as the intervention is proportionally low-cost). In some cases, N_2O removal interventions can be conducted on top of existing land use and it is appropriate to only consider this marginal cost. Yet, given a finite amount of suitable land, interventions with higher intensity should be prioritized, both within N_2O removal approaches and across other climate interventions. Thus, it is valuable to contextualize projected removal rates by comparing to other climate interventions.

- Mature tropical forests in Africa and the Amazon show an increase in live biomass of 0.66 t C ha^{-1} y^{-1}, equivalent to 2.4 tCO_2 ha^{-1} y^{-1}.[62]
- Reforestation via planted forests has a removal rate of 4.5 to 40.7 tCO_2 ha^{-1} y^{-1} for the first twenty years of growth across a range of species and climates.[63]
- Enhanced rock weathering studies show a wide range of removal rates in experimental studies, typically in the range 0.1 to 10 tCO_2 ha^{-1} y^{-1}.[64]
- A global modeling study indicates that large-scale deployment of BECCS (bioenergy with carbon capture and storage) could yield a removal rate of 11.8 tCO_2 ha^{-1} y^{-1} for bioenergy crops.[65]
- A study of enhanced rock weathering, compost, and biochar application to grassland in California found a net removal of 5.4 to 28.7 tCO_2e ha^{-1} y^{-1} across combinations of these treatments.[66]
- Increasing the stock of soil organic carbon on existing managed land could yield a removal rate of 2.2 to 4.8 tCO_2 ha^{-1} y^{-1}, although this effect will saturate after several decades.[67]

S10. The Nitrogen Cycle and Biological Mechanisms for N_2O Consumption

The exchange of nitrogen between inert dinitrogen (N_2) in the atmosphere and reactive nitrogen species in soils and waters is mediated entirely by microbial activity in natural ecosystems.[68] Nitrous oxide is a key component of the denitrification pathway, an anoxic respiratory process that evolved to replace the role of oxygen in environments where O_2 is limited or absent.[69,70] Because oxygen is the most energetically favorable terminal electron acceptor, microbes will preferentially respire O_2 whenever it is available; only when O_2 becomes depleted do they shift to the less efficient sequence in which nitrate (NO_3^-) and its intermediates (nitrite (NO_2^-), nitric oxide (NO), and N_2O) are reduced stepwise to N_2 to sustain respiration.

Thus, denitrification reactions, including both the production and consumption of N_2O, occur almost exclusively within oxygen-depleted microsites in soils or sediments. These microsites can only develop in pore spaces and aggregates that are largely isolated from the atmosphere, so the zones in which N_2O is most actively produced or consumed are inherently limited in atmospheric N_2O exchange as well.

Most terrestrial ecosystems are net sources of N_2O, but this net flux masks simultaneous gross production and gross consumption occurring in close proximity. Consumption occurs through two mechanistically distinct pathways. First, N_2O can be reduced to N_2 under anoxic conditions via N_2O reductase (N_2OR), the only known enzyme capable of catalyzing this reaction.[71,72] Second, N_2O can be fixed to ammonia (NH_3) or ammonium (NH_4^+) through the same enzymatic machinery that fixes dinitrogen (N_2). While this pathway was initially considered minor, a growing body of evidence shows substantial N_2O consumption in oxygen-rich environments such as shallow freshwater and ocean surface waters,[70] consistent with direct experimental observations of N_2O fixation via canonical N_2-fixation pathways.[73] Together, these findings highlight that N_2O consumption does not exclusively occur in anoxic microzones, even though these environments remain the primary sites of reduction to N_2.

Gross N_2O consumption typically follows the local N_2O availability, leading to an equilibrium with the local production rate. Consumption cannot exceed local production without requiring atmospheric drawdown, a process constrained by the low atmospheric concentration of N_2O and the restricted gas exchange characteristic of anoxic microsites where most consumption occurs. Conversely, increases in gross production raise pore-space N_2O concentrations, enabling higher rates of microbial N_2O respiration.

Isotopic approaches that independently quantify gross production and consumption corroborate this pattern: in net-source ecosystems with abundant mineral nitrogen, gross consumption scales strongly as a fraction of gross production, mediated by elevated pore N_2O concentration.[74] This relationship was observed to break down in ecosystems where gross production is too small to meaningfully elevate pore-space N_2O concentrations, and thus no longer exerts the dominant control on gross consumption.[74] There is little information on the distribution of gross fluxes in net-uptake ecosystems. In some cases, they may deviate from this relationship and have high rates of gross production and consumption, with the latter slightly higher. Or, they may have low gross production and even lower gross consumption. In either case, factors beyond gross production and pore-space N_2O concentration must also control gross consumption.

The range of observed N_2O and N fluxes in existing natural and managed ecosystems can bound the effect magnitude of potential interventions. Globally, croplands have an average net N_2O emission rate of 0–5 kg N_2O-N ha^{-1} y^{-1} (0–2.1 tCO_2e ha^{-1} y^{-1}).[75] Two reviews which focus only on field studies reporting net N_2O uptake, by far the minority, report net uptake of 0.1–1 kg N_2O ha^{-1} y^{-1} (0.03–0.27 tCO_2e ha^{-1} y^{-1}).[76,77] While there is general agreement that the scale of the existing terrestrial N_2O sink (i.e., the total uptake of land areas with net uptake, not the gross

uptake of land areas with otherwise positive net emissions) is negligible with respect to the atmospheric sink,[77] these rates may be an indicator of the attainable uptake that could be achieved through intervention. Direct measurement of gross N_2O uptake via the pool dilution technique in California cropland indicates an even larger flux up to 5.4 kg N_2O ha^{-1} y^{-1} (1.5 tCO_2e ha^{-1} y^{-1}), although this may be in part due to the greater N_2O availability from high gross emission rates. While gross production and consumption cannot be measured in the field, laboratory studies using the gas flow soil core (GFSC) technique indicate that these may be approximately an order of magnitude larger than gross emissions and uptake rates via pool-dilution technique[78] (like those reported by Yang et al.).[74]

S11. Potential Interventions for Ecosystem Uptake Enhancement

Trace metal amendments: Microbial metabolic pathways utilize trace metals like Cu, Zn, Fe, Mo, and Co as cofactors in key enzymes that control the emission of N_2O, CH_4, and CO_2. In situ metal concentrations in most environments are orders of magnitude below experimental metabolic optima, suggesting selective additions can modulate metabolic rates and thus greenhouse gas emissions.[79] In particular, copper is an essential cofactor for the denitrifying N_2OR enzyme.[79] One study of wetland sediment cores found trace Cu supplementation reduced net N_2O production fivefold over a 96-hour incubation.[80] The net greenhouse gas effect was a 78% reduction in produced CO_2e when accounting for small increases in CH_4 and CO_2 production. The primary challenges for trace-metal interventions include uncertain persistence in heterogeneous soils, the risk of metal toxicity (several earlier studies of higher Cu amendments produced divergent results attributed to Cu-induced toxicity),[80] the complexity of co-limitation with other nutrients, and the possibility of trade-offs, including elevated CH_4 or CO_2 emissions.

Microbial inoculation: Typical denitrifying bacteria have enzymes to perform several or all of the denitrifying reactions, thus their net effect on N_2O production depends on the relative expression of these enzymes. A subset, non-denitrifying N_2O-reducers, are only capable of reducing or respiring N_2O and are targets for inoculation interventions to introduce or increase their population.[81]

Several studies have sought to enrich non-denitrifying N_2O-reducing bacteria in the digestate waste of biogas reactors by incubation in anoxic, N_2O-rich conditions.[82,83] When the treated digestate was used as nitrogen-rich fertilizer, the post-application spike in N_2O emissions was reduced by up to 94%.[83] This effect is driven by accelerated N_2O respiration during transient anoxic periods following fertilization. During the period of highest emissions, the magnitude of reduction attributed to increased gross consumption peaked at a rate equivalent to 13,000 kg N_2O ha^{-1} y^{-1} (3,500 tCO_2e ha^{-1} y^{-1}), indicating a large capacity for consumption.[83] Reported Michaelis–Menten kinetics indicate a gross consumption rate of 101 kg N_2O ha^{-1} y^{-1} (28 tCO_2e ha^{-1} y^{-1}) when extrapolated to atmospheric N_2O concentration (K_m = 13.1 μM N_2O, average reported V_{max} = 0.64 fmol N_2O $cell^{-1}$ h^{-1}, treatment load of 6.6×10^{13} cell m^{-2}).[83] However,

respiration by these microbes is strongly suppressed by O_2, and the intervention has little activity during periods of low gross N_2O production. As a result, this implementation functioned primarily as mitigation for anthropogenic fertilizer emissions rather than as atmospheric N_2O removal. The efficacy is strongly dependent on the soil type, reflecting the role of soil structure and conditions on modulating the activity of these bacteria once they are present.[81] While inoculation of organic waste fertilizer is a promising distribution pathway for managed ecosystems, operation under oxic conditions and at atmospheric N_2O concentration remains the major constraint on achieving net N_2O uptake.

An emerging set of greenhouse gas flux data on tree surfaces has indicated a potentially significant N_2O sink through bark and leaves, including several observations of ecosystem-level net uptake.[84–87] High uptake rates from beech stems (0.254 µg N_2O m^{-2} h^{-1}) and shoots (4.54 µg N_2O m^{-2} h^{-1}) in a temperate upland forest in Germany result in a net ecosystem uptake rate of 1.99 kg N_2O ha^{-1} y^{-1} (0.54 tCO_2e ha^{-1} y^{-1}), including a small net emission from the soil.[86] A second study of beech trees identified a cryptogamic stem cover as a likely mechanism for stem uptake based on laboratory incubations of cryptogamic lichens and mosses.[85] Extremely high soil net uptake of 9.16 kg N_2O ha^{-1} y^{-1} (2.50 tCO_2e ha^{-1} y^{-1}) was also recorded at this site, which may be attributed to similar lichen and moss populations. While promising, these findings are highly unusual and should be independently confirmed. Yet, if the behavior of these cryptogam communities can be verified and understood, it may be possible to incubate and inoculate these species into other forest ecosystems, potentially via existing biosolids application programs or dedicated spore slurry spreading, although such techniques are not well developed.[88] While these results are promising indicators of a potential mechanism that could be leveraged, caution must be taken when extrapolating these results. Short-term measurements may not capture diurnal, seasonal, or weather-related variability and intra- and inter-tree spatial variability is not well sampled.

Modifying soil biogeochemistry: Broader changes to land use or management practices have been shown to increase N_2O consumption by altering soil biogeochemistry. Multi-year field data from enhanced rock weathering and biochar additions, for example, show marginal reductions in net N_2O emissions (~0.01 tCO_2e ha^{-1} y^{-1}) that are attributed to an observed pH shift from the ground rock.[66] Several studies of agroforestry, the addition of cultivated trees onto cropland, have observed reduced N_2O emissions compared to a monocrop control.[89–91] As expected, the unfertilized tree rows have lower net N_2O emission than the adjacent fertilized crop rows but their effect on N_2O fluxes extends into the adjacent crop area, both increasing gross uptake and reducing gross emission proportional to proximity.[91] The highest efficacy field showed a 55% net N_2O emissions reduction of 2.2 kg N_2O ha^{-1} y^{-1} (0.6 tCO_2e ha^{-1} y^{-1}) for a replacement of 20% of crop area with trees.[91] Within the affected crop area, total mineral N and water-filled pore space correlate with gross emission while no correlation was found between gross N_2O uptake and any measured soil variables, highlighting the difficulty of developing similar interventions to achieve net N_2O uptake.[91] The variability of N_2O emissions and consumption in natural environments can also be used to understand biogeochemical controls. A study of a tropical forest found a shift from net production to consumption for soil and stem N_2O fluxes along a

topographic gradient, corresponding to the changes in soil type, temperature, and water content.[87] Here, stem uptake up to 0.9 kg N_2O ha^{-1} y^{-1} (0.25 tCO_2e ha^{-1} y^{-1}) and soil uptake of 2.1 kg N_2O ha^{-1} y^{-1} (0.6 tCO_2e ha^{-1} y^{-1}) were observed.[87] While further studies may better determine the mechanism of variation here, it may be difficult or impossible to manage the relevant biogeochemical factors.

Interventions targeting N_2O fixation: While existing nitrogen fixation results in a small gross consumption of N_2O (e.g., ~0.03% in a study of artificial freshwater ponds[70]; additional information reported in Section S11.1), a much larger fraction of fixed nitrogen is ultimately released as N_2O (5.9% of global reactive nitrogen input to the land is released as N_2O),[92] resulting in increased gross production over the life cycle of the fixed nitrogen. Thus, an intervention here would require either a modification of the existing nitrogenase-based pathway to increase the preference for N_2O by several orders of magnitude, or the development of a novel fixation pathway that is highly selective for N_2O. Efforts to engineer nitrogenase have historically faced difficult constraints: nitrogenase is strongly inhibited by oxygen, energetically costly to operate, and governed by complex multi-subunit metalloclusters whose assembly is sensitive to intracellular redox state.[93,94] While, to my knowledge, no nitrogenase engineering efforts have focused on increasing N_2O affinity, the thermodynamic advantage of N_2O fixation indicates a theoretical path to increasing nitrogen fixation rates, which are typically energy-limited. Yet, if the increased preference for N_2O results in a decreased affinity for N_2, the total nitrogen fixation rate is likely to decrease due to the lower concentration, and thus availability, of N_2O. This risk is particularly salient for crops like soy, alfalfa, and groundnuts, whose high nitrogen fixation rate is both desirable and more likely to be constrained by mass transfer, not just energy availability.

Heterologous expression: Expressing N_2OR in crop leaves has been proposed as a scalable deployment strategy,[95,96] paralleling similar efforts to improve nitrogen fixation via nitrogenase expression[94,97] and CH_4 uptake via methane monooxygenase (MMO) expression.[98] N_2OR has been successfully expressed in tobacco leaves, although activity was only assessed for extracted proteins in an anoxic and highly reducing artificial atmosphere.[99] This activity is extrapolated to a seasonal uptake rate of 60,000 kg N_2O ha^{-1} by assuming the same enzymatic activity on leaves in air as in the assay,[95] a highly optimistic upper bound that does not account for the large differences between assay conditions and real agricultural environments. While the primary motivation for expression in crops is to leverage the leaf's high rate of atmospheric exchange, leaves are, because of this, highly oxygenated and not well suited to N_2OR activity, presenting a challenge for high real-world consumption even if N_2OR is functionally expressed and assembled.

There is increasing commercial interest in using microbe-based inoculants to increase yield, reduce reliance on synthetic fertilizers and pesticides, and improve crop tolerance to drought and environmental stressors, although many new-to-market products remain unproven.[100] Among existing practices, seed inoculation is the lowest-cost delivery method, requiring minimal modification to standard field operations and no additional dispersal costs. This technique is

widely used to improve N_2 fixation in soy at a total cost of \$17–24 ha^{-1}.[101] Liquid microbial inoculants are also becoming more common, delivered either as soil amendments or via foliar sprays, with retail costs of \$13–30 ha^{-1},[100,102,103] and all-in costs, including application labor and equipment, of \$30–52 ha^{-1}.[104,105] Liquid inoculants can sometimes be co-applied with fertilizers or pesticides, although in many cases a dedicated spray pass is required due to chemical compatibility and timing constraints. Lichen restoration has also been explored in other contexts, with small-scale academic trials reporting costs of \$450–4,170 ha^{-1} for manual collection and dispersal.[88] A single application has proven sufficient to re-establish mature lichen mats in previously disturbed but otherwise suitable ecosystems, indicating an annualized cost that may be comparable to microbial inoculation, e.g., \$67 ha^{-1} y^{-1} for a cost of \$450 ha^{-1} distributed over 10 years at an 8% discount rate.[106]

S11.1. Evidence for Direct Fixation of N_2O

Historical laboratory evidence demonstrates that nitrogen-fixing organisms can use N_2O when it is abundant, suggesting a potential additional path for uptake enhancement. A 1954 study using ^{15}N-labeled N_2O reported incorporation into soybean nodules and Azotobacter vinelandii after exposures to 0.1 atm N_2O in an otherwise N_2-free environment.[73] Additionally, N_2O has a thermodynamic advantage over N_2 as a substrate for NH_3 given its higher reactivity.[107] While these results demonstrate a plausible mechanism, they rely on N_2O concentrations many orders of magnitude above atmospheric levels and cannot, on their own, establish that direct N_2O fixation is climatologically or ecologically relevant at ambient conditions.

The primary ecological evidence for direct biological fixation of N_2O to NH_3 or NH_4^+ via the canonical nitrogen-fixation pathway consists of observations of N_2O undersaturation in shallow, oxygenated freshwater and marine systems, in which denitrification is inhibited by the oxygen-rich conditions.

Undersaturation, dissolved N_2O concentrations below those expected from Henry's law equilibrium with atmospheric concentrations, is indicative of a diffusional flux from the atmosphere into the aquatic environment and thus a net N_2O uptake within the aquatic environment. Saturation of 70–90% has been documented across a range of N-poor aquatic environments, with rare cases as low as 13–34%.[70] Because ammonium and nitrite availability increase gross N_2O production, thereby suppressing undersaturation signals, these observations are most evident in strongly N-limited systems where gross N_2O production is minimal, yet this mechanism may be active under a wider range of conditions.

Si et al. estimated an atmospheric N_2O influx of 0.21 kg N_2O ha^{-1} y^{-1} (0.06 tCO_2e ha^{-1} y^{-1}) based on observed undersaturation in experimental freshwater ponds.[70] However, these systems appear to have atypically high nitrogen fixation: the same study reported N_2 fixation rates near 400 kg N ha^{-1} y^{-1}, one to two orders of magnitude higher than typical aquatic rates (global median 8.7 kg N ha^{-1} y^{-1}).[108] The relative uptake of N_2O and N_2, 0.03% of N_2 fixation, is 800 times higher than the ratio of their atmospheric concentrations. A factor of ~40 enhancement

can be attributed to the greater solubility of N_2O than N_2 (24.2 versus 0.652 mM atm^{-1} in pure water).[21] The remaining factor of ~20 enhancement may support the conclusion of a lower activation energy for N_2O fixation based on the 18% lower ΔG for conversion to NH_3.[70]

However, a lab study attempting to directly observe assimilation of N_2O-N in soil samples exposed to N_2O in an N_2-free environment found no statistically significant assimilation despite significant N_2O consumption.[109] This suggests that N_2O fixation is not a universal microbial capability, despite the thermodynamic advantage, and may be restricted to particular nitrogenase variants or environmental conditions.

S12. Additional Analysis for Section 4.4

Using eddy covariance flux towers to monitor N_2O, CH_4, and CO_2 fluxes across a landscape is the gold standard for MRV for novel open-system approaches. The cost of a single tower, which can cover an area of 100 ha, is estimated at $160,000 (2022 Australian dollars).[110,111] In 2025 USD, this is $114,000 (Table S2). Assuming a ten-year project lifetime and a discount rate of 8%, this is \$170 ha^{-1} y^{-1}.

For an example of a hypothetical large-scale deployment of photocatalyst powder for atmospheric removal, the northern half of Australia is identified as having promising regional characteristics: an annualized irradiation above 6 kWh m^{-2} d^{-1} (compared to 3.5–5.5 kWh m^{-2} d^{-1} for the continental US) and a dry season which may extend the lifetime of catalyst powders from weeks to months.[112,113] Many other regions combine elements of infrequent precipitation, and high irradiance (e.g., equatorial, high altitude). Extrapolating from the field trial reported in Section 4.2 and annualized as in Section S6, four annual applications in such a setting, each with a catalyst persistence of 12 weeks, could achieve $C_{open} \approx \$11–45\ tCO_2e^{-1}$ ($R_{CO2e} \approx 1.8–7$ $tCO_2e\ ha^{-1}\ y^{-1}$ at a cost of \$80 ha^{-1} y^{-1}). Including MRV at the previously estimated rate of \$170 ha^{-1} y^{-1} yields $C_{open} \approx$ \$35–142 per tCO_2e. Here it is assumed that there are no other significant N_2O sources or sinks which would alter the ground-level atmospheric N_2O concentration. For areas with net N_2O production, such as agricultural fields, a higher instantaneous removal rate may be possible due to the elevated ground-level N_2O concentration, yet this elevation has high temporal variability, typically peaking following fertilization and precipitation events.

S13. Additional Analysis for Section 5

This section contains a brief narrative on how the review and analysis in Sections 3 and 4 are synthesized to determine the *feasibility level* and *key constraint*, produce a quantitative estimate of the *viability threshold*, and select a *key research need* for each approach.

The closed-system cost model defines a $C_{net} < \$200\ tCO_2e^{-1}$ Pareto frontier as a function of SEC and CAPEX. The knee of this frontier is approximately SEC = 0.2 Wh m^{-3} and CAPEX = $2

cfm^{-1} (Section 3.1 and S2), which is taken to be a representative set of assumptions for an optimal system design which balances trade-offs across these costs.

Table S11. Consolidated quantitative assessment of N_2O removal approaches[a]

**Mechanism**	**Approach**	**Performance: demonstrated or modeled**	**Required performance and gap**	**Estimated cost range**	**Cost uncertainties and omissions**	**Evidence basis for cost estimate**	**Key research need**	**Research priority**
Thermal and thermocatalysis	Thermal reactor	None at atmospheric concentration. Modeled from a commercial RTO at 750 °C with ε_{HX} = 97%: SEC = 7.64 Wh m^{-3} (Section 3.2).	SEC = 0.2 Wh m^{-3}, requiring ε_{HX} = 99.92%, a 38-fold reduction in heat loss from the state of the art.	Essentially climate-neutral (net removal ~8% of gross); SEC is within 10% of the ~8 Wh m^{-3} climate-neutral threshold.	Scaling of RTO capital cost to very high heat-recovery efficiency is unknown.	Mature commercial RTO cost and performance data.	Identify the cost-efficiency frontier of large high-efficiency heat exchangers.	Low.
	Thermocatalytic reactor	None. Modeled for a hypothetical best-available system at T_{rxn} = 200 °C (best reported catalytic T_{50}), ε_{HX} = 97%, CAPEX = $44 cfm^{-1}: SEC = 1.75 Wh m^{-3} (Section 3.2).	SEC = 0.2 Wh m^{-3}, a tenfold reduction, via T_{rxn} near 45 °C, ε_{HX} = 99.7%, or a combination.	Approximately $4,400 tCO_2e^{-1}; below $200 tCO_2e^{-1} only with waste heat, which constrains scale.	Catalyst lifetime and replacement cost; scaling of RTO capital cost to a low-temperature design.	Commercial RTO cost data with the best reported catalyst T_{50}; no relevant reaction kinetics.	High-throughput assay of thermal catalysts for reaction kinetics and H_2O inhibition at atmospheric concentration.	Medium.
Photolysis	Photolytic reactor	None. Modeled for KrBr* (207 nm) excimer lamps with AQY_{N2O} = 0.3% and η_{rad} = 5–18%: SEC of approximately 4 Wh m^{-3} (Section 3.3).	SEC = 0.2 Wh m^{-3}; the modeled ideal limit at 201 nm with η_{rad} = 100% is 0.5 Wh m^{-3}, still 2.5 times the target.	≈ $2,500 tCO_2e^{-1} at 18% radiant efficiency; ≈ $150 tCO_2e^{-1} at the idealized limit.	O_3 treatment capital and energy are absent from the model; lamp lifetime and replacement.	Simplified photolysis model developed here and unvalidated.	Lab-scale photolytic reactor with integrated in situ O_3 treatment.	Medium.
Gas-phase radical	Gas-phase radical reactor	None. Kinetic limit AQY_{N2O} < 1 × 10^{-8} under tropospheric conditions (Section 3.4).	$AQY_{N2O} \cdot \eta_{rad}$ > 0.63%; a six-order-of-magnitude gap.	No path to a climate-beneficial or economically plausible system.	Not applicable; no cost estimate is warranted.	Established gas-phase kinetics; no cost model attempted.	None identified.	None. No targeted research is recommended.
Photocatalysis	Shared foundation	No photocatalyst AQY_{N2O} has been measured at atmospheric concentration in any deployment format. Every cost estimate below scales inversely with one inferred value, so the four rows that follow are not independent estimates.				AQY_{N2O} = 0.02–0.04% inferred from the N_2O flux of a single field trial, with order-of-magnitude uncertainty from surface	High-throughput assay of photocatalysts for AQY_{N2O}, competing reactions, and humidity	High. Informs every photocatalytic approach.

						coverage, light, and N_2O flux (Section 3.5.2 and Section S6).	dependence at atmospheric concentration.	
	Photocatalytic reactor	None. Modeled SEC = 5–11 Wh m^{-3} and LED module CAPEX = \$30–60 cfm^{-1} using inferred AQY_{N2O} (Section 3.5).	$AQY_{N2O} \cdot \eta_{rad}$ > 0.63% for economic plausibility and >0.015% for climate viability, ~25–50 times the inferred performance.	Not climate-beneficial at the lower end of the modeled range, SEC = 5–11 Wh m^{-3}.	LED lifetime and replacement; reactor housing and air handling beyond the LED modules.	Component-level LED module cost only; no reactor has been built or costed.	None specific to this approach.	Low.
	Photocatalytic coatings (static surfaces)	No N_2O field or laboratory data for a photocatalyst in a coating format under ambient conditions (Section 4.2).	AQY_{N2O} ≈ 0.08% or more, twice the upper estimate, to reach \$200 tCO_2e^{-1} in the lower-cost case; the upper-cost case cannot reach \$200 tCO_2e^{-1} (Section S9.5).	\$380–1,400 tCO_2e^{-1} at C_A = \$8,400–30,700 ha^{-1} y^{-1} and AQY_{N2O} = 0.04%, for a coating whose area is entirely active catalyst.	Coating longevity and reapplication interval; labor dominates C_A and varies roughly fivefold across the surveyed markets.	Commercial roof coating and maintenance reports; R_{CO2e} estimated from the powder-dispersal AQY_{N2O} (Section S9.5).	Field trials with performance, longevity, and environmental impact monitoring across seasons and sites.	Medium.
	Photocatalytic powders (field-applied)	A single study reported a 0.1 μg m^{-2} s^{-1} change in N_2O flux against control fields, annualized to 0.5–2 tCO_2e ha^{-1} over a 90-day season from 12 applications totaling 3 kg ha^{-1} (Section 4.2 and Section S6).	Sustained activity and attribution to photocatalytic reduction; replication across seasons and sites with a positive environmental and toxicological assessment.	\$120–480 tCO_2e^{-1} at C_A = \$240 ha^{-1} y^{-1}; approximately \$11–45 tCO_2e^{-1} in high-irradiation settings, or \$35–142 tCO_2e^{-1} including MRV (Section S12).	Attribution of flux change to a photocatalytic or biological effect; CH_4 and CO_2 fluxes and crop yield were not reported; reapplication frequency and material cost.	A single short-duration study with a proprietary catalyst, combined with commercial agricultural spraying costs.	Field trials measuring N_2O, CH_4, and CO_2 flux, catalyst coverage, irradiance, nitrogen cycle, and crop yield to attribute the flux change.	High. The only approach with open-system field data, and its projected cost range spans the benchmark, but attribution of the reported removal remains to be established.
	Photocatalytic aerosols	None; no experimental data on aerosol performance or environmental impact (Section 4.2).	Catalytic activity sustained for days to weeks while aerosolized, with a small-scale outdoor test establishing performance and	\$350–1,060 tCO_2e^{-1} modeled at a dispersal cost of \$0.80 kg^{-1} and an eight-day tropospheric lifetime.	Monitoring of a dispersing plume, and environmental monitoring cost, are absent from the modeled dispersal cost; the ultimate	One modeling study; no experimental characterization of the aerosol format.	Paired chamber and small-scale outdoor testing with comprehensive measurement of gas-phase reactions.	Medium.

			environmental impact.		fate of the catalyst material.			
Biological reduction	Shared foundation	N_2O reduction requires anoxic conditions at the enzyme, yet both approaches must operate in bulk aerobic air. No system, engineered or natural, has shown sustained net N_2O reduction under those conditions.				Few observations, no long-duration measurements, and no direct cost estimate at scale for either approach.	Laboratory screening of candidate communities for sustained N_2O reduction under bulk aerobic conditions.	High. Informs both biological approaches.
	Bioreactor	14–18% removal at 0.43–0.56 ppm on swine-barn exhaust; scaled to 100% removal at 0.339 ppm, CAPEX = \$10.56 cfm^{-1} and SEC = 0.28 Wh m^{-3} (Section 3.6).	CAPEX < \$2 cfm^{-1}, requiring removal efficiency above 90% in a low-cost biofilter architecture, a 5.3-fold reduction in effective CAPEX.	Approximately \$800 tCO_2e^{-1}.	Media replacement, nutrient and water supply, and biological maintenance are absent from the scaled capital cost.	One pilot biofilter, scaled here to 100% removal; its bacterial community was not characterized.	Assay of natural and engineered communities for N_2O reduction kinetics and O_2 inhibition under controlled biofilter conditions.	High. Effective CAPEX is within a factor of about five of the target even from pilot-scale data.
	Ecosystem uptake enhancement	Short-duration net uptake of order 1 tCO_2e ha^{-1} y^{-1} in natural analogs; lichen communities 0.5–2.5 tCO_2e ha^{-1} y^{-1} (Section 4.3).	Season-average net uptake above 1 tCO_2e ha^{-1} y^{-1} across multiple sites and conditions. Reported uptake is instantaneous or short-duration.	\$25 tCO_2e^{-1} at C_A = \$50 ha^{-1} y^{-1} and 2 tCO_2e ha^{-1} y^{-1}, spanning ≈ \$7–340 tCO2e-1 across the cost and uptake ranges in Section 4.3.	MRV at \$170 ha^{-1} y^{-1} (Section S12); intervention persistence and reapplication frequency.	Removal rate from short-duration flux measurements in natural analogs; cost by analogy to commercial seed and liquid inoculant pricing.	Identify or engineer bacterial communities that sustain N_2O reduction under natural or managed field conditions.	Medium.

[a]Values are reproduced from the sections cited in each cell. Approaches are grouped by removal mechanism, with a *Shared foundation* row included for photocatalysis and biological reduction to highlight a common dependency. Performance figures are labeled as demonstrated or modeled; "modeled" indicates an output of the cost models rather than a measurement. Costs are based on the closed- and open-system cost models (Sections 3.1 and 4.1), not techno-economic assessments, and are stated only to the precision those models support. Research priority reflects the expected value of the decisive next experiment, combining how far it would resolve the key constraint with how tractable it is. For example, an approach at a low feasibility level can carry a high research priority when a single tractable experiment would resolve what limits it.

Thermal reactor: The feasibility level of 1 indicates that technical efficacy is proven (a significant body of work has shown thermal decomposition above 700 °C). Without heat recovery, the energy required to supply this heat would result in net positive emissions for any realistic energy emissions intensity: a non-catalytic process at 750 °C has SEC = 255 Wh m^{-3}. Even with state-of-the-art heat recovery efficiency of ε_{HX} = 97%, SEC = 7.6 Wh m^{-3}, essentially at the ~8 Wh m^{-3} climate-neutral threshold (Figure 3). The net removal is then only ~8% of the gross, and it becomes negative for electricity with life-cycle emissions above 22 g CO_2e kWh^{-1}, barely above the 20 g CO_2e kWh^{-1} assumed here for new solar photovoltaics. Because the cost model omits other life-cycle emissions, which would only narrow this margin, climate viability is not established under realistic operating conditions. To achieve SEC = 0.2 Wh m^{-3} would require heat recovery of 99.92%. While such an efficiency, which represents roughly a 38-fold reduction in unrecovered heat from the current state of the art, is theoretically possible, it would require major breakthroughs in heat exchanger design, and an R&D program is unlikely to achieve this goal. However, research to better identify the cost-efficiency frontier of large high-efficiency heat exchangers is highly relevant to thermocatalytic reactors and thermal destruction at higher concentrations, where the value of improving heat recovery is high, even at more modest performance levels.

Thermocatalytic reactor: Assuming a state-of-the-art reaction temperature of 200 °C and heat recovery efficiency of ε_{HX} = 97%, SEC = 1.75 Wh m^{-3}, below the climate-neutral threshold of ~8 Wh m^{-3} but above the feasibility target of 0.2 Wh m^{-3}, indicating climate viability. The similarity of a thermocatalytic system to existing industrial processes and the lack of evidence for any significant environmental side effects or undesirable reaction products are taken as sufficient evidence of *Environmental and social viability*, resulting in a feasibility level of 3. Here, the key constraint is again the heating energy requirement. The required tenfold reduction in SEC could be achieved, in theory, by either reducing the reaction temperature to ~45 °C, improving the heat recovery efficiency to 99.7%, or a combination thereof. Of the two, I select the catalyst performance as the *key research need* given the existing lack of experimental investigation at atmospheric concentration. Using recent advances in high-throughput assaying of thermal catalyst materials, a single research effort could quickly screen a wide range of known and novel N_2O thermal catalysts to develop improved models of reaction kinetics at atmospheric concentration, inhibition from H_2O, and, potentially, identify candidates with higher reactivity.

Photolytic reactor: Low-concentration N_2O photolysis by UV light is well characterized as the dominant natural sink in the stratosphere, a strong indicator for *technical efficacy* at tropospheric conditions. Using existing KrBr* (207 nm) and ArF* (193 nm) excimer lamps may result in an initial energy requirement of SEC ≈ 4 Wh m^{-3}, indicating *climate viability*. Yet, I consider this unproven because: a) The simplified photolysis model developed here is not sufficiently validated to give confidence in such a quantitative conclusion (e.g., a variation in SEC by a factor of two in either direction seems possible with the inclusion of additional factors like the full emission spectrum of the excimer lamp sources, losses from non-ideal internal reflectance, or absorption by additional trace gas species not included), and b) the co-production of O_3 would rapidly increase SEC as the O_3 concentration increases and no strategy for preventing this

build-up has been proposed. This O_3 production is also a significant concern for environmental and human impacts if it is released in the exhaust. While a range of O_3 destruction and treatment technologies exist for other applications, I consider the *environmental and social viability* unproven until an experimental demonstration of N_2O photolysis with integrated O_3 treatment due to the severity of this issue. I identify O_3 production as the *key constraint* as it is the most salient limiting factor for both *climate viability* and *environmental and social viability*. While the simplified photolysis model indicates an ideal performance limit of SEC = 0.5 Wh m^{-3}, greater than the 0.2 Wh m^{-3} target for *economic plausibility*, I do not take this as sufficient evidence for ruling out this approach. Rather, given other desirable process attributes, a more thorough second-pass analysis, including the development of a lab-scale experimental system and integration of O_3 treatment, is identified as the *key research need*. In particular, I hypothesize that a method for in situ O_3 treatment with extremely low energy use is required in order to mitigate the effect of O_3 on the AQY_{N2O} and not increase SEC. Even if the finding of economic nonviability is confirmed, this approach may be relevant for high-value niches, higher-concentration sources, or removal from low-O_2 air flows.

Gas-phase radical reactor: The analysis in Section 3.4 indicates that radical destruction via $O(^1D)$ shows *technical efficacy* at tropospheric conditions. Yet, the extremely low AQY_{N2O} of any radical reaction at these conditions ($<1 \times 10^{-8}$), taken as the *key constraint*, indicates that *climate viability* and *economic plausibility* are not possible. As such, there does not appear to be any path to *economic plausibility* for this approach and no targeted research is recommended.

Photocatalytic reactor: Photocatalytic removal at near-ambient concentration has been reported, indicating *technical efficacy*. The *key constraint* is the lighting energy requirement, which is primarily determined by the photocatalyst's AQY_{N2O} and the light source's radiant efficiency (η_{rad}). *Climate viability* requires $AQY_{N2O} \cdot \eta_{rad} > 0.015\%$ and *economic plausibility* requires $AQY_{N2O} \cdot \eta_{rad} > 0.63\%$ under the conservative component-level targets used as the *viability threshold*. The coupled lighting model crosses the \$200 tCO_2e^{-1} contour at a lower yield–efficiency product of approximately 0.45%. There is little experimental characterization of AQY_{N2O} across any experimental conditions and no direct reports at atmospheric conditions, although the analysis here indicates AQY_{N2O} = 0.02–0.04% for one outdoor study. Given this, *climate viability* is considered unproven. To address the lack of AQY_{N2O} characterization, the *key research need* is a broad assay of known and novel photocatalytic materials. This could be achieved by a single research effort using a high-throughput reactor system. Additional characterization of reaction kinetics and inhibition from H_2O could also inform reactor scaling. Observation of potential co-reactions is also needed to identify potential *environmental and social viability* concerns.

Bioreactor: Biological reaction of N_2O at near-ambient concentration has been demonstrated in a biofilter, indicating *technical efficacy* with CAPEX = \$10.56 cfm^{-1} and SEC = 0.28 Wh m^{-3} (both adjusted to account for the low reported removal efficiency). This performance further indicates *climate viability*. None of the N_2O bioreactor studies for low-concentration point-source

mitigation reveal any pollutant production or discernible adverse side effects, indicating *environmental and social viability*.

CAPEX and SEC are within factors of about five and 1.4, respectively, of their corresponding *economic plausibility* targets. Both are influenced by the removal efficiency: these adjusted numbers are the effective CAPEX and SEC for 100% removal efficiency based on the reported CAPEX = \$1.76 cfm^{-1} and SEC = 0.047 Wh m^{-3} at a 14–18.3% removal efficiency. Thus, the removal efficiency is taken as the *key constraint*. Further, the removal efficiency is related to a core mechanistic challenge, N_2O reduction under aerobic conditions. All else equal, an increase of removal efficiency to >90% for a low-cost biofilter architecture is needed to reduce the effective CAPEX to below the \$2 cfm^{-1} target for *economic plausibility*. The primary lever for increasing removal efficiency is the bacterial community in the bioreactor, which was not characterized for the referenced biofilter. Further, no applicable reference data exists across a range of N_2O-reducing bacteria under atmospheric conditions. Thus, the *key research need* is an assay of bacterial cultures from environments with known gross N_2O uptake to assess N_2O reduction kinetics, O_2 inhibition, and other metabolic performance metrics while under ambient conditions. Directed evolution or other forms of microbe engineering could also be used to improve performance.

Photocatalytic surfaces: Of the range of open-system photocatalytic formats, the term specifically refers to the application of photocatalysts to static or mobile surfaces. Powder dispersal has shown preliminary indications of *technical efficacy*; coatings have not yet been tested. Of the implementations explored, the release of a powder onto cropped fields via existing dispensing technology is the lowest-cost deployment method; a hypothetical large-scale deployment has a projected cost of C_{open} ≈ \$120–480 tCO_2e^{-1}, spanning the benchmark, based on the reported removal rate and dispersal costs from industrial agricultural spraying. I do not consider this first-order model as sufficient evidence to prove *economic plausibility*, and the reported field flux would indicate *climate viability* only once its statistical significance and its attribution to photocatalysis are established (barring unforeseen increases in CH_4 or CO_2 emissions). The photocatalytic powder's effect on crop yields and the ultimate fate in the environment, among other factors, were not reported, leaving *environmental and social viability* unproven. This approach is only supported by evidence from a single research effort of short duration using a proprietary catalyst. The specific performance of this proprietary catalyst does not appear to be a key enabling factor. Bueno-Alejo et al. report a reaction rate 51% greater than that of TiO_2 at 365 nm. Thus, to first order, a TiO_2 catalyst could generate similar performance with an increased areal mass loading of 51%. The material cost is ~1% of the projected removal cost, thus using a greater mass of TiO_2, or a smaller mass of a more expensive catalyst, does not appear to have a significant effect on the total cost. Thus, the *key constraint* is the lack of statistically robust, attributable performance data and of environmental impact data, and the *key research need* is additional field trials with more refined greenhouse gas flux measurement, improved quantification of AQY_{N2O}, N_2O removal rate, albedo changes, powder longevity, and other environmental impacts. A positive environmental impact assessment, no toxicological concerns with the chosen catalyst material, and the ability to

replicate the reported performance across multiple growing seasons and field sites would constitute the *viability threshold*.

Photocatalytic aerosols: This term specifically refers to the release of photocatalytic particles into the troposphere (or at high elevation) with intent for activity to occur while the particle is aerosolized, rather than once settled. The hypothetical use of the same catalyst materials used for photocatalytic surfaces is sufficient to indicate *technical efficacy* and *climate viability*. Yet, *economic plausibility* is dependent on the catalyst's activity being maintained for days to weeks while aerosolized and settling through the troposphere. While surface-based photocatalysts have shown continuous activity over these timescales, experimental characterization of aerosol performance (e.g., in enclosed environmental chambers) will be needed to increase confidence in the performance of this format and identify any phenomena unique to this format. The primary distinction between photocatalysts applied to surfaces versus dispersed in the air is the degree of control over the location of activity: it is more difficult to control the dispersion of aerosol particles and may be impossible to ensure they remain confined to an experimental site. While the potential adverse environmental and health effects are broadly similar for both, the reduced ability to contain these effects spatially, and the increased challenge of monitoring such impacts, increase the degree of certainty required for *environmental and social viability*. Thus, the *key constraint*, *viability threshold*, and *key research need* have an increased focus on reducing the uncertainty of environmental impact and developing monitoring/measurement capabilities.

Ecosystem uptake enhancement: The existence of natural ecosystems with net N_2O uptake, and laboratory measurement of N_2O consumption by microbial communities, indicate *technical efficacy*. Evidence of net greenhouse gas removal from these systems, and the anticipated low emissions associated with dispersal, indicate that *climate viability* is likely. Yet, the highest potential intervention, inoculation of N_2O-reducing bacteria onto managed land, is unlikely to achieve net N_2O removal due to O_2 inhibition of the primary N_2O reduction pathway, here taken as the *key constraint*. While such an intervention may still yield a net climate benefit by reducing N_2O emissions on land with high preexisting emissions, within the context of atmospheric removal I consider *climate viability* to be unproven until net N_2O uptake can be demonstrated. Based on natural analogs, N_2O fluxes are highly variable across time and space, with short-duration flux measurements of order 1 $tCO_2e\ ha^{-1}\ y^{-1}$. This rate is likely sufficient for *economic plausibility* if it can be maintained over time given the cost of comparable agricultural inoculants. Thus, the *viability threshold* is an average net uptake rate of >1 $tCO_2e\ ha^{-1}\ y^{-1}$ maintained over an entire growing season and demonstrated in multiple sites and conditions. Before conducting field trials, the *key research need* is to identify existing candidate bacteria or communities, or engineer new ones, which have increased O_2 tolerance or other protective mechanisms to maintain N_2O reduction under aerobic conditions.

References for Supporting Information

(1) IPCC, 2021: Annex III: Tables of Historical and Projected Well-Mixed Greenhouse Gas Mixing Ratios and Effective Radiative Forcing of All Climate Forcers. In *Climate Change 2021: The Physical Science Basis. Contribution of Working Group I to the Sixth Assessment Report of the Intergovernmental Panel on Climate Change*; Dentener, F. J., Hall, B., Smith, C., Eds.; Masson-Delmotte, V., Zhai, P., Pirani, A., Connors, S. L., Pean, C., Berger, S., Caud, N., Chen, Y., Goldfarb, L., Gomis, M. I., Huang, M., Leitzell, K., Lonnoy, E., Matthews, J. B. R., Maycock, T. K., Waterfield, T., Yelekci, O., Yu, R., Zhou, B., Series Eds.; Cambridge University Press: Cambridge, United Kingdom, 2021; pp 2139–2152. https://doi.org/10.1017/9781009157896.017

(2) Haynes, W. M. *CRC Handbook of Chemistry and Physics*; CRC Press, 2016.

(3) Smith, C.; Nicholls, Z. R. J.; Armour, K.; Collins, W.; Forster, P.; Meinshausen, M.; Palmer, M. D.; Watanabe, M. 2021: The Earth’s Energy Budget, Climate Feedbacks, and Climate Sensitivity Supplementary Material. In *Climate Change 2021: The Physical Science Basis. Contribution of Working Group I to the Sixth Assessment Report of the Intergovernmental Panel on Climate Change*; Masson-Delmotte, V., Zhai, P., Pirani, A., Connors, S. L., Pean, C., Berger, S., Caud, N., Chen, Y., Goldfarb, L., Gomis, M. I., Huang, M., Leitzell, K., Lonnoy, E., Matthews, J. B. R., Maycock, T. K., Waterfield, T., Yelekci, O., Yu, R., Zhou, B., Eds.; Cambridge University Press, 2023. https://doi.org/10.1017/9781009157896

(4) National Oceanic and Atmospheric Administration. *The Atmosphere*. https://www.noaa.gov/jetstream/atmosphere (accessed 2026-01-14)

(5) Inman, M. Carbon Is Forever. *Nat. Clim. Change* **2008**, *1*, 156–158. https://doi.org/10.1038/climate.2008.122

(6) Lan, X.; Thoning, K. W.; Dlugokencky, E. J. Trends in Globally Averaged CH4, N2O, and SF6 Determined from NOAA Global Monitoring Laboratory Measurements. Version 2025-12, 2025. https://doi.org/10.15138/P8XG-AA10

(7) Myhre, G.; Shindell, D.; Breon, F.-M.; Collins, W.; Fuglestvedt, J.; Huang, J.; Koch, D.; Lamarque, J.-F.; Lee, D.; Mendoza, B.; Nakajima, T.; Robock, A.; Stephens, G.; Takemura, T.; Zhang, H. 2013: Anthropogenic and Natural Radiative Forcing Supplementary Material. In *Climate Change 2013: The Physical Science Basis. Contribution of Working Group I to the Fifth Assessment Report of the Intergovernmental Panel on Climate Change*; Stocker, T. F., Qin, D., Plattner, G.-K., Tignor, M., Allen, S. K., Boschung, J., Nauels, A., Xia, Y., Bex, V., Midgley, P. M., Eds.; Cambridge University Press, 2013.

(8) U.S. Department of the Treasury, Bureau of the Fiscal Service. *Currency Exchange Rates Converter Tool*. https://fiscaldata.treasury.gov/currency-exchange-rates-converter/ (accessed 2026-01-16)

(9) Federal Reserve Bank of Minneapolis. *Inflation Calculator*. Federal Reserve Bank of Minneapolis. https://www.minneapolisfed.org/about-us/monetary-policy/inflation-calculator (accessed 2026-07-06)

(10) Smith, B.; Sekar, A.; Mirletz, H.; Heath, G.; Margolis, R. *An Updated Life Cycle Assessment of Utility-Scale Solar Photovoltaic Systems Installed in the United States*; NREL/TP-7A40-87372; National Renewable Energy Laboratory, 2024; p NREL/TP--7A40-87372, 2331420, MainId:88147. https://doi.org/10.2172/2331420

(11) U.S. EIA. *Average Price of Electricity to Ultimate Customers by End-Use Sector, by State, 2022 and 2023*. https://www.eia.gov/electricity/annual/html/epa_02_10.html (accessed 2025-09-13)

(12) Vatankhah Ghadim, H.; Haas, J.; Breyer, C.; Gils, H. C.; Read, E. G.; Xiao, M.; Peer, R. Are We Too Pessimistic? Cost Projections for Solar Photovoltaics, Wind Power, and Batteries Are over-Estimating Actual Costs Globally. *Appl. Energy* **2025**, *390*, 125856. https://doi.org/10.1016/j.apenergy.2025.125856

(13) McQueen, N.; Desmond, M. J.; Socolow, R. H.; Psarras, P.; Wilcox, J. Natural Gas vs. Electricity for Solvent-Based Direct Air Capture. *Front. Clim.* **2021**, *2*. https://doi.org/10.3389/fclim.2020.618644

(14) Richard Mattus. *Best Practice Guidance on Ventilation Air Methane (VAM) Mitigation*; UNECE, 2025; p 56. https://unece.org/sites/default/files/2025-02/BPG%20VAM%20Mitigation%20Final%2012022025%20v2.pdf

(15) Yan, L.; Ren, T.; Wang, X.; Gao, Q.; Ji, D.; Suo, J. Excellent Catalytic Performance of ZnxCo1−xCo2O4 Spinel Catalysts for the Decomposition of Nitrous Oxide. *Catal. Commun.* **2003**, *4* (10), 505–509. https://doi.org/10.1016/S1566-7367(03)00131-6

(16) Tolcin, A. C. *Mineral Commodity Summaries 2026 - Zinc*; United States Geological Survey, 2026.

(17) Ewing, S. M. *Mineral Commodity Summaries 2026 - Cobalt*; United States Geological Survey, 2026.

(18) Lienhard, J. H. I.; Lienhard, J. H. V. *A Heat Transfer Textbook*, 5th Edition.; Dover, 2020.

(19) Forman, C.; Muritala, I. K.; Pardemann, R.; Meyer, B. Estimating the Global Waste Heat Potential. *Renew. Sustain. Energy Rev.* **2016**, *57*, 1568–1579. https://doi.org/10.1016/j.rser.2015.12.192

(20) Papapetrou, M.; Kosmadakis, G.; Cipollina, A.; La Commare, U.; Micale, G. Industrial Waste Heat: Estimation of the Technically Available Resource in the EU per Industrial Sector, Temperature Level and Country. *Appl. Therm. Eng.* **2018**, *138*, 207–216. https://doi.org/10.1016/j.applthermaleng.2018.04.043

(21) Burkholder, J. B.; Sander, S. P.; Abbatt, J.; Barker, J. R.; Cappa, C.; Crounse, J. D.; Dibble, T. S.; Huie, R. E.; Kolb, C. E.; Kurylo, M. J.; Orkin, V. L.; Percival, C. J.; Wilmouth, D. M.; Wine, P. H. *Chemical Kinetics and Photochemical Data for Use in Atmospheric Studies, Evaluation No. 19*; JPL Publication 19-5; Jet Propulsion Laboratory: Pasadena, 2019.

(22) Herman, J. R.; Mentall, J. E. O2 Absorption Cross Sections (187–225 Nm) from Stratospheric Solar Flux Measurements. *J. Geophys. Res. Oceans* **1982**, *87* (C11), 8967–8975. https://doi.org/10.1029/JC087iC11p08967

(23) Ranjan, S.; Schwieterman, E. W.; Harman, C.; Fateev, A.; Sousa-Silva, C.; Seager, S.; Hu, R. Photochemistry of Anoxic Abiotic Habitable Planet Atmospheres: Impact of New H2O Cross Sections. *Astrophys. J.* **2020**, *896* (2), 148. https://doi.org/10.3847/1538-4357/ab9363

(24) Shemansky, D. E. CO2 Extinction Coefficient 1700–3000 Å. *J. Chem. Phys.* **1972**, *56* (4), 1582–1587. https://doi.org/10.1063/1.1677408

(25) Gkouvelis, L.; Akın, C.; Heng, K. The Quantum Yield of O(^{1} S) in CO_2 Photolysis Retrieved from the Martian Atmosphere: The Quantum Yield of O(^{1} S) in CO_2 Photolysis. *Astron. Astrophys.* **2024**, *690*, A319. https://doi.org/10.1051/0004-6361/202450588

(26) Rauch, K. D.; MacIsaac, S. A.; Reid, B.; Mullin, T. J.; Atkinson, A. J.; Pimentel, A. L.; Stoddart, A. K.; Linden, K. G.; Gagnon, G. A. A Critical Review of Ultra-Violet Light Emitting Diodes as a One Water Disinfection Technology. *Water Res. X* **2024**, *25*, 100271. https://doi.org/10.1016/j.wroa.2024.100271

(27) Bergman, R. S. Germicidal UV Sources and Systems. *Photochem. Photobiol.* **2021**, *97* (3), 466–470. https://doi.org/10.1111/php.13387

(28) Zhang, H. J.; Han, Q. Y.; Zhang, S. D. 254 Nm Radiant Efficiency of High Output Low Pressure Mercury Discharge Lamps with Neon-Argon Buffer Gas. *Appl. Mech. Mater.* **2013**, *325–326*, 409–412. https://doi.org/10.4028/www.scientific.net/AMM.325-326.409

(29) An, R.; Han, Q.; Zhang, S. 185 Nm Radiation Measurement of High Output Low Pressure Mercury Lamps. In *Proceedings of the 2015 International Conference on Mechatronics, Electronic, Industrial and Control Engineering*; Atlantis Press, 2015; pp 719–722. https://doi.org/10.2991/meic-15.2015.164

(30) Kumagai, H.; Obara, M. New High-efficiency Quasi-continuous Operation of an ArF(B→X) Excimer Lamp Excited by Microwave Discharge. *Appl. Phys. Lett.* **1989**, *55* (15), 1583–1584. https://doi.org/10.1063/1.102247

(31) Obenschain, S. P.; Schmitt, A. J.; Bates, J. W.; Wolford, M. F.; Myers, M. C.; McGeoch, M. W.; Karasik, M.; Weaver, J. L. Direct Drive with the Argon Fluoride Laser as a Path to High Fusion Gain with Sub-Megajoule Laser Energy. *Philos. Trans. R. Soc. Math. Phys. Eng. Sci.* **2020**, *378* (2184), 20200031. https://doi.org/10.1098/rsta.2020.0031

(32) Sosnin, E. A.; Oppenländer, T.; Tarasenko, V. F. Applications of Capacitive and Barrier Discharge Excilamps in Photoscience. *J. Photochem. Photobiol. C Photochem. Rev.* **2006**, *7* (4), 145–163. https://doi.org/10.1016/j.jphotochemrev.2006.12.002

(33) Hashimoto, K.; Irie, H.; Fujishima, A. TiO2 Photocatalysis: A Historical Overview and Future Prospects. *Jpn. J. Appl. Phys.* **2005**, *44* (12R), 8269. https://doi.org/10.1143/JJAP.44.8269

(34) Sun, H.; Bai, Y.; Cheng, Y.; Jin, W.; Xu, N. Preparation and Characterization of Visible-Light-Driven Carbon−Sulfur-Codoped TiO2 Photocatalysts. *Ind. Eng. Chem. Res.* **2006**, *45* (14), 4971–4976. https://doi.org/10.1021/ie060350f

(35) Tokode, O.; Prabhu, R.; Lawton, L. A.; Robertson, P. K. J. UV LED Sources for Heterogeneous Photocatalysis. In *Environmental Photochemistry Part III*; Bahnemann, D. W., Robertson, P. K. J., Eds.; Springer: Berlin, Heidelberg, 2015; pp 159–179. https://doi.org/10.1007/698_2014_306

(36) Alalm, M. G.; Djellabi, R.; Meroni, D.; Pirola, C.; Bianchi, C. L.; Boffito, D. C. Toward Scaling-Up Photocatalytic Process for Multiphase Environmental Applications. *Catalysts* **2021**, *11* (5), 562. https://doi.org/10.3390/catal11050562

(37) Bellobono, I. R.; Rossi, M.; Testino, A.; Morazzoni, F.; Bianchi, R.; De Martini, G.; Tozzi, P. M.; Stanescu, R.; Costache, C.; Bobirica, L.; Bonardi, M. L.; Groppi, F. Influence of Irradiance, Flow Rate, Reactor Geometry, and Photopromoter Concentration in Mineralization Kinetics of Methane in Air and in Aqueous Solutions by Photocatalytic Membranes Immobilizing Titanium Dioxide. *Int. J. Photoenergy* **2008**, *2008* (1), 283741. https://doi.org/10.1155/2008/283741

(38) Bloh, J. Z. A Holistic Approach to Model the Kinetics of Photocatalytic Reactions. *Front. Chem.* **2019**, *7*. https://doi.org/10.3389/fchem.2019.00128

(39) Bueno-Alejo, C. J.; Khambhati, Y. K.; Papadopoulos, A.; Reli, M.; Ricka, R. Using Photocatalysis for Sustainable Agriculture: R-Leaf's Potential in Large-Scale N2O Mitigation. *J. Hazard. Mater. Adv.* **2025**, *18*, 100703. https://doi.org/10.1016/j.hazadv.2025.100703

(40) Kessler, M. I.; Randall, R.; Wan, G.; Xu, K.; Zhang, Y.; Dionne, J. A.; Jackson, R. B.; Majumdar, A. A Humidity-Tolerant Photocatalyst for Methane Removal. *Environ. Sci. Technol.* **2026**, *60* (7), 5442–5452. https://doi.org/10.1021/acs.est.5c16764

(41) Randall, R.; Jackson, R. B.; Majumdar, A. Cost Modeling of Photocatalytic Decomposition of Atmospheric Methane and Nitrous Oxide. *Environ. Res. Lett.* **2024**, *19* (6), 064015. https://doi.org/10.1088/1748-9326/ad4376

(42) Mino, L.; Cazzaniga, M.; Moriggi, F.; Ceotto, M. Elucidating NOx Surface Chemistry at the Anatase (101) Surface in TiO2 Nanoparticles. *J. Phys. Chem. C* **2022**, *127* (1), 437–449. https://doi.org/10.1021/acs.jpcc.2c07489

(43) Wanbayor, R.; Ruangpornvisuti, V. Adsorption of CO, H2, N2O, NH3 and CH4 on the Anatase TiO2 (001) and (101) Surfaces and Their Competitive Adsorption. *Mater. Chem. Phys.* **2010**, *124* (1), 720–725. https://doi.org/10.1016/j.matchemphys.2010.07.043

(44) Wang, L.; Song, W.; Deng, J.; Zheng, H.; Liu, J.; Zhao, Z.; Gao, M.; Wei, Y. Facet-Dependent Photocatalytic Decomposition of N2O on the Anatase TiO2: A DFT Study. *Nanoscale* **2018**, *10* (13), 6024–6038. https://doi.org/10.1039/c7nr09274a

(45) Devahasdin, S.; Fan, C.; Li, K.; Chen, D. H. TiO2 Photocatalytic Oxidation of Nitric Oxide: Transient Behavior and Reaction Kinetics. *J. Photochem. Photobiol. Chem.* **2003**, *156* (1), 161–170. https://doi.org/10.1016/S1010-6030(03)00005-4

(46) Henderson, M. A.; Epling, W. S.; Peden, C. H. F.; Perkins, C. L. Insights into Photoexcited Electron Scavenging Processes on TiO2 Obtained from Studies of the Reaction of O2 with OH Groups Adsorbed at Electronic Defects on TiO2(110). *J. Phys. Chem. B* **2003**, *107* (2), 534–545. https://doi.org/10.1021/jp0262113

(47) Obalová, L.; Reli, M.; Lang, J.; Matějka, V.; Kukutschová, J.; Lacný, Z.; Kočí, K. Photocatalytic Decomposition of Nitrous Oxide Using TiO2 and Ag-TiO2 Nanocomposite Thin Films. *Catal. Today* **2013**, *209*, 170–175. https://doi.org/10.1016/j.cattod.2012.11.012

(48) Obalová, L.; Šihor, M.; Praus, P.; Reli, M.; Kočí, K. Photocatalytic and Photochemical Decomposition of N2O on ZnS-MMT Catalyst. *Catal. Today* **2014**, *230*, 61–66. https://doi.org/10.1016/j.cattod.2013.09.047

(49) Zhao, S.; Choi, W. C-F Bond Integration in Polymeric Carbon Nitride Enables Oxygen-Tolerant Photocatalytic Reduction of NO to N2. *Nat. Commun.* **2025**, *16* (1), 11024. https://doi.org/10.1038/s41467-025-66008-z

(50) Bueno-Alejo, C. J.; Khambhati, Y. K.; Papadopoulos, A. Photocatalytic Removal of N2O in Cropped Fields Using R-Leaf. *Appl. Catal. O Open* **2025**, *201*, 207032. https://doi.org/10.1016/j.apcato.2025.207032

(51) Hood, M. C.; Shah, S. B.; Kolar, P.; Stikeleather, L. F. Design and Operation of a Biofilter for Treatment of Swine House Pit Ventilation Exhaust, ASABE Paper No. 1111023. In *ASABE*; American Society of Agricultural and Biological Engineers, 2011. https://doi.org/10.13031/2013.37394

(52) Wang, Z.; Vishwanathan, N.; Kowaliczko, S.; Ishii, S. Clarifying Microbial Nitrous Oxide Reduction under Aerobic Conditions: Tolerant, Intolerant, and Sensitive. *Microbiol. Spectr.* **2023**, *11* (2), e04709-22. https://doi.org/10.1128/spectrum.04709-22

(53) Han, H.; Kim, D. D.; Song, M. J.; Yun, T.; Yoon, H.; Lee, H. W.; Kim, Y. M.; Laureni, M.; Yoon, S. Biotrickling Filtration for the Reduction of N2O Emitted during Wastewater Treatment: Results from a Long-Term In Situ Pilot-Scale Testing. *Environ. Sci. Technol.* **2023**, *57* (9), 3883–3892. https://doi.org/10.1021/acs.est.2c08818

(54) Pritchard, D. T.; Currie, J. A. Diffusion of Coefficients of Carbon Dioxide, Nitrous Oxide, Ethylene and Ethane in Air and Their Measurement. *J. Soil Sci.* **1982**, *33* (2), 175–184. https://doi.org/10.1111/j.1365-2389.1982.tb01757.x

(55) Clear, R. D.; Gartland, L.; Winkelmann, F. C. An Empirical Correlation for the Outside Convective Air-Film Coefficient for Horizontal Roofs. *Energy Build.* **2003**, *35* (8), 797–811. https://doi.org/10.1016/S0378-7788(02)00240-2

(56) World Bank Group. *Global Wind Atlas*. https://globalwindatlas.info (accessed 2025-10-04)

(57) ASTM International. *Standard Tables for Reference Solar Spectral Irradiances: Direct Normal and Hemispherical on 37° Tilted Surface*. https://store.astm.org/g0173-03r20.html (accessed 2025-10-08)

(58) Billy J Roberts. *Global Horizontal Solar Irradiance*; NREL, 2018. https://www.nrel.gov/docs/libraries/gis/high-res-images/nsrdb-v3-ghi-2018-01.jpg? (accessed 2025-10-06)

(59) de Richter, R.; Ming, T.; Davies, P.; Liu, W.; Caillol, S. Removal of Non-CO2 Greenhouse Gases by Large-Scale Atmospheric Solar Photocatalysis. *Prog. Energy Combust. Sci.* **2017**, *60*, 68–96. https://doi.org/10.1016/j.pecs.2017.01.001

(60) Sproul, J.; Wan, M. P.; Mandel, B. H.; Rosenfeld, A. H. Economic Comparison of White, Green, and Black Flat Roofs in the United States. *Energy Build.* **2014**, *71*, 20–27. https://doi.org/10.1016/j.enbuild.2013.11.058

(61) Municipal Administration and Urban Development Department. *Telangana Cool Roof Policy 2023–2028*; Municipal Administration and Urban Development Department, 2023.

(62) Hubau, W.; Lewis, S. L.; Phillips, O. L.; Affum-Baffoe, K.; Beeckman, H.; Cuní-Sanchez, A.; Daniels, A. K.; Ewango, C. E. N.; Fauset, S.; Mukinzi, J. M.; Sheil, D.; Sonké, B.; Sullivan, M. J. P.; Sunderland, T. C. H.; Taedoumg, H.; Thomas, S. C.; White, L. J. T.; Abernethy, K. A.; Adu-Bredu, S.; Amani, C. A.; Baker, T. R.; Banin, L. F.; Baya, F.; Begne, S. K.; Bennett, A. C.; Benedet, F.; Bitariho, R.; Bocko, Y. E.; Boeckx, P.; Boundja, P.; Brienen, R. J. W.; Brncic, T.; Chezeaux, E.; Chuyong, G. B.; Clark, C. J.; Collins, M.; Comiskey, J. A.; Coomes, D. A.; Dargie, G. C.; de Haulleville, T.; Kamdem, M. N. D.; Doucet, J.-L.; Esquivel-Muelbert, A.; Feldpausch, T. R.; Fofanah, A.; Foli, E. G.; Gilpin, M.; Gloor, E.; Gonmadje, C.; Gourlet-Fleury, S.; Hall, J. S.; Hamilton, A. C.; Harris, D. J.; Hart, T. B.; Hockemba, M. B. N.; Hladik, A.; Ifo, S. A.; Jeffery, K. J.; Jucker, T.; Yakusu, E. K.; Kearsley, E.; Kenfack, D.; Koch, A.; Leal, M. E.; Levesley, A.; Lindsell, J. A.; Lisingo, J.; Lopez-Gonzalez, G.; Lovett, J. C.; Makana, J.-R.; Malhi, Y.; Marshall, A. R.; Martin, J.; Martin, E. H.; Mbayu, F. M.; Medjibe, V. P.; Mihindou, V.; Mitchard, E. T. A.; Moore, S.; Munishi, P. K. T.; Bengone, N. N.; Ojo, L.; Ondo, F. E.; Peh, K. S.-H.; Pickavance, G. C.; Poulsen, A. D.; Poulsen, J. R.; Qie, L.; Reitsma, J.; Rovero, F.; Swaine, M. D.; Talbot, J.; Taplin, J.; Taylor, D. M.; Thomas, D. W.; Toirambe, B.; Mukendi, J. T.; Tuagben, D.; Umunay, P. M.; van der Heijden, G. M. F.; Verbeeck, H.; Vleminckx, J.; Willcock, S.; Wöll, H.; Woods, J. T.; Zemagho, L. Asynchronous Carbon Sink Saturation in African and Amazonian Tropical Forests. *Nature* **2020**, *579* (7797), 80–87. https://doi.org/10.1038/s41586-020-2035-0

(63) Bernal, B.; Murray, L. T.; Pearson, T. R. H. Global Carbon Dioxide Removal Rates from Forest Landscape Restoration Activities. *Carbon Balance Manag.* **2018**, *13* (1), 22. https://doi.org/10.1186/s13021-018-0110-8

(64) Power, I. M.; Hatten, V. N. J.; Guo, M.; Schaffer, Z. R.; Rausis, K.; Klyn-Hesselink, H. Are Enhanced Rock Weathering Rates Overestimated? A Few Geochemical and Mineralogical Pitfalls. *Front. Clim.* **2025**, *6*. https://doi.org/10.3389/fclim.2024.1510747

(65) Zhao, X.; Mignone, B. K.; Wise, M. A.; McJeon, H. C. Trade-Offs in Land-Based Carbon Removal Measures under 1.5 °C and 2 °C Futures. *Nat. Commun.* **2024**, *15* (1), 2297. https://doi.org/10.1038/s41467-024-46575-3

(66) Anthony, T. L.; Jones, A. R.; Silver, W. L. Supplementing Enhanced Weathering With Organic Amendments Accelerates the Net Climate Benefit of Soil Amendments in Rangeland Soils. *AGU Adv.* **2025**, *6* (2), e2024AV001480. https://doi.org/10.1029/2024AV001480

(67) Zomer, R. J.; Bossio, D. A.; Sommer, R.; Verchot, L. V. Global Sequestration Potential of Increased Organic Carbon in Cropland Soils. *Sci. Rep.* **2017**, *7* (1), 15554. https://doi.org/10.1038/s41598-017-15794-8

(68) Stein, L. Y.; Klotz, M. G. The Nitrogen Cycle. *Curr. Biol.* **2016**, *26* (3), R94–R98. https://doi.org/10.1016/j.cub.2015.12.021

(69) Bakken, L. R.; Bergaust, L.; Liu, B.; Frostegård, Å. Regulation of Denitrification at the Cellular Level: A Clue to the Understanding of N2O Emissions from Soils. *Philos. Trans. R. Soc. B Biol. Sci.* **2012**, *367* (1593), 1226–1234. https://doi.org/10.1098/rstb.2011.0321

(70) Si, Y.; Zhu, Y.; Sanders, I.; Kinkel, D. B.; Purdy, K. J.; Trimmer, M. Direct Biological Fixation Provides a Freshwater Sink for N2O. *Nat. Commun.* **2023**, *14* (1), 6775. https://doi.org/10.1038/s41467-023-42481-2

(71) Thomson, A. J.; Giannopoulos, G.; Pretty, J.; Baggs, E. M.; Richardson, D. J. Biological Sources and Sinks of Nitrous Oxide and Strategies to Mitigate Emissions. *Philos. Trans. R. Soc. B Biol. Sci.* **2012**, *367* (1593), 1157–1168. https://doi.org/10.1098/rstb.2011.0415

(72) Zhang, L.; Wüst, A.; Prasser, B.; Müller, C.; Einsle, O. Functional Assembly of Nitrous Oxide Reductase Provides Insights into Copper Site Maturation. *Proc. Natl. Acad. Sci.* **2019**, *116* (26), 12822–12827. https://doi.org/10.1073/pnas.1903819116

(73) Mozen, M. M.; Burris, R. H. The Incorporation of 15N-Labelled Nitrous Oxide by Nitrogen Fixing Agents. *Biochim. Biophys. Acta* **1954**, *14*, 577–578. https://doi.org/10.1016/0006-3002(54)90243-7

(74) Yang, W. H.; Silver, W. L. Net Soil–Atmosphere Fluxes Mask Patterns in Gross Production and Consumption of Nitrous Oxide and Methane in a Managed Ecosystem. *Biogeosciences* **2016**, *13* (5), 1705–1715. https://doi.org/10.5194/bg-13-1705-2016

(75) Xu, R.; Tian, H.; Pan, S.; Prior, S. A.; Feng, Y.; Dangal, S. R. S. Global N2O Emissions From Cropland Driven by Nitrogen Addition and Environmental Factors: Comparison and Uncertainty Analysis. *Glob. Biogeochem. Cycles* **2020**, *34* (12), e2020GB006698. https://doi.org/10.1029/2020GB006698

(76) Syakila, A.; Kroeze, C.; Slomp, C. P. Neglecting Sinks for N_2O at the Earth's Surface: Does It Matter? *J. Integr. Environ. Sci.* **2010**, *7* (sup1), 79–87. https://doi.org/10.1080/1943815X.2010.497492

(77) Schlesinger, W. H. An Estimate of the Global Sink for Nitrous Oxide in Soils. *Glob. Change Biol.* **2013**, *19* (10), 2929–2931. https://doi.org/10.1111/gcb.12239

(78) Wen, Y.; Chen, Z.; Dannenmann, M.; Carminati, A.; Willibald, G.; Kiese, R.; Wolf, B.; Veldkamp, E.; Butterbach-Bahl, K.; Corre, M. D. Disentangling Gross N2O Production and Consumption in Soil. *Sci. Rep.* **2016**, *6* (1), 36517. https://doi.org/10.1038/srep36517

(79) Glass, J.; Orphan, V. J. Trace Metal Requirements for Microbial Enzymes Involved in the Production and Consumption of Methane and Nitrous Oxide. *Front. Microbiol.* **2012**, *3*. https://doi.org/10.3389/fmicb.2012.00061

(80) Giannopoulos, G.; Hartop, K. R.; Brown, B. L.; Song, B.; Elsgaard, L.; Franklin, R. B. Trace Metal Availability Affects Greenhouse Gas Emissions and Microbial Functional Group Abundance in Freshwater Wetland Sediments. *Front. Microbiol.* **2020**, *11*. https://doi.org/10.3389/fmicb.2020.560861

(81) Domeignoz-Horta, L. A.; Putz, M.; Spor, A.; Bru, D.; Breuil, M. C.; Hallin, S.; Philippot, L. Non-Denitrifying Nitrous Oxide-Reducing Bacteria - An Effective N2O Sink in Soil. *Soil Biol. Biochem.* **2016**, *103*, 376–379. https://doi.org/10.1016/j.soilbio.2016.09.010

(82) Jonassen, K. R.; Hagen, L. H.; Vick, S. H. W.; Arntzen, M. Ø.; Eijsink, V. G. H.; Frostegård, Å.; Lycus, P.; Molstad, L.; Pope, P. B.; Bakken, L. R. Nitrous Oxide Respiring Bacteria in Biogas Digestates for Reduced Agricultural Emissions. *ISME J.* **2022**, *16* (2), 580–590. https://doi.org/10.1038/s41396-021-01101-x

(83) Hiis, E. G.; Vick, S. H. W.; Molstad, L.; Røsdal, K.; Jonassen, K. R.; Winiwarter, W.; Bakken, L. R. Unlocking Bacterial Potential to Reduce Farmland N2O Emissions. *Nature* **2024**, *630* (8016), 421–428. https://doi.org/10.1038/s41586-024-07464-3

(84) Barba, J.; Poyatos, R.; Vargas, R. Automated Measurements of Greenhouse Gases Fluxes from Tree Stems and Soils: Magnitudes, Patterns and Drivers. *Sci. Rep.* **2019**, *9* (1), 4005. https://doi.org/10.1038/s41598-019-39663-8

(85) Machacova, K.; Maier, M.; Svobodova, K.; Lang, F.; Urban, O. Cryptogamic Stem Covers May Contribute to Nitrous Oxide Consumption by Mature Beech Trees. *Sci. Rep.* **2017**, *7* (1), 13243. https://doi.org/10.1038/s41598-017-13781-7

(86) Machacova, K.; Schindler, T.; Bréchet, L.; Mander, Ü.; Grams, T. E. E. Substantial Uptake of Nitrous Oxide (N2O) by Shoots of Mature European Beech. *Sci. Total Environ.* **2024**, *934*, 173122. https://doi.org/10.1016/j.scitotenv.2024.173122

(87) Daniel, W.; Stahl, C.; Burban, B.; Goret, J.-Y.; Cazal, J.; Richter, A.; Janssens, I. A.; Bréchet, L. M. Tree Stem and Soil Methane and Nitrous Oxide Fluxes, but Not Carbon Dioxide Fluxes, Switch Sign along a Topographic Gradient in a Tropical Forest. *Plant Soil* **2023**, *488* (1–2), 533–549. https://doi.org/10.1007/s11104-023-05991-y

(88) Ricky S. Kong; Binyam Tedla; Michael Schulz; Jean-Marie Sobze. *A Review of Lichen Transplant Studies and Methods*; Northern Alberta Institute of Technology Centre for Boreal Research: Peace River, AB, 2022.

https://arckp.ca/data/Lichen%20Literature%20Review%20%28Final%29.pdf (accessed 2025-12-05)

(89) Amadi, C. C.; Farrell, R. E.; Van Rees, K. C. J. Greenhouse Gas Emissions along a Shelterbelt-Cropped Field Transect. *Agric. Ecosyst. Environ.* **2017**, *241*, 110–120. https://doi.org/10.1016/j.agee.2016.09.037

(90) Beaudette, C.; Bradley, R. L.; Whalen, J. K.; McVetty, P. B. E.; Vessey, K.; Smith, D. L. Tree-Based Intercropping Does Not Compromise Canola (*Brassica Napus* L.) Seed Oil Yield and Reduces Soil Nitrous Oxide Emissions. *Agric. Ecosyst. Environ.* **2010**, *139* (1), 33–39. https://doi.org/10.1016/j.agee.2010.06.014

(91) Luo, J.; Beule, L.; Shao, G.; Veldkamp, E.; Corre, M. D. Reduced Soil Gross N_2 O Emission Driven by Substrates Rather Than Denitrification Gene Abundance in Cropland Agroforestry and Monoculture. *J. Geophys. Res. Biogeosciences* **2022**, *127* (3), e2021JG006629. https://doi.org/10.1029/2021JG006629

(92) Fowler, D.; Coyle, M.; Skiba, U.; Sutton, M. A.; Cape, J. N.; Reis, S.; Sheppard, L. J.; Jenkins, A.; Grizzetti, B.; Galloway, J. N.; Vitousek, P.; Leach, A.; Bouwman, A. F.; Butterbach-Bahl, K.; Dentener, F.; Stevenson, D.; Amann, M.; Voss, M. The Global Nitrogen Cycle in the Twenty-First Century. *Philos. Trans. R. Soc. B Biol. Sci.* **2013**, *368* (1621), 20130164. https://doi.org/10.1098/rstb.2013.0164

(93) Bennett, E. M.; Murray, J. W.; Isalan, M. Engineering Nitrogenases for Synthetic Nitrogen Fixation: From Pathway Engineering to Directed Evolution. *Biodesign Res.* **2023**, *5*, 0005. https://doi.org/10.34133/bdr.0005

(94) Burén, S.; Rubio, L. M. State of the Art in Eukaryotic Nitrogenase Engineering. *FEMS Microbiol. Lett.* **2018**, *365* (2), fnx274. https://doi.org/10.1093/femsle/fnx274

(95) Wan, S.; Greenham, T.; Goto, K.; Mottiar, Y.; Johnson, A. M.; Staebler, J. M.; Zaidi, M. A.; Shu, Q.; Altosaar, I. A Novel Nitrous Oxide Mitigation Strategy: Expressing Nitrous Oxide Reductase from Pseudomonas Stutzeri in Transgenic Plants. *Can. J. Plant Sci.* **2014**, *94* (6), 1013–1023. https://doi.org/10.4141/cjps2013-141

(96) Demone, J. J.; Wan, S.; Nourimand, M.; Hansen, A. E.; Shu, Q.; Altosaar, I. New Breeding Techniques for Greenhouse Gas (GHG) Mitigation: Plants May Express Nitrous Oxide Reductase. *Climate* **2018**, *6* (4), 80. https://doi.org/10.3390/cli6040080

(97) Liu, F.; Zhao, Z.; Fernie, A. R.; Zhang, Y. Towards Establishing Functional Nitrogenase Activities within Plants. *Trends Biotechnol.* **2025**, *43* (11), 2698–2708. https://doi.org/10.1016/j.tibtech.2025.04.020

(98) Spatola Rossi, T.; Tolmie, A. F.; Nichol, T.; Pain, C.; Harrison, P.; Smith, T. J.; Fricker, M.; Kriechbaumer, V. Recombinant Expression and Subcellular Targeting of the Particulate Methane Monooxygenase (pMMO) Protein Components in Plants. *Sci. Rep.* **2023**, *13* (1), 15337. https://doi.org/10.1038/s41598-023-42224-9

(99) Wan, S.; Mottiar, Y.; Johnson, A. M.; Goto, K.; Altosaar, I. Expression of the Nos Operon Proteins from Pseudomonas Stutzeri in Transgenic Plants to Assemble Nitrous Oxide Reductase. *Transgenic Res.* **2012**, *21* (3), 593–603. https://doi.org/10.1007/s11248-011-9555-1

(100) Santos, M. S.; Nogueira, M. A.; Hungria, M. Microbial Inoculants: Reviewing the Past, Discussing the Present and Previewing an Outstanding Future for the Use of Beneficial Bacteria in Agriculture. *AMB Express* **2019**, *9*, 205. https://doi.org/10.1186/s13568-019-0932-0

(101) Grooms, L. *Investing in inoculants*. Farm Progress. https://www.farmprogress.com/soybean/investing-in-inoculants (accessed 2025-12-07)

(102) Tesfaye, M.; Powe, M.; Morris, P. R. *What's the Big Deal About Biologicals?*; Indigo Ag: No-Till Farmer, 2022. https://www.no-tillfarmer.com/articles/11990-whats-the-big-deal-about-biologicals-webinar (accessed 2025-12-07)

(103) Successful Farming. *Envita Now Available for Foliar Application in Corn*. https://www.agriculture.com/crops/corn/envita-now-available-for-foliar-application-in-corn (accessed 2025-12-07)

(104) Gil Gullickson. *Helpful or Overhyped: Uncertainty Exists With Biologicals*. BioConsortia. https://bioconsortia.com/helpful-or-overhyped-uncertainty-exists-with-biologicals/ (accessed 2025-12-07)

(105) Link, E. *Utrisha N Biological Nitrogen Supplement for Organic Corn*; Practical Farmers of Iowa, 2024. https://practicalfarmers.org/research/utrisha-n-biological-nitrogen-supplement-for-organic-corn/ (accessed 2025-12-07)

(106) Ronalds, I.; Grant, L. *Tweedsmuir Lichen Restoration Trial: Year 1 Report*; Ministry of Forests, Lands, Natural Resource Operations, and Rural Development, 2018.

(107) Jensen, B. B.; Burris, R. H. N2O as a Substrate and as a Competitive Inhibitor of Nitrogenase. *Biochemistry* **1986**, *25* (5), 1083–1088. https://doi.org/10.1021/bi00353a021

(108) Fulweiler, R. W.; Berberich, M. E.; Rinehart, S. A.; Taylor, J. M.; Kelly, M. C.; Ray, N. E.; Oczkowski, A.; Balint, S. J.; Geisser, A. H.; Mahoney, C. R.; Benavides, M.; Church, M. J.; Loeks, B.; Newell, S. E.; Olofsson, M.; Oppong, J. C.; Roley, S. S.; Vizza, C.; Wilson, S. T.; Groffman, P. M.; Scott, J. T.; Marcarelli, A. M. A Global Dataset of Nitrogen Fixation Rates across Inland and Coastal Waters. *Limnol. Oceanogr. Lett.* **2025**, *10* (3), 412–429. https://doi.org/10.1002/lol2.10459

(109) Vieten, B.; Conen, F.; Seth, B.; Alewell, C. The Fate of N_2O Consumed in Soils. *Biogeosciences* **2008**, *5* (1), 129–132. https://doi.org/10.5194/bg-5-129-2008

(110) Southern Queensland Landscapes. *New use for old technology set to make measuring soil carbon more affordable*. https://www.sqlandscapes.org.au/new-use-for-old-technology-set-to-make-measuring-soil-carbon-more-affordable (accessed 2025-12-09)

(111) Butterbach-Bahl, K.; Sander, B. O.; Pelster, D.; Díaz-Pinés, E. Quantifying Greenhouse Gas Emissions from Managed and Natural Soils. In *Methods for Measuring Greenhouse Gas Balances and Evaluating Mitigation Options in Smallholder Agriculture*; Rosenstock, T. S., Rufino, M. C., Butterbach-Bahl, K., Wollenberg, L., Richards, M., Eds.; Springer International Publishing: Cham, 2016; pp 71–96. https://doi.org/10.1007/978-3-319-29794-1_4

(112) World Bank Group. *Global Solar Atlas*. https://globalsolaratlas.info/map (accessed 2025-12-14)

(113) Australian Government - Bureau of Meteorology. *Climate Averages Maps: Rainfall*. https://www.bom.gov.au/climate/maps/averages/rainfall/?period=dry&region=aus (accessed 2025-12-14)
